# Transcriptional profiling of Arabidopsis heat shock proteins and transcription factors reveals extensive overlap between heat and non-heat stress response pathways

**DOI:** 10.1186/1471-2164-8-125

**Published:** 2007-05-22

**Authors:** William R Swindell, Marianne Huebner, Andreas P Weber

**Affiliations:** 1 Department of Statistics and Probability, Michigan State University, East Lansing, MI 48824, USA; 2 Department of Plant Biology, Michigan State University, East Lansing, MI 48824, USA

## Abstract

**Background:**

The heat shock response of *Arabidopsis thaliana *is dependent upon a complex regulatory network involving twenty-one known transcription factors and four heat shock protein families. It is known that heat shock proteins (Hsps) and transcription factors (Hsfs) are involved in cellular response to various forms of stress besides heat. However, the role of Hsps and Hsfs under cold and non-thermal stress conditions is not well understood, and it is unclear which types of stress interact least and most strongly with Hsp and Hsf response pathways. To address this issue, we have analyzed transcriptional response profiles of *Arabidopsis *Hsfs and Hsps to a range of abiotic and biotic stress treatments (heat, cold, osmotic stress, salt, drought, genotoxic stress, ultraviolet light, oxidative stress, wounding, and pathogen infection) in both above and below-ground plant tissues.

**Results:**

All stress treatments interact with Hsf and Hsp response pathways to varying extents, suggesting considerable cross-talk between heat and non-heat stress regulatory networks. In general, Hsf and Hsp expression was strongly induced by heat, cold, salt, and osmotic stress, while other types of stress exhibited family or tissue-specific response patterns. With respect to the Hsp20 protein family, for instance, large expression responses occurred under all types of stress, with striking similarity among expression response profiles. Several genes belonging to the Hsp20, Hsp70 and Hsp100 families were specifically upregulated twelve hours after wounding in root tissue, and exhibited a parallel expression response pattern during recovery from heat stress. Among all Hsf and Hsp families, large expression responses occurred under ultraviolet-B light stress in aerial tissue (shoots) but not subterranean tissue (roots).

**Conclusion:**

Our findings show that Hsf and Hsp family member genes represent an interaction point between multiple stress response pathways, and therefore warrant functional analysis under conditions apart from heat shock treatment. In addition, our analysis revealed several family and tissue-specific heat shock gene expression patterns that have not been previously described. These results have implications regarding the molecular basis of cross-tolerance in plant species, and raise new questions to be pursued in future experimental studies of the *Arabidopsis *heat shock response network.

## Background

The heat shock response network of *Arabidopsis thaliana *involves temperature perception mechanisms, an intricate array of signal transduction networks, and twenty-one known transcription factors that activate heat shock proteins and other heat-stress related genes [1-3]. The availability of genome sequence data has considerably advanced our understanding of this heat shock response pathway, as well as the molecular basis of regulatory networks that underlie other forms of environmental stress in *Arabidopsis *(e.g., cold, salinity, drought). One result of this development has been increased recognition of the cross-talk or overlap that exists among cellular responses to different environmental stress treatments [4-8]. In this respect, heat shock proteins (and their associated transcription factors) are of special interest. Heat shock proteins are molecular chaperones that regulate the folding, localization, accumulation, and degradation of protein molecules in both plant and animal species [9]. Heat shock proteins are thus believed to play a broad role in many cellular processes, which may impart a generalized role in tolerance to multiple environmental stress treatments apart from heat stress. Understanding the role of heat shock proteins under cold and non-thermal stress conditions may therefore provide insight into multiple stress tolerance mechanisms [10]. This may be of considerable importance for improving the production of agriculturally important crop species under field conditions, which are best characterized as an interaction of several different types of stress, rather than just a single stress treatment in isolation [7].

The *Arabidopsis *heat shock proteins (Hsps) and transcription factors (Hsfs) have been well characterized on the basis of genome sequence information [1,11-14]. In addition to the twenty-one known transcription factors [1], the *Arabidopsis *heat shock response is partly mediated by thirteen Hsp20 proteins [11], eighteen Hsp70 proteins [12], seven Hsp90 proteins [13], and up to eight members of the Hsp100 protein family [14]. The molecular pathways leading to Hsp expression are not entirely understood [2], but involve temperature perception mechanisms coupled with multiple signal transduction pathways [3], which together lead to the activation of Hsfs that induce expression of heat shock genes by binding to heat shock elements [15]. There are several levels at which this molecular pathway may overlap with those underlying response to cold and non-thermal stress treatments. However, since Hsps play a uniquely broad role in cellular processes, Hsps are particularly likely to underlie interactions between heat and non-heat stress response pathways. A role of Hsps in cellular response to cold and non-heat stress treatments, for instance, has been supported by several gene expression studies. In *Arabidopsis *and other plant species, various Hsps have been induced by low temperature [16], osmotic stress [17], salt [18], oxidative stress [19-22], desiccation [23], exposure to intense light [24,25], wounding [4], and heavy metal exposure [26].

While a number of studies have shown that Hsp expression can be induced under cold and non-thermal stress treatments, no comparative analysis has been carried out to identify which particular stress treatments are the weakest and strongest inducers of Hsp expression. It therefore remains unclear which stress-response pathways overlap most extensively with this important part of the *Arabidopsis *heat shock regulatory network. If the primary stress conditions interacting with Hsp response pathways can be identified, it would be of considerable interest to understand how Hsfs and Hsps contribute to tolerance under such stress conditions. The physiological role of Hsfs and Hsps in promoting tolerance may differ depending on the nature of the stress imposed upon the cell. Heat stress, for instance, leads directly to denaturation of cellular proteins. It is therefore clear how molecular chaperone activity may contribute to high temperature tolerance via prevention of deleterious protein conformations and elimination of non-native aggregations. With respect to cold and non-thermal stress treatments, however, the impact on cellular protein conformations is less direct and not as well understood. The role of Hsps as molecular chaperones, therefore, may not strictly parallel their function under heat stress, and it is possible that their cellular function extends beyond the chaperone activity that has been well characterized *in vitro *[27,28]. One possibility, for example, is that Hsps limit damage resulting from accumulation of reactive oxygen species, which are generated as messengers and elements of signal transduction pathways under a wide range of stress conditions [29]. In both plant and animal species, for instance, there is evidence to suggest that Hsps protect against reactive oxygen species [30-38]. This hypothesis is particularly intriguing in light of the considerable interconnectivity that exists between heat shock and oxidative stress response pathways in plant species [21,22,39,40].

DNA microarray technology offers a promising approach for better understanding the functional role of *Arabidopsis *heat shock proteins and transcription factors under both heat and non-heat stress conditions. Recently, a number of genome-wide microarray datasets have been generated and made publicly available by the AtGenExpress consortium [41]. These resources provide an opportunity to profile Hsf and Hsp expression over a wide range of stress conditions simultaneously. In this study, we utilized AtGenExpress datasets to analyze transcriptional responses of *Arabidopsis *Hsfs and Hsps to a total of ten different abiotic and biotic stress treatments (cold, osmotic stress, salt, drought, genotoxic stress, ultraviolet light, oxidative stress, wounding, high temperature, and pathogen infection). For all abiotic stress treatments, we analyzed expression measurements generated from both below (root) and above-ground (shoots) tissue samples, while with respect to pathogen infection treatment, expression measurements generated from leaf tissue were considered. In all treatments, expression measurements obtained at up to six different time points of stress exposure (0.5, 1, 3, 6, 12, and 24 hours). With respect to each of five protein families (Hsf, Hsp20, Hsp70, Hsp90, and Hsp100), we evaluated whether expression responses of each family to each stress were significantly large in comparison to other *Arabidopsis *genes. This analysis provided indication of which types of stress interacted most and least with each protein family. In addition, we characterized Hsf and Hsp stress-response patterns at the level of protein families, as well as among individual genes within protein families. This allowed identification of family-level expression patterns under each stress, gene sub-groups within families exhibiting similar expression patterns, and individual Hsf/Hsp genes with large expression responses to multiple stress treatments.

## Results

An overview of how strongly each stress treatment impacted expression levels for each heat shock gene family is provided in Table 1. To compare the effect of each stress, a summary statistic was developed (*T*) that represents the median level of fold-change induced by each stress among members of a given protein family (see Equation 2 in Methods). For the Hsp20, Hsp70 and Hsp90 protein families, the largest expression responses were associated with the high temperature treatment, with median levels of fold-change in each family (*T*) ranging from one to above four (Table 1). However, for the Hsf and Hsp100 protein families, the largest magnitude expression responses were associated with osmotic stress (Table 1). For each stress, it was of interest to determine whether median-level expression responses of each gene family were large in comparison to all other genes represented on the ATH1 array. A resampling procedure was therefore carried out to evaluate the likelihood of observed *T *statistics under a null hypothesis of random sampling from the genome (see Table 1 caption and Methods). Significant *T *statistics were found with respect to each type of stress we considered, indicating that for one or more protein families, each stress induced expression responses that were large in comparison to other *Arabidopsis *genes. High temperature was associated with a significant *T *statistic for all protein families except Hsp100 in both roots and shoots. The second strongest elicitor of expression responses was oxidative stress, since it was associated with a significant *T *statistic for most families in both tissue types.

Protein families exhibiting strong expression responses to many stress treatments exhibit a generalized expression response pattern. The Hsf and Hsp20 were associated with the most stress-general expression patterns, since for both families, *T *was significant for nearly all types of stress (see Table 1). In contrast, the Hsp70, Hsp90, and Hsp100 families were not so widely responsive across stress treatments. Expression response patterns associated with each protein family are described in the following sections. Two additional files related to these results are available online. Additional file 1 contains summary results from differential expression analyses, heat maps of clustering solutions, and raw signal intensities of Hsf and Hsp genes (present/absent calls). Additional file 2 contains expression response profiles for each individual Hsf and Hsp gene under the stress conditions we examined.

### Heat shock transcription factors

**Figure 1:**
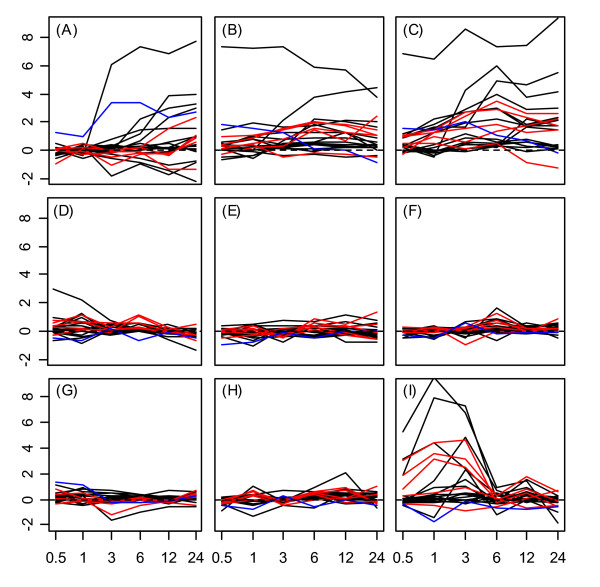
**Heat shock transcription factor expression response profiles in roots**. Expression response profiles associated with heat shock transcription factors under (A) cold stress, (B) osmotic stress, (C) salt stress, (D) drought, (E) genotoxic stress, (F) oxidative stress, (G) ultraviolet-b light, (H) wounding, and (I) heat in root tissue. Class A, B, and C transcription factors are represented by black, red, and blue lines, respectively. The horizontal axis of each subplot corresponds to time points at which gene expression measurements were obtained under each stress treatment (0.5, 1, 3, 6, and 12 hrs.). The vertical axis of each subplot indicates the log_2 _fold-change associated with each Hsf under a given stress treatment.

**Figure 2:**
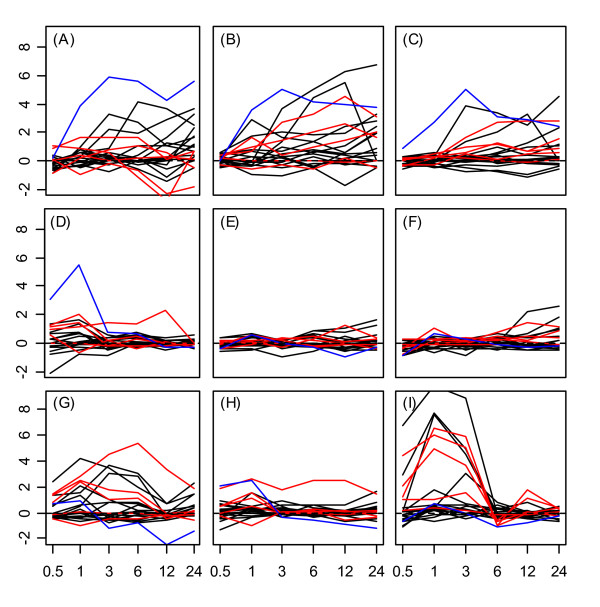
**Heat shock transcription factor expression response profiles in shoots**. Expression response profiles associated with heat shock transcription factors in shoot tissue. See Figure 1 caption.

Heat shock transcription factors were most strongly upregulated under heat, cold, osmotic, and salt stress treatments. Figure 1 displays gene expression response profiles for all Hsf genes in roots, while Figure 2 displays response profiles of Hsf genes in shoots. In both tissues, expression responses to cold, osmotic, and salt treatment primarily occur over the late stages of stress exposure between 6 and 24 hours (see Figs. 1 and 2, parts A – C). This pattern contrasts with that observed under heat stress treatment, in which Hsfs were strongly up-regulated during early stages of stress exposure, with the effect diminishing after the heat stress was removed beyond the 6 hr. time point (Figs. 1 and 2, part I). A general trend among all five heat shock gene groups was a difference between the effects of UV-B stress in shoot tissue in comparison to root tissue. With respect to the Hsf genes, UV-B stress induced strong up-regulation over most time points in shoot tissue (Fig 2G), but yielded comparatively low gene expression responses in roots (Fig. 1G).

Considerable variation was observed among expression response patterns associated with individual Hsf genes. To discern which Hsf genes were the least and most stress-responsive across all stress treatments, we ranked genes according to an index (*d*) (see Table 2). The value of *d *represents the mean proportion of time points, among all stress and tissue types we considered, at which a gene was differentially expressed (see Methods). Highly stress-responsive genes were associated with large values of *d*, while genes less responsive to stress were associated with low values of *d*. The seven least-stress responsive Hsf genes were all class A type Hsfs, and were associated with values of *d *less than or equal to 0.167. The most stress responsive Hsf gene, in contrast, was the one class C transcription factor in the Hsf family (HsfC1, *d *= 0.456). Cluster analysis using the HOPACH algorithm (see Methods) identified three multi-member clusters of Hsf genes with respect to stress-responses across all tissue-treatment-time combinations (stress-clusters 451, 452, and 470) (see Table 2). For comparison, the Hsfs were also clustered with respect to their expression patterns across the developmental series conditions analyzed by Schmid et al. (2005) [41] (see Table 2). Members of developmental-cluster 60 (see Table 2) exhibited a pattern of tissue-specificity that was found among certain genes from each of the four Hsp families. The expression pattern was characterized by strong upregulation specific to seed stages 6 – 10 (ATGE conditions 81 – 84), roots (17 days) (ATGE condition 9), flowers stage 12 (ATGE conditions 34 – 37), and flowers stage 15 (ATGE conditions 41 – 45) (see section 1C of additional file 1). Among all Hsfs, two clustered together with respect to both the developmental and stress datasets (HsfB2a and HsfB2b), strongly suggesting co-regulation of these genes.

### Hsp20 protein family

**Figure 3:**
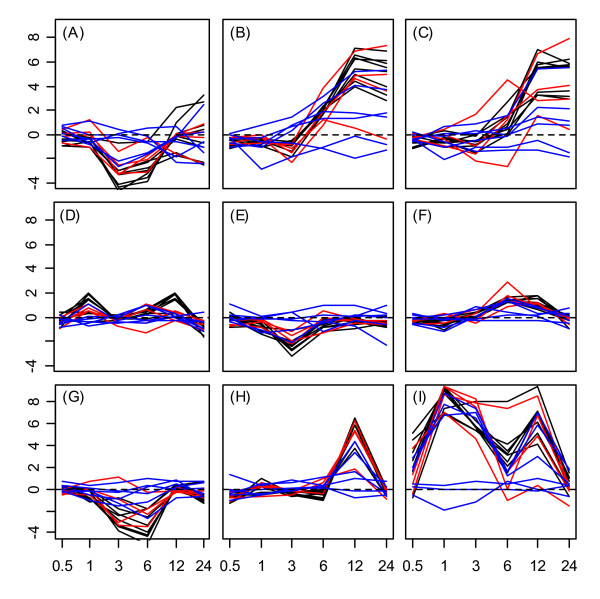
**Hsp20 expression response profiles in roots**. Expression response profiles associated with Hsp20 proteins under (A) cold stress, (B) osmotic stress, (C) salt stress, (D) drought, (E) genotoxic stress, (F) oxidative stress, (G) ultraviolet-b light, (H) wounding, and (I) heat in root tissue. The cytoplasmic/nuclear Hsp20s (classes I – III) are represented by black lines. Plastidial, endoplasmic reticulum, and mitochondrial Hsp20s (classes P, ER, and M) are represented by red lines. Class I and Class P related Hsp20s are indicated by blue lines. The horizontal axis of each subplot corresponds to time points at which gene expression measurements were available under each stress treatment (0.5, 1, 3, 6, and 12 hrs.). The vertical axis of each subplot indicates log_2 _fold-change under a given stress treatment. A dotted horizontal line in each plot indicates a log_2 _fold-change of zero (no expression response to stress).

**Figure 4:**
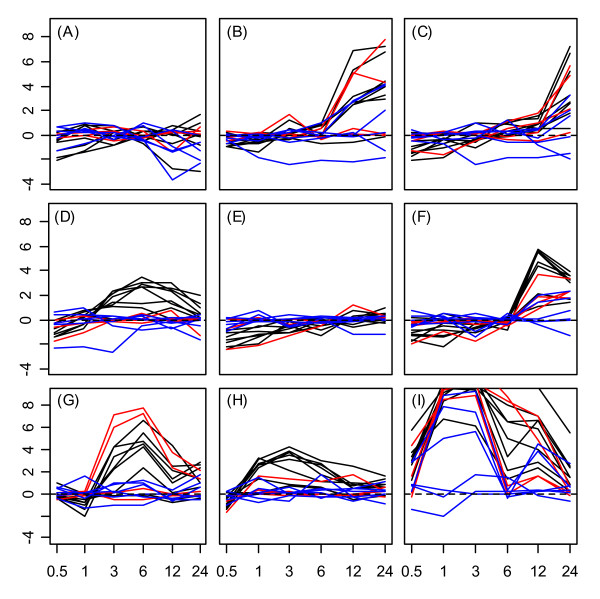
**Hsp20 expression response profiles in shoots**. Expression response profiles associated with Hsp20 proteins in shoot tissue.

The Hsp20 protein family exhibited the strongest overall responsiveness to environmental stress treatments, as well as the most cohesive family-level expression patterns among member genes. Expression response profiles are displayed in Figures 3 and 4 for all Hsp20 proteins in root and shoot tissues respectively. Tissue-specific patterns of Hsp20 stress-response can be discerned from a comparison of Figures 3 and 4. With respect to the UV-B treatment, for example, strong downregulation of Hsp20 genes occurred between the 3 – 6 hr. time points in roots (Fig. 3G). In shoots, however, UV-B stress induced strong upregulation over this same time period (Fig. 4G). With respect to the cold stress treatment, Hsp20 genes were downregulated between the 3 – 6 hr. time points in roots (Fig. 3A), while no such response pattern was associated with shoots (Fig. 4A). Tissue differences were also associated with wounding and heat stress, since expression response profiles of Hsp20 proteins exhibited different temporal dynamics in the two tissue types (see Figs. 3H, 3I, 4H and 4I).

**Figure 5:**
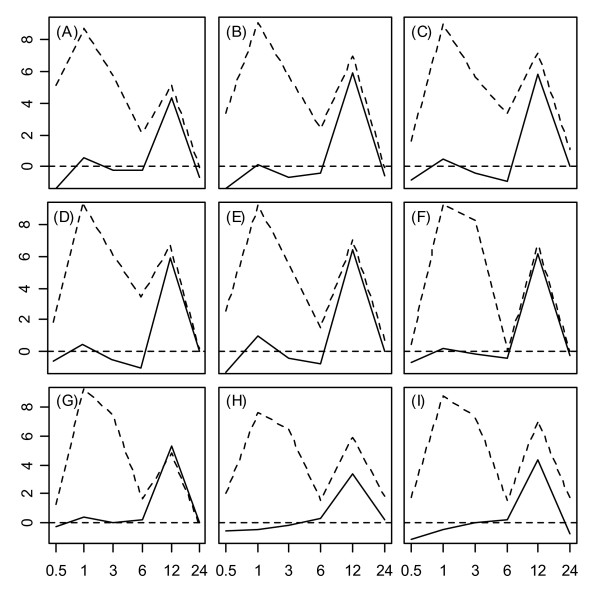
**Expression response profiles of select Hsp20 genes under wounding and heat stress treatments**. Expression response profiles of nine selected Hsp20 proteins under wounding (solid line) and heat (dotted line) treatments are shown. Subplots display response profiles associated with (A) 17.6A-CI, (B) 17.4-CI, (C) 17.6C-CI, (D) 17.6-CII, (E) 17.7-CII, (F) 25.4-P, (G) 23.6-M, (H) 15.7-CI(r), and (I) 26.5-P(r). The horizontal axis corresponds to time points at which gene expression measurements were obtained, while the vertical axis indicates the log_2 _fold-change. The dotted horizontal line in each plot indicates a log_2 _fold-change of zero (no expression response to stress). For the heat stress treatment, roots were exposed to heat until the 3 hr. time point, such that the 3–24 hr time interval represents a recovery period.

The expression responses of Hsp20 proteins under wounding and heat stress revealed surprising family-level patterns within root tissue. Nearly all Hsp20 proteins exhibited strong upregulation 12 hrs. following wounding of root tissue (see Fig. 3H). Under the heat treatment, Hsp20 upregulation also occurred at the 12 hr. time point (Fig 3I), which represented the heat stress recovery period (9 hrs. following cessation of heat stress). These expression responses during heat stress recovery were a unique aspect of the Hsp20 family, since generally, all other heat shock genes were responsive only while heat stress was directly applied (0.5 – 3 hrs.). For a number of Hsp20 genes, moreover, the 12 hr. upregulation under heat strongly coincided with that observed under the wounding stress treatment. This synchrony between expression response profiles under wounding and heat treatment in root tissue is evident from Figure 5, which displays response profiles of nine Hsp20 genes under wounding and heat stress treatments.

Most Hsp20 proteins exhibited strong expression responses to several types of stress. AtHsp14.2-P(r) exhibited the weakest overall responsiveness to stress (*d *= 0.096), while in contrast, AtHsp18.5-Cl(r) showed the strongest expression responses to stress treatments (*d *= 0.325) (see Table 3). Cluster analyses revealed strong similarities among Hsp20 genes with respect to stress-response patterns and the developmental series [41] (see section 2C of additional file 1). Members of stress-cluster 30 (AtHsp23.6-M, AtHsp25.4-P) and stress-cluster 42 (AtHsp26.5-P(r), AtHsp15.7-Cl(r), AtHsp22.0-ER) were highly responsive to stress treatments in root tissue. The other multi-gene stress-cluster (21) consisted of Hsp17 proteins entirely (AtHsp17.4-CI, AtHsp17.6-CII, AtHsp17.6B-CI, AtHsp17.6C-CI, AtHsp17.7-CII), and similar to stress-clusters 30 and 42, exhibited large expression responses to all stress treatments, except that strong responses were present in both roots and shoots. The members of all three of these stress-clusters, and most Hsp20 proteins in general, exhibited a similar expression profile among developmental stages (see section 2C of additional file 1). As among certain Hsfs, this developmental expression profile consisted of high expression levels with respect to roots (17 days), flowers stage 12, flowers stage 15, and seed stages 6 – 10. Three cytoplasmic/nuclear Hsp20 genes clustered together with respect to both developmental and stress datasets (AtHsp17.7-CII, AtHsp17.6C-Cl, AtHsp17.4-Cl).

### Hsp70, Hsp90, and Hsp100 protein families

**Figure 6:**
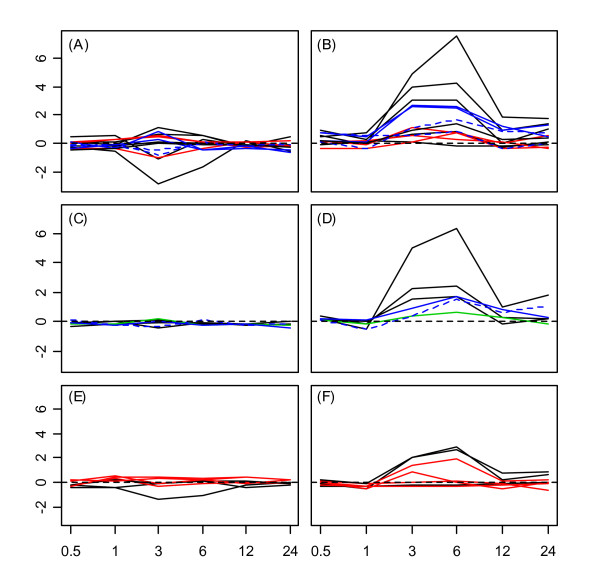
**Hsp70, Hsp90 and Hsp100 expression response profiles under ultraviolet-B light stress treatment**. Expression response profiles associated with all members of the Hsp70 family (A and B), Hsp90 family (C and D), and Hsp100 family (E and F). Profiles associated with root tissue are shown in A, C, and E, while expression response profiles associated with shoot tissue are shown in B, D, and F. The horizontal axis corresponds to time points at which genes expression measurements were obtained, while the vertical axis indicates the log_2 _fold-change. The dotted horizontal line in each plot indicates a log_2 _fold-change of zero (no expression response to UV-B light). Hsps were localized to the cytoplasm (black lines), plastid (red lines), chloroplast (green line), mitochondria (blue lines), or endoplasmic reticulum (dashed blue line).

**Figure 7:**
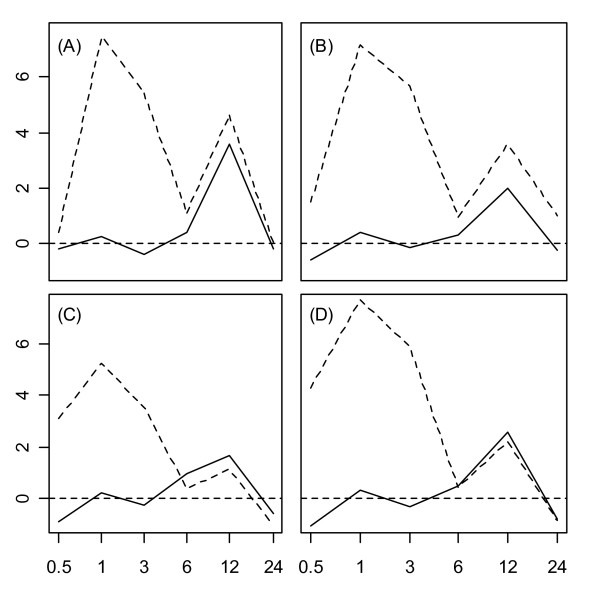
**Expression response profiles of selected Hsp70, Hsp90 and Hsp100 genes under wounding and heat stress treatments**. Expression response profiles of four selected proteins (Hsp70, Hsp90, or Hsp100) under wounding (solid line) and heat (dotted line) treatments are shown. Subplots display response profiles associated with (A) AtHsp70-5, (B) AtHsp70-8, (C) AtHsp90-1, and (D) AtHsp100-1. The horizontal axis corresponds to time points at which gene expression measurements were obtained, while the vertical axis indicates the log_2 _fold-change. The dotted horizontal line in each plot indicates a log_2 _fold-change of zero (no expression response to stress). For the heat stress treatment, roots were exposed to heat until the 3 hr. time point, such that the 3–24 hr time interval represents a recovery period.

The Hsp70, Hsp90, and Hsp100 protein families were generally associated with smaller magnitude expression responses across stress conditions. However, members of these families were stress-responsive, since differential expression occurred under most stress conditions for nearly every Hsp within these families (see sections 3B, 4B, and 5B of additional file 1). Members of Hsp70, Hsp90, and Hsp100 families were most strongly induced by heat, primarily over the early portion of the time course (0.5 -3 hrs.), although several genes within each family exhibited large responses to the cold, osmotic, and salt stress treatments (see additional file 2). The Hsp70, Hsp90, and Hsp100 families were all associated with a similar tissue-specific pattern under the UV-B stress condition (see Fig. 6). In particular, expression levels of member genes increased at the 3 – 6 hr. time points in shoot tissue, with little or no transcriptional induction in root tissue. In addition, the expression response pattern identified following wounding and heat stress in root tissue was also evident with respect to AtHsp70-5, AtHsp70-8, AtHsp100-1, and to a lesser extent, AtHsp90-1 (see Fig. 7).

The individual gene members of the Hsp70, Hsp90, and Hsp100 families are listed in tables 4, 5, and 6, respectively. On the basis of differential expression, these families contained both the least and most stress-responsive *Arabidopsis *Hsps. The most stress-responsive was AtHsp70-4 (*d *= 0.439), which was differentially expressed under all stress treatments, including all three time points of exposure to pathogen stress. In contrast, AtHsp100-2 was the least stress-responsive *Arabidopsis *Hsp (*d *= 0.009), and was differentially expressed with respect to just one time point under the heat stress treatment (see section 5B of additional file 1).

Clustering of Hsp90 genes with respect to stress-response patterns assigned five members to one group (AtHsp90-2, 4, 5, 6, and 7), since these genes were all associated with highly similar (and weak) expression response patterns in root tissue (see section 4C of additional file 1). The remaining AtHsp90-1 exhibited a strong expression response pattern distinct from all other AtHsp90 genes, and was therefore assigned to a singleton cluster (see Table 5). Within Hsp70 and Hsp100 families, clustering with respect to stress-response patterns identified few sub-groups among member genes (see Tables 4 and 6).

Various members of the Hsp70, Hsp90, and Hsp100 families were associated with the same developmental expression pattern found among certain Hsf and Hsp20 genes. This pattern was best exhibited by AtHsp70-4, AtHsp70-11, AtHsp90-1, and AtHsp100-1, all of which were highly expressed in roots (17 days), flowers stage 12, flowers stage 15, and seed stages 6 – 10 (see sections 3C, 4C, and 5C of additional file 1).

## Discussion

Heat shock proteins (Hsps) and transcription factors (Hsfs) are central components of the *Arabidopsis thaliana *heat shock regulatory network. It has long been recognized that these elements are also involved in response to cold and non-thermal stress treatments [9], but the types of stress that most strongly elicit Hsp/Hsf expression responses have not been identified, and the physiological role of these proteins under non-heat stress treatments is unclear. The findings of this study support the hypothesis that Hsps and Hsfs represent an intersection point between heat and non-heat stress response pathways. Our results indicate that, to varying extents, each of nine cold and non-thermal stress treatments interact with Hsfs and Hsps at the level of gene expression. Several prominent family-level expression response patterns were identified. These included highly similar stress-response profiles among Hsp20 proteins, a number of Hsps specifically upregulated 12 hours after wounding and during recovery following heat stress, and upregulation of heat shock genes to UV-B stress in shoot but not root tissue. Our findings raise important questions to be pursued in future experimental studies of the *Arabidopsis *heat shock response network.

Genome-wide transcriptional profiling allowed the expression of Hsf and Hsp genes under many stress conditions to be examined within the same context. This facilitated identification of which stressors interact with each protein family most strongly, which provides insight into the nature and degree of cross-talk that exists between heat and other forms of stress. The osmotic, cold, and salt treatments were among the strongest inducers of heat shock gene expression. These stress treatments induced expression responses of heat shock genes that were large in comparison to other *Arabidopsis *genes (see Table 1), and also large in an absolute sense, since these stressors induced strong fold-changes and differential expression of individual Hsf and Hsp genes (see Figs. 1, 2, 3, 4 and additional files). Expression response patterns were very similar under each of these treatments, with upregulation primarily occurring over the late stages of stress exposure (3 – 24 hours). Since osmotic, cold, and salt stress treatments are each believed to have a deleterious impact on cellular water potential [42,43], it is possible that their impact on heat shock genes is related to this common effect. In support of this notion, several previous studies in plant species have implicated Hsp20 proteins in tolerance to water stress treatments [17,44,45]. Among other stress treatments, wounding and UV-B stress induced moderately large expression responses of heat shock genes (with strong differences among families and between tissue types). The pathogen infection treatment was unique, since in contrast to other types of stress, it elicited strong expression responses among the Hsp70, Hsp90, and Hsp100 families, while most members of the Hsf and Hsp20 family were not responsive. Overall, drought and genotoxic stress treatments were associated with weak induction of heat shock genes, although some individual genes can be cited as an exception to this trend (e.g., HsfA8, AtHsp15.4-CI(r), AtHsp100-7).

The degree to which oxidative stress impacted heat shock gene expression is difficult to discern. In comparison to other *Arabidopsis *genes, all protein families (except Hsp90) exhibited large expression responses to oxidative stress (see Table 1). However, since genomic expression responses to oxidative stress were generally small, absolute fold-changes induced by oxidative stress were nonetheless of small magnitude. Only one transcription factor, for instance, was differentially expressed under oxidative stress (HsfA1d). These results were surprising, since there is considerable evidence that heat shock transcription factors can function as reactive oxygen species sensors in plants [40], and extensive interactions have been identified between heat and oxidative stress molecular pathways [33,38,39,46,47]. Moreover, since the generation of reactive oxygen species is a general response under many types of stress [29], Hsf activation by reactive oxygen species may provide the best hypothesis to explain why heat shock genes are induced by so many stress treatments. In view of this, an important factor to consider is the means by which oxidative stress was experimentally induced. For data we analyzed, oxidative stress was induced by exogenous application of methyl viologen, which is a generator of superoxide anion radical [48]. The impact of this reactive oxygen species on heat shock gene expression may differ from that of others [49,50]. In a recent study, for instance, Gadjev et al. (2006) [50] demonstrated that among genes upregulated more than two-fold under heat stress, relatively few were responsive to superoxide anion radical, while most were instead responsive to the singlet oxygen reactive oxygen species. These considerations suggest that, although the oxidative stress treatment examined by this study may not have had a strong impact on heat shock genes, the production of different types of reactive oxygen species (e.g., H_2_O_2_), leading to Hsf activation and consequently Hsp expression, remains a pathway through which cellular responses to heat and other forms of stress may be linked.

Heat shock transcription factors are of fundamental importance to understanding stress response networks, since these proteins coordinate the expression of Hsps and other stress-responsive genes. The *Arabidopsis *Hsf family is larger than that which has been described in any animal system [1], and at present, no single Hsf has been identified as a primary trigger of the heat shock response. The emerging picture is one of considerable complexity, with extensive interactions among individual Hsfs and sensitivity to a diverse range of environmental signals [40]. We found that seven Hsfs (six class A, one class B) exhibited very weak expression responses to heat and all other stress conditions (see Table 2, stress-clusters 451 and 452), while the remaining 14 Hsfs were strongly induced by several stress treatments. The most distinctive expression response patterns we observed were associated with HsfA6b and HsfC1 (see additional file 2). In root tissue, HsfA6b exhibited approximately five-fold induction to salt and osmotic treatments across all time points of gene expression measurement (0.5 – 24 hours). This pattern contrasted with that observed among other Hsfs, most of which responded to salt and osmotic stress over the late stages of stress exposure only. This early response of HsfA6b to salt and cold treatments was, in fact, unique among all the heat shock genes that we examined, suggesting that HsfA6b may interact with elements outside of the Hsf/Hsp response pathway. On the basis of differential expression analysis, HsfC1 was the most stress-responsive of all Hsfs. Among all treatments and tissues that we examined, this transcription factor was, on average, differentially expressed with respect to nearly half of the time points at which gene expression was measured. This strong expression response pattern is particularly noteworthy in light of the large structural dissimilarities between HsfC1 and all other *Arabidopsis *Hsfs [1].

The Hsp20 family exhibited the most stress-general expression response pattern of all the protein groups that we examined. Our results therefore suggest that this protein family is of potential importance as a factor contributing to multiple stress tolerance in plant species. These findings are also consistent with those of previous studies, which have found that certain Hsp20 proteins are involved in cellular responses to a wide variety of environmental treatments besides heat, such as alcohol [51], cold [16], heavy metals [52-55], osmotic stress [17], desiccation [56], and oxidative stress [38]. At present, little is known regarding how Hsp20 proteins are integrated with molecular networks that underlie cellular responses to these stress treatments. Increasingly, it has been recognized that Hsp20 proteins can engage in a wide range of cellular processes under stress, including ATP-independent stabilization of substrate proteins undergoing conformational disruption [55], or associating with lipid molecules to regulate fluidity of the membrane structure [57]. This latter function suggests that Hsp20s could be involved in the perception of stressful stimuli leading to the activation of signal transduction pathways. Under temperature extremes, the role of membrane fluidity as a means of stress perception and activation of signal transduction pathways has been well established [2]. However, since non-thermal stressors may also alter membrane fluidity or lead to various types of membrane damage, interactions of Hsp20s with membranes could partly account for the overall stress-responsiveness of the Hsp20 family.

A striking aspect of the Hsp20 family was the similarity among the expression response patterns of member genes. This similarity was demonstrated by our clustering analysis (see section 2C of additional file 1), which interestingly, revealed a cluster of five 17 kDa Hsp20 proteins that included both class I and II nuclear/cytosolic proteins. This result is consistent with findings of previous studies, which have identified functional similarities between class I and II Hsp20s [58], despite marked differences between the amino acid sequences of the two classes [59]. If analysis is restricted to stress responses occurring in the root tissue type only, the overall homology of Hsp20 expression response patterns is considerably enhanced. In root tissue, expression patterns of 17 kDa Hsp20s are very similar to those of the 18 – 20 kDa Hsp20s, including those localized to the mitochondria and endoplasmic reticulum (see section 2C of additional file 1). The similarity of expression patterns among the Hsp20 proteins may reflect shared induction mechanisms, and possibly extensive coordination among Hsp20s as cellular chaperones, such as that observed during the formation of heat-stress granules [60]. Shared induction mechanisms among Hsp20 proteins may include accumulation of denatured proteins in the cytoplasm [2], generation of reactive oxygen species [40], or changes in membrane lipid composition and fluidity [57]. These processes are thought to be upstream signals leading to the activation of critical Hsfs, which are most likely the direct inducers of Hsp20 expression under stress.

A number of Hsps were upregulated 12 hours after wounding, with a parallel expression response pattern during recovery from heat stress in root tissue. While the majority of these proteins were members of the Hsp20 family (see Fig. 5), some members of the Hsp70, Hsp90, and Hsp100 families also exhibited this distinctive expression pattern (see Fig. 7). The upregulation of multiple Hsps following wounding and during heat stress recovery has not been previously documented in *Arabidopsis *or other plant species, and is therefore an important finding of this study. The first indication that Hsps are involved in the wounding response pathway was provided by the study of Cheong et al. (2002) [4], in which the effect of wounding on expression levels of 8,200 *Arabidopsis *genes was surveyed in leaf tissue. Cheong et al. (2002) [4] identified one Hsf upregulated 0.5 hours following wounding (AtHsfA4a), along with another upregulated both 0.5 and 6 hours after wounding (AtHsfB1). In addition, several Hsp70 proteins were upregulated 6 hours after wounding, as well as two 17 kDa sHSPs (AtHsp17.8-CII and AtHsp17.7-CII). In our study, the most interesting wounding-response patterns occurred in root tissue, but our results are consistent with those of [4], since Hsp upregulation also occurred after wounding in aerial shoot tissue. Overall, our findings suggest that Hsp involvement in wounding response is greater than previously recognized, and by profiling Hsps simultaneously under multiple types of stress, our results show that late wound-responsive genes are also expressed during recovery from heat stress. These results point to a broad role of some Hsps during stress recovery or acclimation. Following wounding of plant tissue, both local and systemic signals are generated that coordinate defense responses aimed at limiting further injury (e.g., by pathogen) [61]. Similarly, following exposure to high temperatures, plants exhibit increased tolerance or hardening to limit damage resulting from future temperature elevations [2]. The functional role of Hsps upregulated as part of this post-wounding and post-heat stress response is unclear and warrants further investigation. With respect to heat stress recovery, one recent study found that mutant plants lacking a 32 kDa heat shock associated protein (Hsa32) exhibited an elevated decay in thermotolerance following exposure to heat stress [62].

Ultraviolet-B radiation resulted in upregulation of heat shock proteins and transcription factors in shoots, but did not have this effect in root tissue. This distinction between aerial and subterranean tissue types was most marked with respect to the Hsp20 group, in which nearly all Hsp20s were upregulated in shoots and downregulated in roots. Similar to other stress treatments, exposure to ultraviolet-B light has been associated with the production of reactive oxygen species [63,64]. Specifically, ultraviolet light stress has been found to increase cellular concentrations of H_2_O_2 _[65], which has been thought to activate Hsf expression [40], especially that of HsfA4a and HsfA8 [47]. We found that both HsfA4a and HsfA8 were strongly induced by UV-B stress in shoots but not in roots (see additional file 2). These observations are consistent with the notion that Hsp expression in shoots results from UV-B induced activation of Hsfs, possibly HsfA4a and HsfA8, with the generation of H_2_O_2 _as an intermediary signal. Given the tissue-specific effect we observed, however, the generation of H_2_O_2 _could be dependent upon interactions between UV-B stress and photosynthetic processes taking place in chloroplast. In previous models, it has been suggested that UV-B generated reactive oxygen species are upstream components that act upon photosynthetic genes (i.e., H_2_O_2 _→ photosynthesis) [66,67]. Our results, however, suggest that the reverse is also plausible, in which photosynthetic processes are upstream components leading to the generation of reactive oxygen species under UV-B stress (i.e., photosynthesis → H_2_O_2_).

## Conclusion

It has recently been emphasized that the generation of agricultural varieties tolerant to a range of stress conditions should be a primary goal in biotechnological applications, since under field conditions, plants may encounter different types of stress in combination [7]. Focusing on overlapping elements among response pathways that underlie diverse forms of stress may advance our knowledge of cross-tolerance in plant species [68]. The *Arabidopsis *heat shock proteins and transcription factors exhibit expression responses under a wide range of stressful stimuli, and are therefore a natural model for developing our understanding of integration between regulatory networks associated with different kinds of stress. The findings of this study have identified which types of stress interact least and most strongly with Hsfs and each Hsp family at the transcriptional level. In addition, new family and tissue-specific expression response patterns have been uncovered. These patterns include concerted expression response profiles among Hsp20 proteins, specific upregulation of most Hsp20 proteins (some Hsp70, Hsp90, and Hsp100 proteins) twelve hours following wounding of root tissue, and with respect to all heat shock gene families, upregulation under ultraviolet-B light stress in aerial (shoots) but not subterranean tissue (roots). These results provide insight into the nature and degree of cross talk between heat and non-heat stress conditions, and represent a basis for further experimental investigations into the involvement of Hsf and Hsp proteins under cold and non-thermal stress.

## Methods

### Microarray datasets

All microarray data analyzed in this study were generated using the ATH1 Affymetrix microarray platform [69,70], with expression estimates obtained by gcRMA normalization [71]. A total of 22,810 probes were included on the ATH1 platform, along with 64 control probes not corresponding to *Arabidopsis *genes. Our analysis is therefore based on a total of 22,746 genes, representing approximately 80% of all known *Arabidopsis *genes [41]. Abiotic stress and pathogen series expression datasets were downloaded from AtGenExpress [72]. Complete protocols associated with these data can be obtained from TAIR (submission numbers: ME00325, ME00326, ME00327, ME00328, ME00329, ME00330, ME00338, ME00339, ME00340, ME00342) [73]. In brief, the abiotic stress series data consists of gene expression measurements performed on *Arabidopsis thaliana *(col-0) roots and shoots under a benign control condition and nine environmental stress conditions. Root samples consisted of all below-ground plant tissue, while shoot samples consisted of all above-ground green tissues (including leaves). For each stress condition, expression measurements were obtained from 16 to 18-day old plants at six different time points of stress-exposure (1/2, 1, 3, 6, 12, and 24 hours). All expression measurements were performed with duplicate biological replications and no technical replications. Stress treatments included cold (4°C), osmotic stress (300 mM Mannitol), salt (150 mM NaCl), drought (15 min. dry air stream leading to 10% loss of fresh weight), genotoxic stress (1.5 μg/ml bleomycin, 22 μg/ml mitomycin), oxidative stress (10 μM methyl viologen), ultraviolet-B light stress (15 min. exposure, 1.18 W/m^2 ^Phillips TL40W/12), wounding (pin puncture), and high temperature (3 hrs. at 38°C followed by 21 hrs. recovery at 25°C). From the pathogen series dataset, we considered experiments involving *P. infestans *infection of 5-week old *Arabidopsis *leaves, along with corresponding control treatments in which H_2_O was applied to leaves. Only the treated leaf tissue was used in RNA extractions and microarray analyses (excluding all other green tissues). Expression measurements were obtained at each of three post-infection time points (6, 12, and 24 hours), with three biological replications at each time (no technical replication). Pathogen infections used 10^-8 ^cfu/ml in MgCl_2 _with 5 × 10^5 ^*P*. *infestans *spores applied to leaf surfaces.

### Heat shock proteins and transcription factors

The heat shock proteins and transcription factors analyzed in this study were selected based upon the genomic sequence analyses performed by [1,11-14]. Our analysis includes all of the twenty-one Hsfs identified by [1]. Several Hsps identified by the above-cited studies were not represented on the ATH1 array (AtHsp17.8-Cl, AtHsp70-12, AtHsp70-13, AtHsp70-14, AtHsp70-16, AtHsp70-18, AtHsp90-3, and AtHsp100-6), and therefore could not be included in this study. In total, our heat shock protein analysis is based upon 18 of 19 members of the Hsp20 family (12 sHsps and 6 related sHsp-like proteins), 13 of 17 members of the Hsp70 family (11 DnaK and 2 SSE subfamily), 6 of 7 members of the Hsp90 family, and 7 of 8 members of the Hsp100 family (AtHsp100-1 and six homologues). The expression response patterns of each Hsf and Hsp gene were analyzed with respect to nine abiotic stress treatments (applied to root and shoot tissue), in addition to pathogen infection treatment (applied to leaf tissue). In total, therefore, the expression response of each Hsf and Hsp was examined under 19 tissue-treatment combinations.

### Statistical analyses

The *T *statistic represents the median level of fold-change induced by a given stress treatment among members of a given protein family [74]. Let X¯ijkt
 MathType@MTEF@5@5@+=feaafiart1ev1aaatCvAUfKttLearuWrP9MDH5MBPbIqV92AaeXatLxBI9gBaebbnrfifHhDYfgasaacH8akY=wiFfYdH8Gipec8Eeeu0xXdbba9frFj0=OqFfea0dXdd9vqai=hGuQ8kuc9pgc9s8qqaq=dirpe0xb9q8qiLsFr0=vr0=vr0dc8meaabaqaciaacaGaaeqabaqabeGadaaakeaacuWGybawgaqeamaaBaaaleaacqWGPbqAcqWGQbGAcqWGRbWAcqWG0baDaeqaaaaa@33B1@ represent the mean gcRMA normalized expression intensity of the *i*th gene under the *j*th experimental treatment (abiotic stress or pathogen) within the *k*th tissue following *t *hours of stress exposure (*i *= 1...*N*, *j *= 1... 10, *k *= 1...3, and *t *= 0.5... 24). For every tissue-treatment-time combination, values of X¯ijkt
 MathType@MTEF@5@5@+=feaafiart1ev1aaatCvAUfKttLearuWrP9MDH5MBPbIqV92AaeXatLxBI9gBaebbnrfifHhDYfgasaacH8akY=wiFfYdH8Gipec8Eeeu0xXdbba9frFj0=OqFfea0dXdd9vqai=hGuQ8kuc9pgc9s8qqaq=dirpe0xb9q8qiLsFr0=vr0=vr0dc8meaabaqaciaacaGaaeqabaqabeGadaaakeaacuWGybawgaqeamaaBaaaleaacqWGPbqAcqWGQbGAcqWGRbWAcqWG0baDaeqaaaaa@33B1@ were associated with a corresponding control measurement designated as X¯i0kt
 MathType@MTEF@5@5@+=feaafiart1ev1aaatCvAUfKttLearuWrP9MDH5MBPbIqV92AaeXatLxBI9gBaebbnrfifHhDYfgasaacH8akY=wiFfYdH8Gipec8Eeeu0xXdbba9frFj0=OqFfea0dXdd9vqai=hGuQ8kuc9pgc9s8qqaq=dirpe0xb9q8qiLsFr0=vr0=vr0dc8meaabaqaciaacaGaaeqabaqabeGadaaakeaacuWGybawgaqeamaaBaaaleaacqWGPbqAcqaIWaamcqWGRbWAcqWG0baDaeqaaaaa@3342@ (*j *= 0). Log_2 _fold-changes (*M*) at each tissue-treatment-time combination were thus calculated as the difference between expression intensities in the *j*th stress treatment and corresponding control treatments.

Mijkt=X¯ijkt−X¯i0kt
 MathType@MTEF@5@5@+=feaafiart1ev1aaatCvAUfKttLearuWrP9MDH5MBPbIqV92AaeXatLxBI9gBaebbnrfifHhDYfgasaacH8akY=wiFfYdH8Gipec8Eeeu0xXdbba9frFj0=OqFfea0dXdd9vqai=hGuQ8kuc9pgc9s8qqaq=dirpe0xb9q8qiLsFr0=vr0=vr0dc8meaabaqaciaacaGaaeqabaqabeGadaaakeaacqWGnbqtdaWgaaWcbaGaemyAaKMaemOAaOMaem4AaSMaemiDaqhabeaakiabg2da9iqbdIfayzaaraWaaSbaaSqaaiabdMgaPjabdQgaQjabdUgaRjabdsha0bqabaGccqGHsislcuWGybawgaqeamaaBaaaleaacqWGPbqAcqaIWaamcqWGRbWAcqWG0baDaeqaaaaa@4325@

The average value of |*M*| occurring over all time points under a given tissue-treatment combination reflects the overall stress-responsiveness associated with a gene's expression profile. Letting this average value for gene *i *under treatment *j *in tissue *k *be represented by |M¯ijk⋅|
 MathType@MTEF@5@5@+=feaafiart1ev1aaatCvAUfKttLearuWrP9MDH5MBPbIqV92AaeXatLxBI9gBaebbnrfifHhDYfgasaacH8akY=wiFfYdH8Gipec8Eeeu0xXdbba9frFj0=OqFfea0dXdd9vqai=hGuQ8kuc9pgc9s8qqaq=dirpe0xb9q8qiLsFr0=vr0=vr0dc8meaabaqaciaacaGaaeqabaqabeGadaaakeaadaabdaqaaiqbd2eanzaaraWaaSbaaSqaaiabdMgaPjabdQgaQjabdUgaRjabgwSixdqabaaakiaawEa7caGLiWoaaaa@37A0@, a test statistic *T *was defined as the median value of |M¯ijk⋅|
 MathType@MTEF@5@5@+=feaafiart1ev1aaatCvAUfKttLearuWrP9MDH5MBPbIqV92AaeXatLxBI9gBaebbnrfifHhDYfgasaacH8akY=wiFfYdH8Gipec8Eeeu0xXdbba9frFj0=OqFfea0dXdd9vqai=hGuQ8kuc9pgc9s8qqaq=dirpe0xb9q8qiLsFr0=vr0=vr0dc8meaabaqaciaacaGaaeqabaqabeGadaaakeaadaabdaqaaiqbd2eanzaaraWaaSbaaSqaaiabdMgaPjabdQgaQjabdUgaRjabgwSixdqabaaakiaawEa7caGLiWoaaaa@37A0@ among the *n *gene members of a given protein family.

Tjk =mediani=1...n(|M¯ijk⋅|)
 MathType@MTEF@5@5@+=feaafiart1ev1aaatCvAUfKttLearuWrP9MDH5MBPbIqV92AaeXatLxBI9gBaebbnrfifHhDYfgasaacH8akY=wiFfYdH8Gipec8Eeeu0xXdbba9frFj0=OqFfea0dXdd9vqai=hGuQ8kuc9pgc9s8qqaq=dirpe0xb9q8qiLsFr0=vr0=vr0dc8meaabaqaciaacaGaaeqabaqabeGadaaakeaaieGacqWFubavdaWgaaWcbaGae8NAaOMae83AaSgabeaakiabbccaGiabb2da9maaxababaGaeeyBa0MaeeyzauMaeeizaqMaeeyAaKMaeeyyaeMaeeOBa4galeaajug4aiabdMgaPjabg2da9iabigdaXiabc6caUiabc6caUiabc6caUiabd6gaUbWcbeaakiabcIcaOiabcYha8jqbd2eanzaaraWaaSbaaSqaaiabdMgaPjabdQgaQjabdUgaRjabgwSixdqabaGccqGG8baFcqGGPaqkaaa@501E@

The magnitude of *T *reflects how large expression responses of a protein family are, on average, with respect to a given tissue-treatment combination. The significance of observed *T *statistics was evaluated under the null hypothesis that the *n *genes in each protein family are a random sample of the *N *= 22746 genes represented on the ATH1 array, versus the alternative that the *n *genes are a non-random sample yielding a *T *statistic larger than expected within a random sample. This hypothesis was evaluated by the following resampling procedure. With respect to each tissue-treatment combination and each protein family, a total of 10^3 ^random samples of *n *genes were drawn from among all *N *= 22746 genes, and the value of *T *was calculated from each of the 10^3 ^random samples. This yielded null distributions specific to each tissue-treatment combination and protein family, which were used to evaluate the significance of observed *T *statistics. An observed *T *statistic was significant if the proportion of random samples yielding a larger or equal *T *statistic was less than *α *= 0.05. A significant *T *statistic indicates that the expression responses among the *n *members of a protein family (with respect to a given tissue-treatment combination) are larger than expected within a random sample of *n *genes.

Hsf and Hsp expression response patterns within protein families and among individual genes were analyzed by differential expression analysis and clustering [75]. Differential expression analysis was carried out using the Limma linear modeling package available in the R Bioconductor software suite [76]. In this approach, a linear model was fit for all genes with respect to each of the 19 tissue-treatment combinations. This allowed heat shock related genes to be tested for differential expression at every time point associated with each tissue-treatment combination. For each of the 19 linear model analyses performed, P-values were adjusted for multiple comparisons using the Benjamini and Hochberg method [77,78]. The differential expression analysis was used to construct the index (*d*) introduced in the Results section.

The hierarchical ordered partitioning and collapsing hybrid (HOPACH) clustering algorithm was used to identify sub-groups of genes with similar expression response patterns in each protein family [79]. In this algorithm, the number of clusters appropriate in the final clustering solution is determined automatically according the median split silhouette criterion [80]. The HOPACH algorithm is particularly well-suited for finding homogenous clusters of small size among a limited number of genes. Stress-clusters were formed by grouping Hsf/Hsp genes with respect to their expression responses (*M*) under all 111 tissue-treatment-time combinations included in our analysis (18 tissue-treatment combinations with 6 time points + 1 tissue-treatment combination with 3 time points). The Euclidean distance metric was used to measure similarity between vectors of expression responses (*M*) associated with each Hsf/Hsp gene. To form developmental-clusters, genes were centered to have a mean expression intensity of zero across the 79 developmental series conditions, and the cosine angle similarity metric was used to cluster expression profiles of Hsf/Hsp genes within each family.

## Authors' contributions

WRS carried out numerical analyses and drafted the manuscript. MH reviewed the manuscript and made important contributions to the statistical methodology implemented in the anlaysis. APW identified the necessary database resources and critically reviewed the manuscript. All authors have read and approved this manuscript.

## Supplementary Material

Additional File 1 Differential expression, heatmaps, raw signal intensities. This file contains summary results from differential expression analyses, heatmaps of clustering solutions, and raw signal intensities of Hsf and Hsp genes (present/absent calls). Click here for file

Additional File 2 Expression response profiles of individual Hsf and Hsp genes. This file contains expression response profiles for each individual Hsf and Hsp gene under all stress conditions examined in root, shoot, and leaf tissue. Click here for file

## Figures and Tables

**Table 1:** Overview: Responsiveness of heat shock gene families to abiotic and biotic stress treatments.

Tissue-Treatment	Hsf (*n *= 21)	Hsp20 (*n *= 18)	Hsp70 (*n *= 13)	Hsp90 (*n *= 6)	Hsp100 (*n *= 7)
Roots					
cold	0.52(0.001)	1.24(< 0.001)	0.45(0.026)	0.32(0.364)	0.25(0.660)
osmotic	0.75(< 0.001)	2.12(< 0.001)	0.48(0.089)	0.74(0.025)	0.66(0.040)
salt	1.17(< 0.001)	1.70(< 0.001)	0.45(0.341)	0.64(0.133)	0.52(0.269)
drought	0.33(0.001)	0.56(< 0.001)	0.26(0.087)	0.15(0.824)	0.20(0.432)
genotoxic	0.24(0.246)	0.72(< 0.001)	0.31(0.061)	0.27(0.258)	0.27(0.243)
oxidative	0.23(0.035)	0.61(< 0.001)	0.26(0.021)	0.19(0.417)	0.31(0.021)
UV-B	0.27(0.050)	0.96(< 0.001)	0.27(0.107)	0.17(0.797)	0.24(0.288)
wounding	0.27(0.043)	1.18(< 0.001)	0.35(0.007)	0.32(0.069)	0.35(0.030)
heat	0.49(0.003)	4.32(< 0.001)	1.55(< 0.001)	1.02(< 0.001)	0.50(0.048)
Shoots					
cold	0.62(0.001)	0.51(0.023)	0.44(0.136)	0.57(0.070)	0.35(0.423)
osmotic	0.83(< 0.001)	1.35(< 0.001)	0.76(0.008)	0.46(0.276)	0.65(0.078)
salt	0.52(0.001)	1.00(< 0.001)	0.44(0.017)	0.39(0.116)	0.31(0.275)
drought	0.36(0.002)	0.66(< 0.001)	0.31(0.057)	0.28(0.198)	0.28(0.192)
genotoxic	0.22(0.343)	0.35(0.001)	0.20(0.607)	0.23(0.374)	0.23(0.363)
oxidative	0.29(0.005)	0.78(< 0.001)	0.48(< 0.001)	0.22(0.344)	0.33(0.016)
UV-B	0.50(0.011)	0.69(< 0.001)	0.53(0.024)	0.69(0.020)	0.32(0.499)
wounding	0.33(0.018)	0.62(< 0.001)	0.27(0.225)	0.35(0.099)	0.28(0.248)
heat	0.55(< 0.001)	4.66(< 0.001)	1.34(< 0.001)	1.45(< 0.001)	0.32(0.339)
Leaves					
pathogen	0.24(0.701)	0.40(0.096)	0.78(0.001)	0.75(0.018)	0.46(0.134)

The table lists values of the *T *statistic associated with each tissue-treatment combination with respect to each of five protein families (Hsf, Hsp20, Hsp70, Hsp90, Hsp100). The value of *T *is proportional to the median level of log_2 _fold-change induced by a given stress treatment among the *n *gene members within a protein family (see Equation 2 in Methods). The P-values associated with each statistic are shown in parentheses. P-values were obtained by genome-wide resampling and represent the probability of obtaining an equal or larger value of *T *based on 10,000 random samples of *n *genes from the *N *= 22746 genes represented on the ATH1 array. P-values exceeding 0.0245 in the table below are non-significant following the Benjamini-Hochberg adjustment for multiple testing (with nominal type I error rate of *α *= 0.05).

**Table 2:** Members of the heat shock transcription factor protein family

Gene Name	Cluster ID (Development)	Cluster ID (Stress)	*d*
HsfA9	31	452	0.018
HsfA5	50	452	0.061
HsfA1a	32	452	0.070
HsfA7b	60	440	0.088
HsfA7a	80	300	0.123
HsfA6a	31	420	0.149
HsfA1b	70	452	0.167
HsfB3	40	430	0.184
HsfA4c	40	452	0.193
HsfA1d	10	451	0.202
HsfA2	60	200	0.228
HsfB4	90	451	0.228
HsfA1e	60	460	0.237
HsfA3	20	410	0.272
HsfA6b	31	100	0.307
HsfB2b*	60	470	0.316
HsfA4a	20	490	0.351
HsfA8	20	480	0.395
HsfB2a*	60	470	0.439
HsfB1	40	500	0.447
HsfC1	80	600	0.456

Genes are ordered from least to most stress-responsive (according to *d*). The value of *d *represents the mean proportion of time points, among the 19 tissue-treatment combinations considered, at which a gene was differentially expressed. Cluster IDs represent gene groupings determined by the HOPACH clustering algorithm (see Methods). The development cluster analysis was carried out with respect to the developmental series conditions of Schmid et al. (2005) [41]. The stress cluster analysis was carried out with respect to expression responses observed under each of the 111 tissue-treatment-time combinations examined in this study. The *j*th digit in each cluster ID indicates the group to which a gene was assigned in the *j*th iteration of the HOPACH algorithm (see [76]). Heat maps corresponding to development and stress clustering solutions are provided in section 1C of additional file 1.

(*)Genes clustered together with respect to both Development and Stress datasets

**Table 3:** Members of the Hsp20 protein family

Gene Name	Cluster ID (Development)	Cluster ID (Stress)	*d*
AtHsp14.2-P(r)	50	60	0.096
AtHsp25.4-P	24	30	0.167
AtHsp17.6-CII	25	21	0.184
AtHsp23.6-M	22	30	0.184
AtHsp22.0-ER	22	42	0.193
AtHsp23.5-M	30	10	0.193
AtHsp17.6B-Cl	28	21	0.202
AtHsp17.7-CII*	27	21	0.219
AtHsp21.7-Cl(r)	70	44	0.219
AtHsp26.5-P(r)	26	42	0.219
AtHsp17.6A-Cl	23	22	0.228
AtHsp18.1-Cl	40	41	0.237
AtHsp17.6C-Cl*	27	21	0.254
AtHsp17.4-Cl*	27	21	0.263
AtHsp15.7-Cl(r)	21	42	0.263
AtHsp17.4-CIII	10	43	0.307
AtHsp15.4-Cl(r)	60	70	0.316
AtHsp18.5-Cl(r)	70	50	0.325

Genes are ordered from least to most stress-responsive (according to *d*). See Table 2 caption for an explanation of clustering procedures and the value of *d*. Heat maps corresponding to clustering solutions are provided in section 2C of additional file 1.

(*)Genes clustered together with respect to both Development and Stress datasets

**Table 4:** Members of the Hsp70 protein family

Gene Name	Cluster ID (Development)	Cluster ID (Stress)	*d*
AtHsp70-15*	5	54	0.202
AtHsp70-5	2	80	0.211
AtHsp70-8	2	70	0.219
AtHsp70-6	7	53	0.228
AtHsp70-17	6	54	0.228
AtHsp70-9	5	52	0.254
AtHsp70-1*	5	54	0.272
AtHsp70-10	4	40	0.281
AtHsp70-11	1	60	0.333
AtHsp70-7	7	51	0.351
AtHsp70-3	4	20	0.368
AtHsp70-2	3	30	0.386
AtHsp70-4	1	21	0.439

Genes are ordered from least to most stress-responsive (according to *d*). See Table 2 caption for an explanation of clustering procedures and the value of *d*. Heat maps corresponding to clustering solutions are provided in section 3C of additional file 1.

(*)Genes clustered together with respect to both Development and Stress datasets

**Table 5:** Members of the Hsp90 protein family

Gene Name	Cluster ID (Development)	Cluster ID (Stress)	*d*
AtHsp90-6*	3	2	0.281
AtHsp90-4*	3	2	0.298
AtHsp90-5	2	2	0.307
AtHsp90-7	4	2	0.316
AtHsp90-1	1	1	0.333
AtHsp90-2*	3	2	0.342

Genes are ordered from least to most stress-responsive (according to *d*). See Table 2 caption for an explanation of clustering procedures and the value of *d*. Heat maps corresponding to clustering solutions are provided in section 4C of additional file 1.

(*) Genes clustered together with respect to both Development and Stress datasets

**Table 6:** Members of the Hsp100 protein family

Gene Name	Cluster ID (Development)	Cluster ID (Stress)	*d*
AtHsp100-2	2	3	0.009
AtHsp100-5	3	3	0.140
AtHsp100-1	1	1	0.228
AtHsp100-8	3	5	0.246
AtHsp100-4	1	2	0.254
AtHsp100-3	4	3	0.316
AtHsp100-7	3	4	0.368

Genes are ordered from least to most stress-responsive (according to *d*). See Table 2 caption for an explanation of clustering procedures and the value of *d*. Heat maps corresponding to clustering solutions are provided in section 5C of additional file 1.
