# Supplementary material for: Transcriptional profiling of Arabidopsis heat shock proteins and transcription factors reveals extensive overlap between heat and non-heat stress response pathways
Source: BMC Genomics. 2007 May 22;8:125. doi: 10.1186/1471-2164-8-125 (PMC1887538; doi:10.1186/1471-2164-8-125)
Supplement: Additional File 2 — Expression response profiles of individual Hsf and Hsp genes. This file contains expression response profiles for each individual Hsf and Hsp gene under all stress conditions examined in root, shoot, and leaf tissue. [file 1471-2164-8-125-S2.pdf]

Additional File 2:

Transcriptional profiling of Arabidopsis heat shock proteins and transcription factors reveals extensive overlap between heat and non−heat stress response pathways

William R. Swindell(1), Marianne Huebner(1), Andreas P. Weber(2)
(1) Michigan State University, Dept. of Statistics and Probability
(2) Michigan State University, Dept. of Plant Biology

This file displays stress−response profile plots for each individual heat shock protein and transcription factor analyzed in this study. Expression response profiles are shown for all abiotic stress treatments applied to roots and shoots, and for the pathogen treatment applied to leaves. The horizontal axis of each plot corresponds to time (hours), while the vertical axis corresponds to the log2 fold−change associated with a given stress treatment.

Contact: William R. Swindell, swindel5@msu.edu

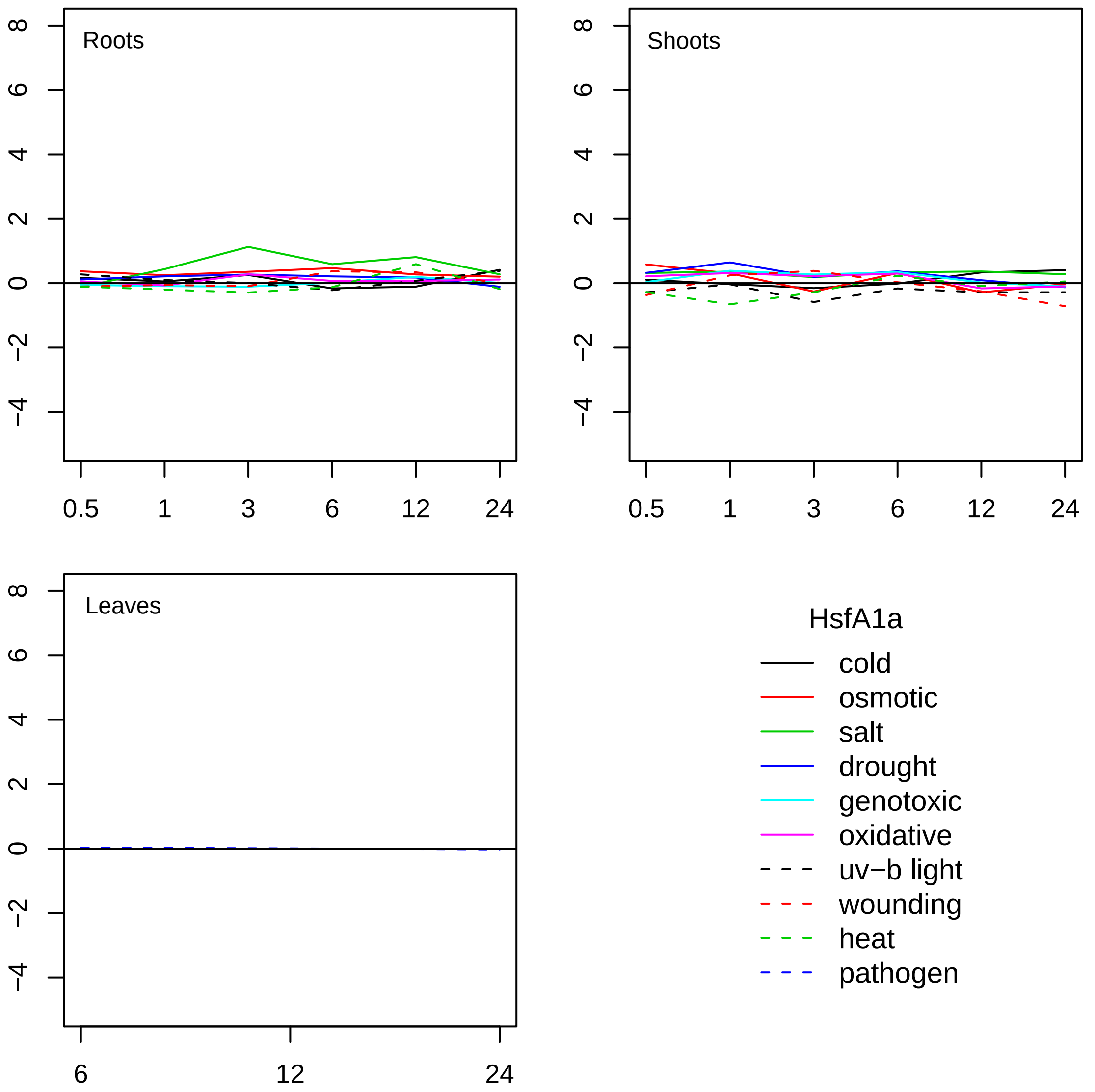

Roots
Shoots
Leaves
8
6
4
2
0
−2
−4
0.5
1
3
6
12
24
HsfA1a
cold
osmotic
salt
drought
genotoxic
oxidative
uv−b light
wounding
heat
pathogen

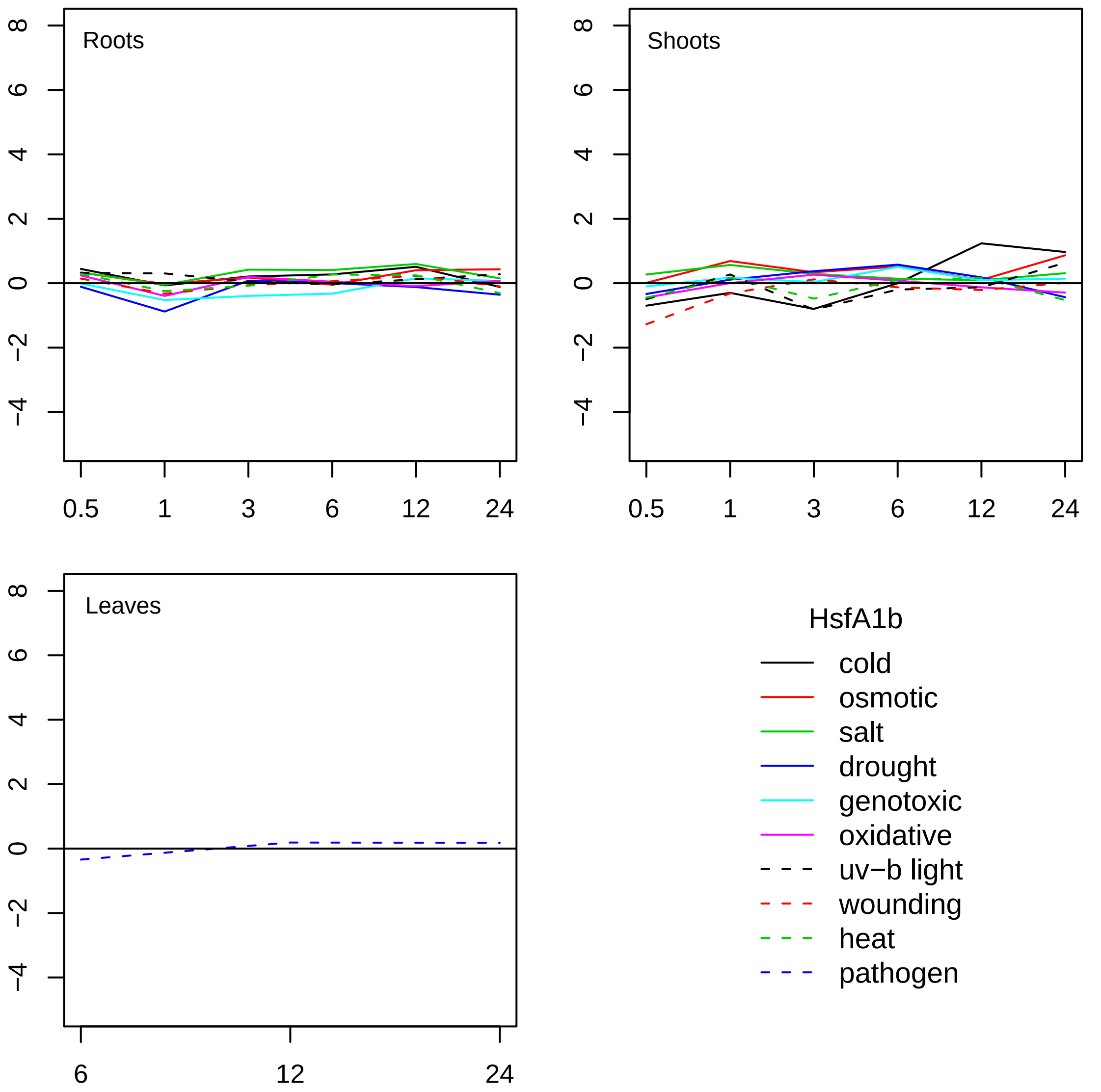
Roots
Shoots
Leaves
HsfA1b
cold
osmotic
salt
drought
genotoxic
oxidative
uv−b light
wounding
heat
pathogen
0.5
1
3
6
12
24
8
6
4
2
0
−2
−4

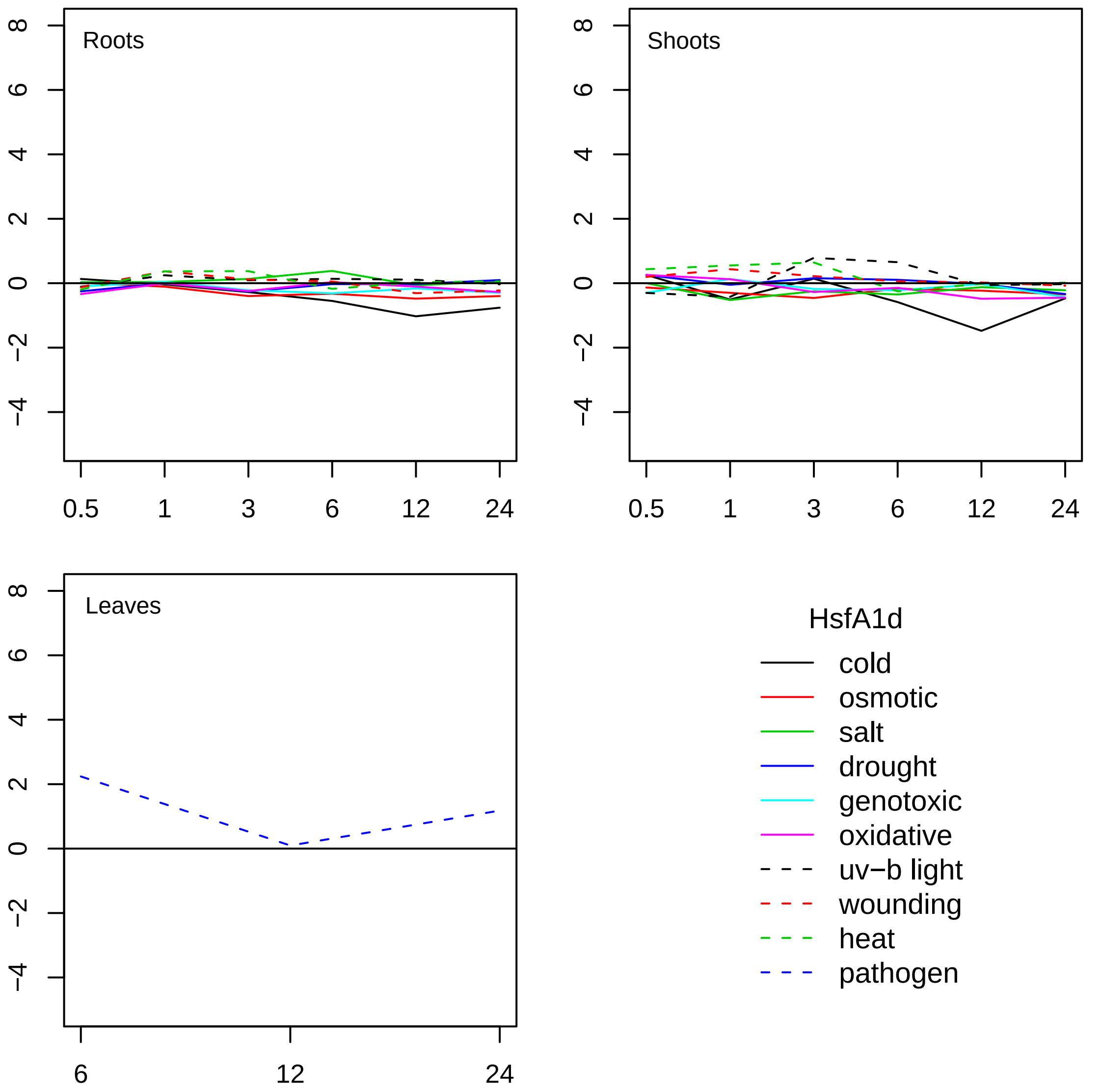
Roots
Shoots
Leaves
8
6
4
2
0
−2
−4
0.5
1
3
6
12
24
HsfA1d
cold
osmotic
salt
drought
genotoxic
oxidative
uv−b light
wounding
heat
pathogen

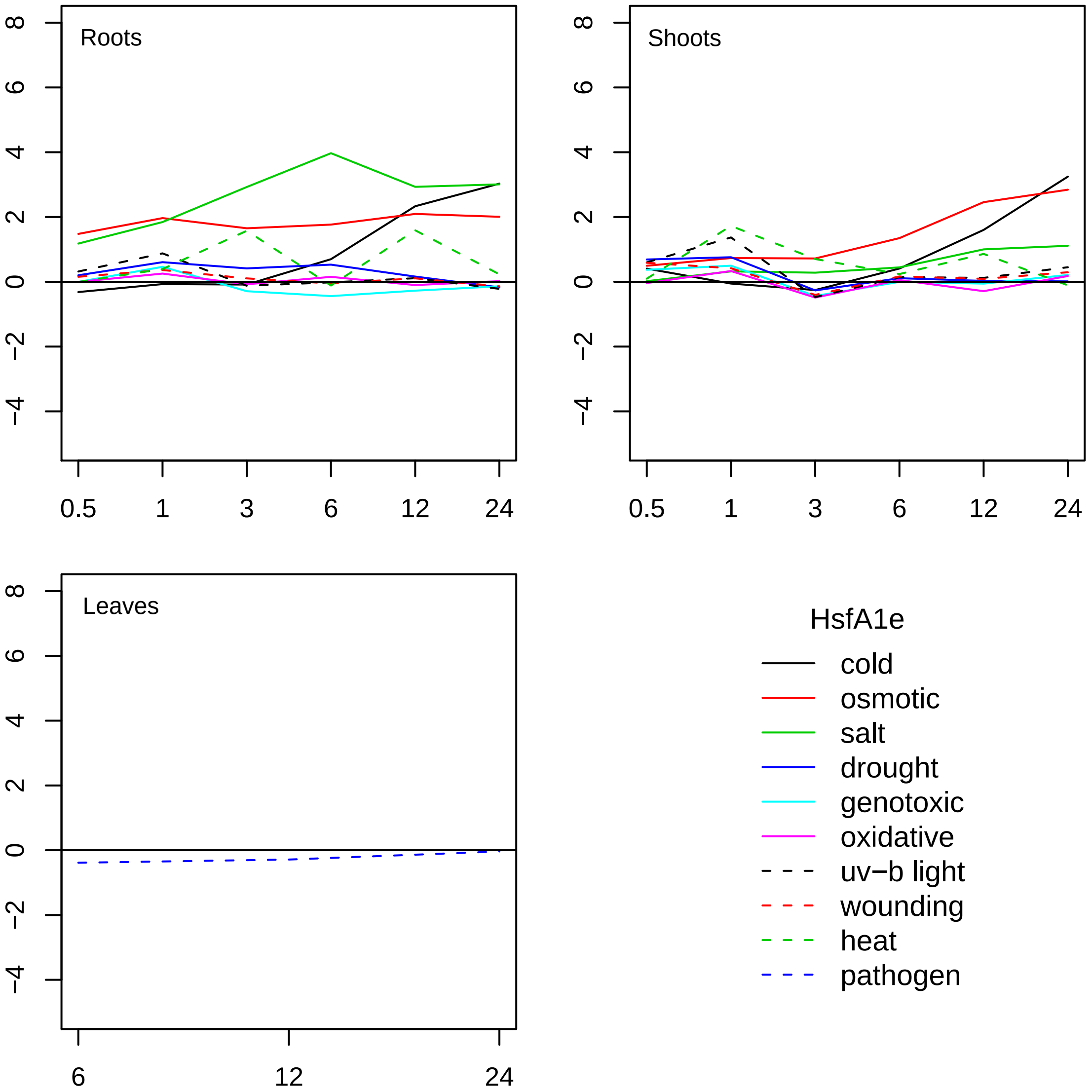

Roots
Shoots
Leaves
8
6
4
2
0
−2
−4
0.5
1
3
6
12
24
HsfA1e
cold
osmotic
salt
drought
genotoxic
oxidative
uv−b light
wounding
heat
pathogen

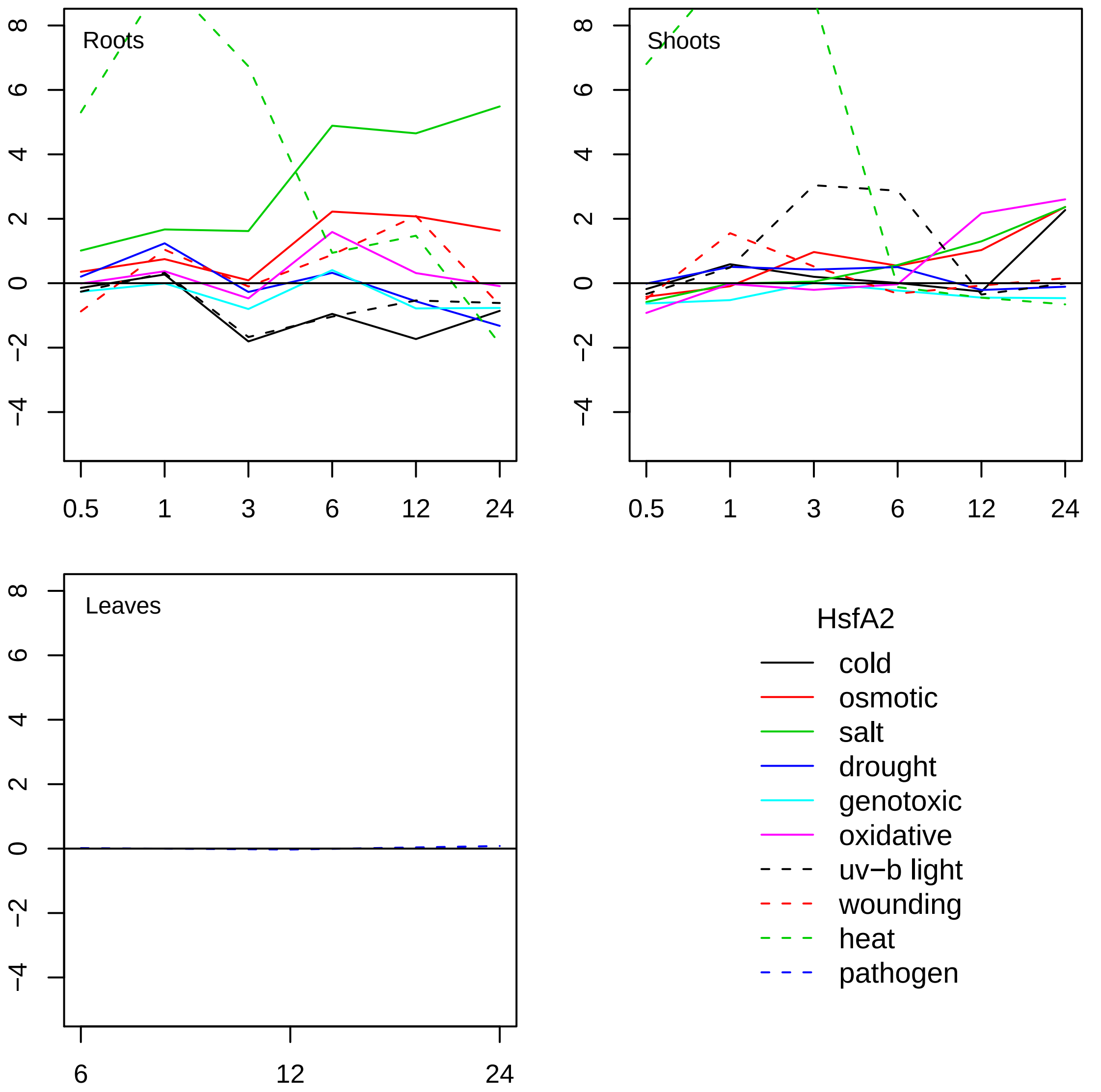

Roots
Shoots
Leaves
0.5
1
3
6
12
24
HsfA2
cold
osmotic
salt
drought
genotoxic
oxidative
uv−b light
wounding
heat
pathogen

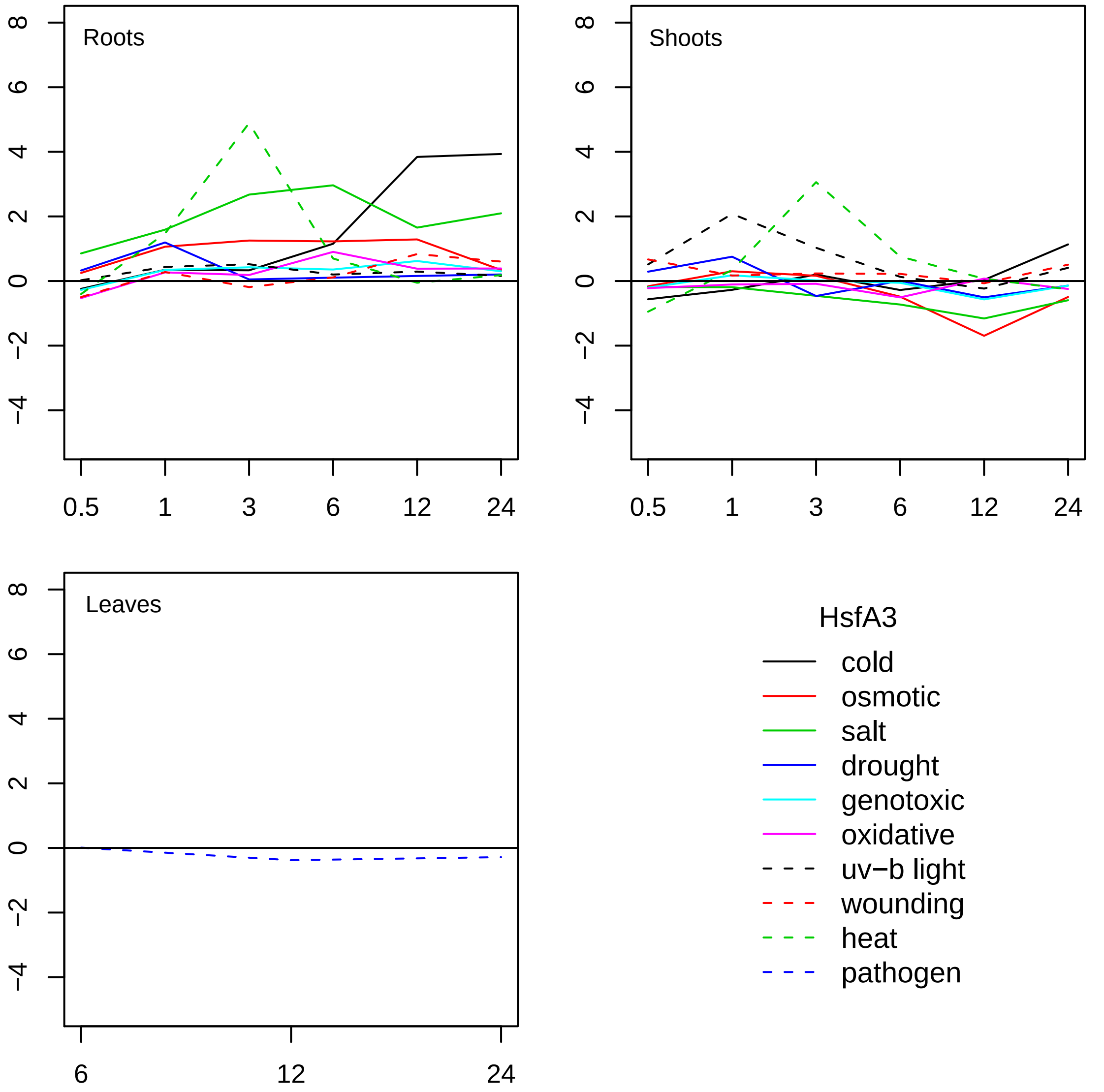
Roots
Shoots
Leaves
8
6
4
2
0
−2
−4
0.5
1
3
6
12
24
HsfA3
cold
osmotic
salt
drought
genotoxic
oxidative
uv−b light
wounding
heat
pathogen

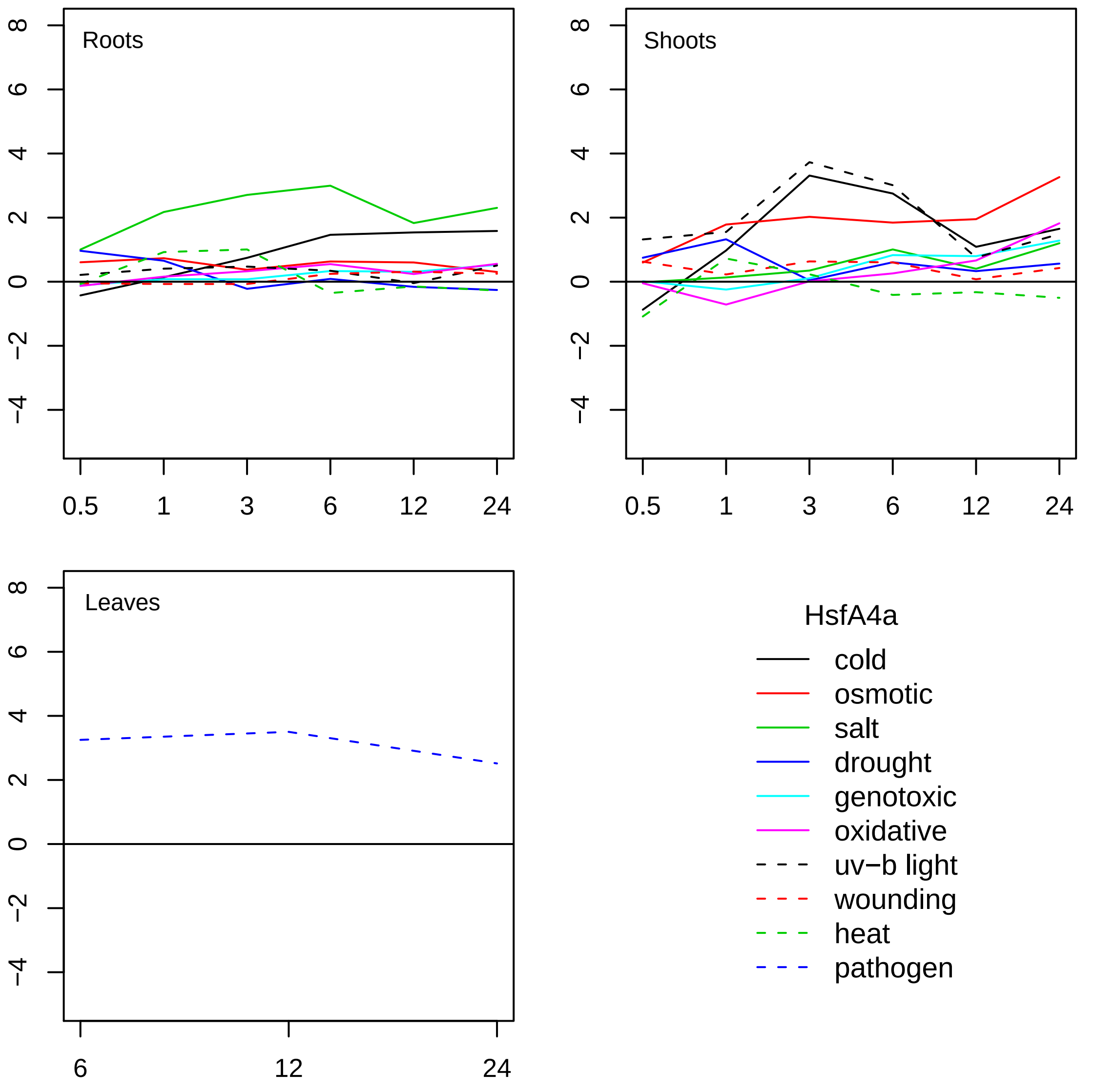

Roots
Shoots
Leaves
8
6
4
2
0
−2
−4
0.5
1
3
6
12
24
HsfA4a
cold
osmotic
salt
drought
genotoxic
oxidative
uv−b light
wounding
heat
pathogen

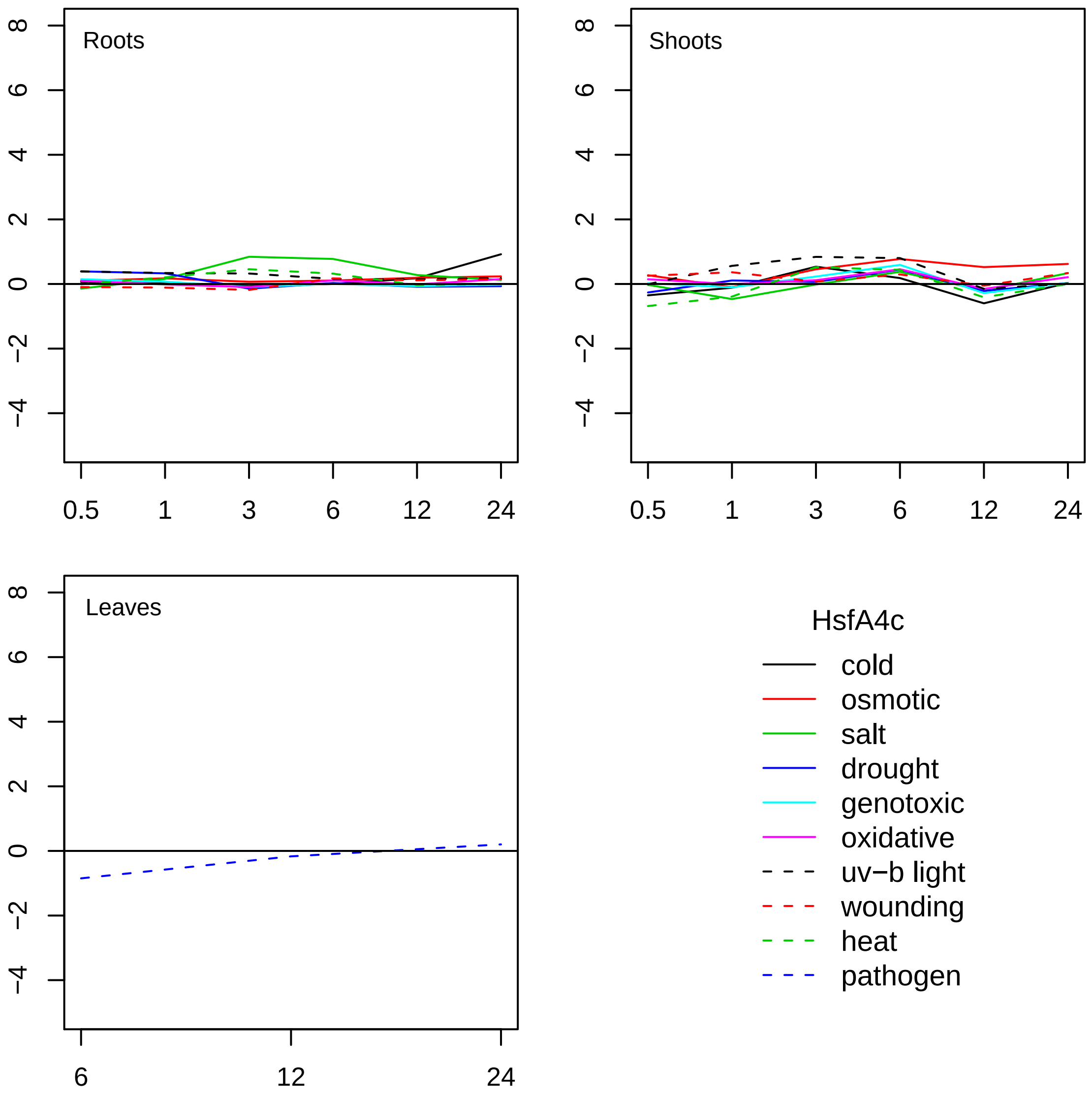

Roots
Shoots
Leaves
8
6
4
2
0
−2
−4
0.5
1
3
6
12
24
HsfA4c
cold
osmotic
salt
drought
genotoxic
oxidative
uv−b light
wounding
heat
pathogen

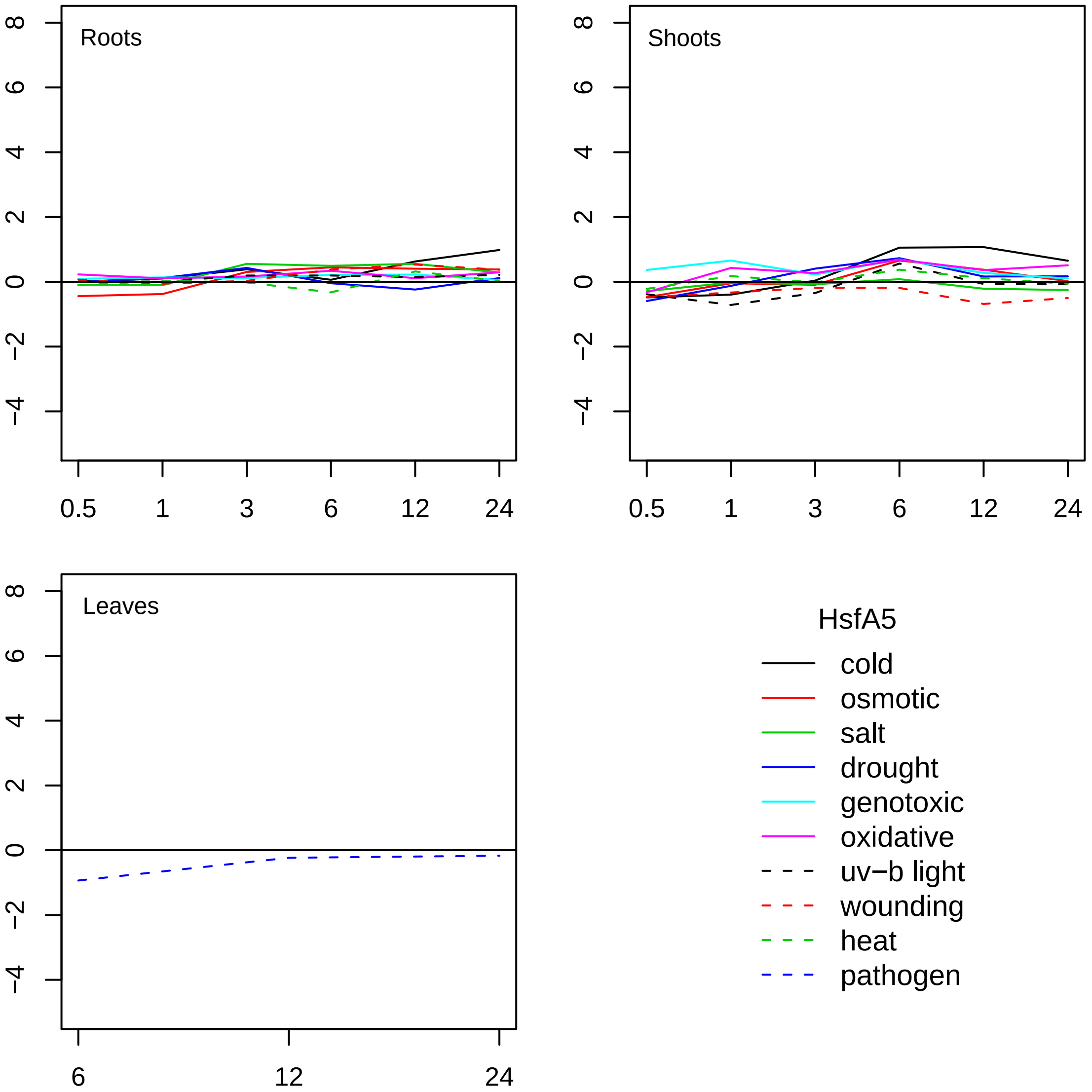

Roots
Shoots
Leaves
8
6
4
2
0
−2
−4
0.5
1
3
6
12
24
HsfA5
cold
osmotic
salt
drought
genotoxic
oxidative
uv−b light
wounding
heat
pathogen

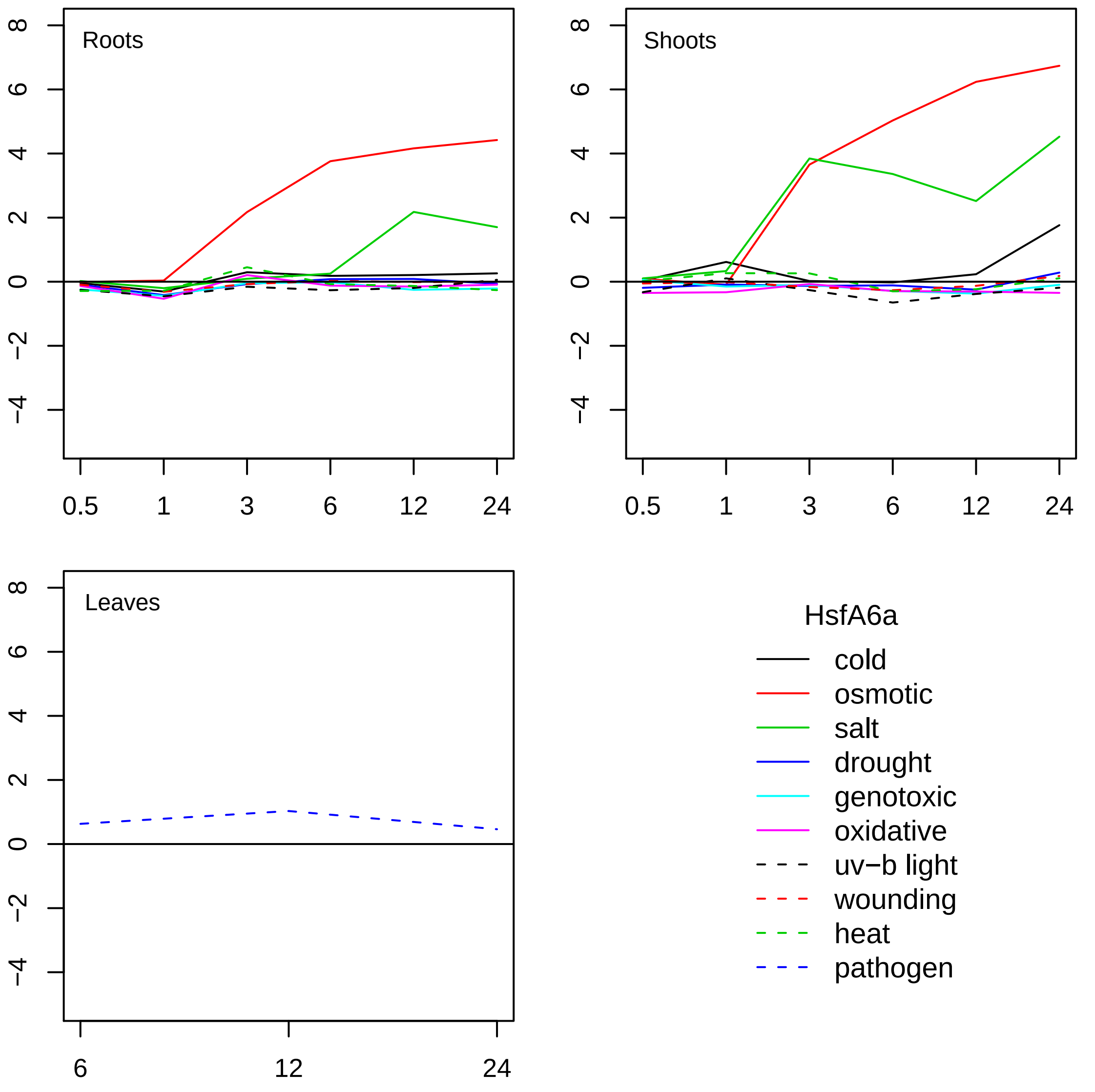

Roots
Shoots
Leaves
8
6
4
2
0
−2
−4
0.5
1
3
6
12
24
HsfA6a
cold
osmotic
salt
drought
genotoxic
oxidative
uv−b light
wounding
heat
pathogen

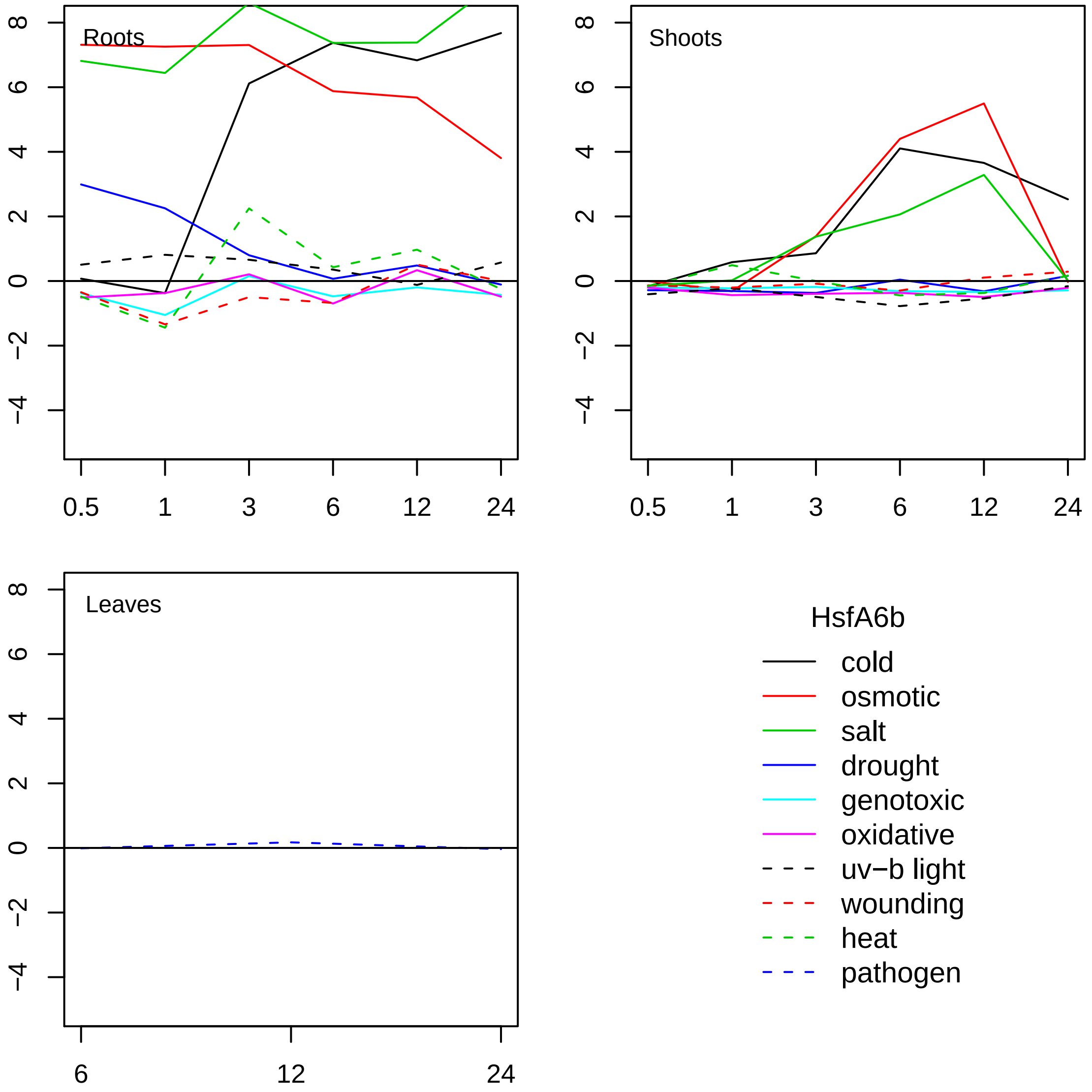
Roots
Shoots
Leaves
8
6
4
2
0
−2
−4
0.5
1
3
6
12
24
HsfA6b
cold
osmotic
salt
drought
genotoxic
oxidative
uv−b light
wounding
heat
pathogen

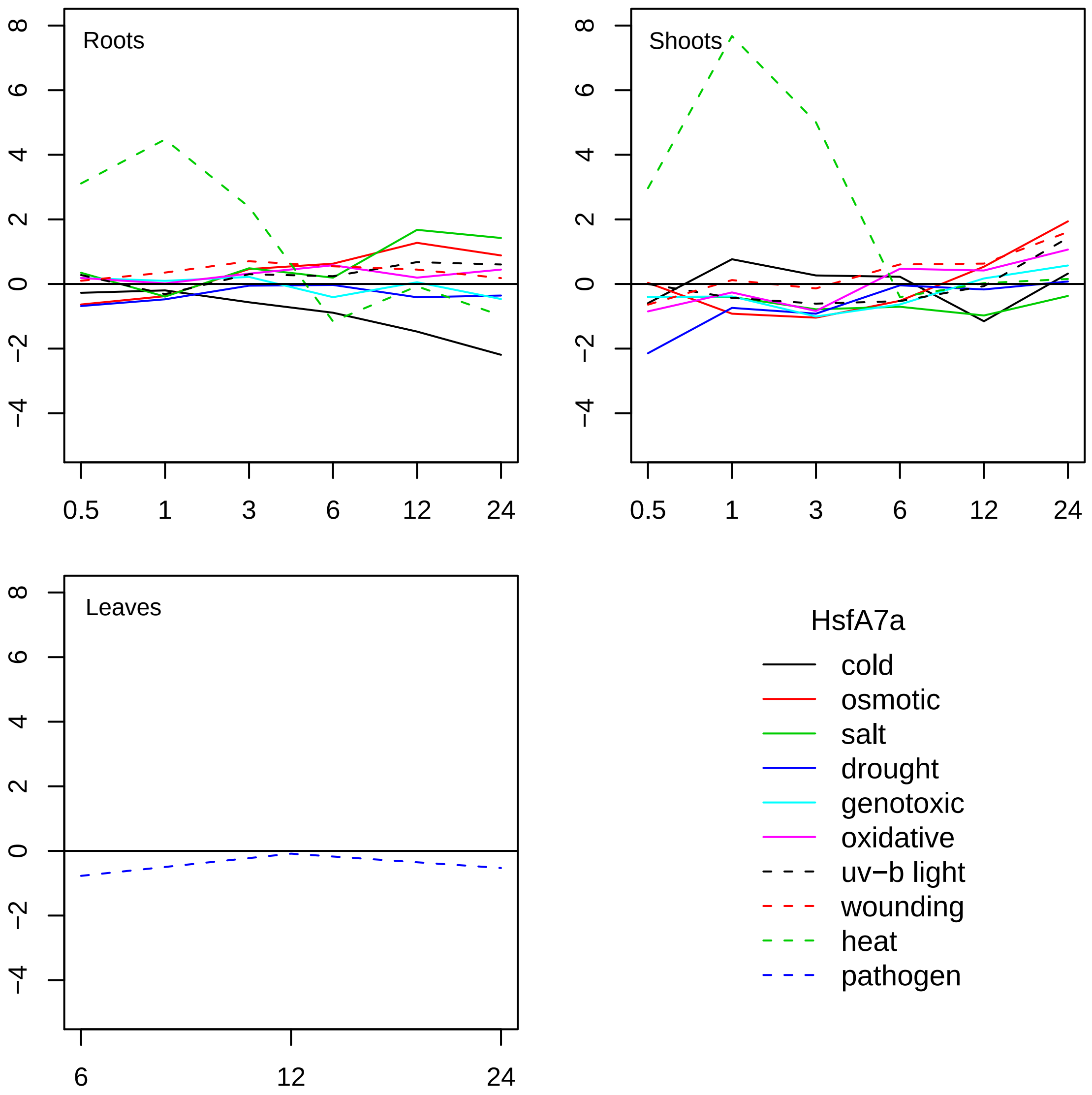

Roots
Shoots
Leaves
8
6
4
2
0
−2
−4
0.5
1
3
6
12
24
HsfA7a
cold
osmotic
salt
drought
genotoxic
oxidative
uv−b light
wounding
heat
pathogen

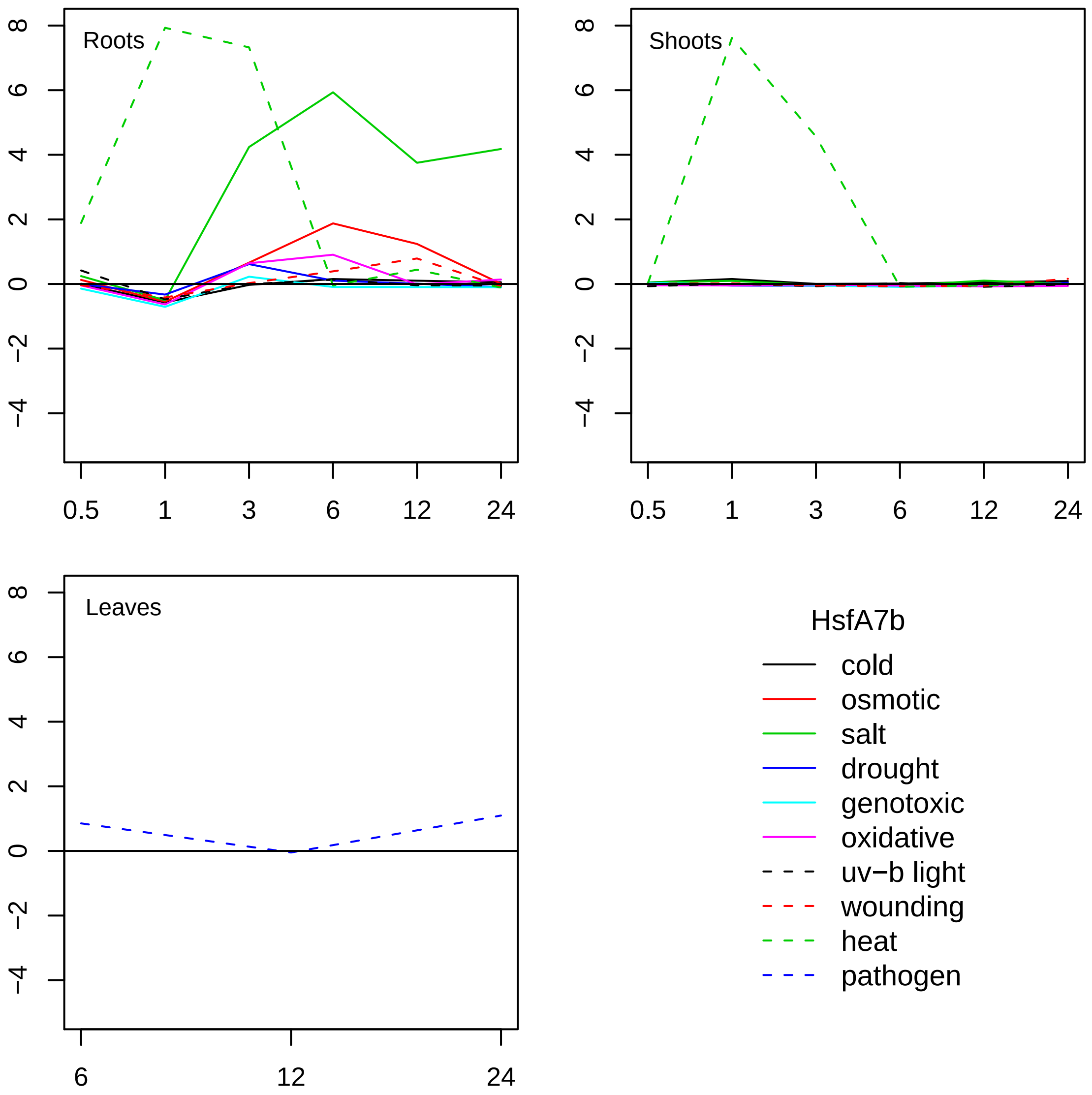
Roots
Shoots
Leaves
8
6
4
2
0
−2
−4
0.5
1
3
6
12
24
HsfA7b
cold
osmotic
salt
drought
genotoxic
oxidative
uv−b light
wounding
heat
pathogen

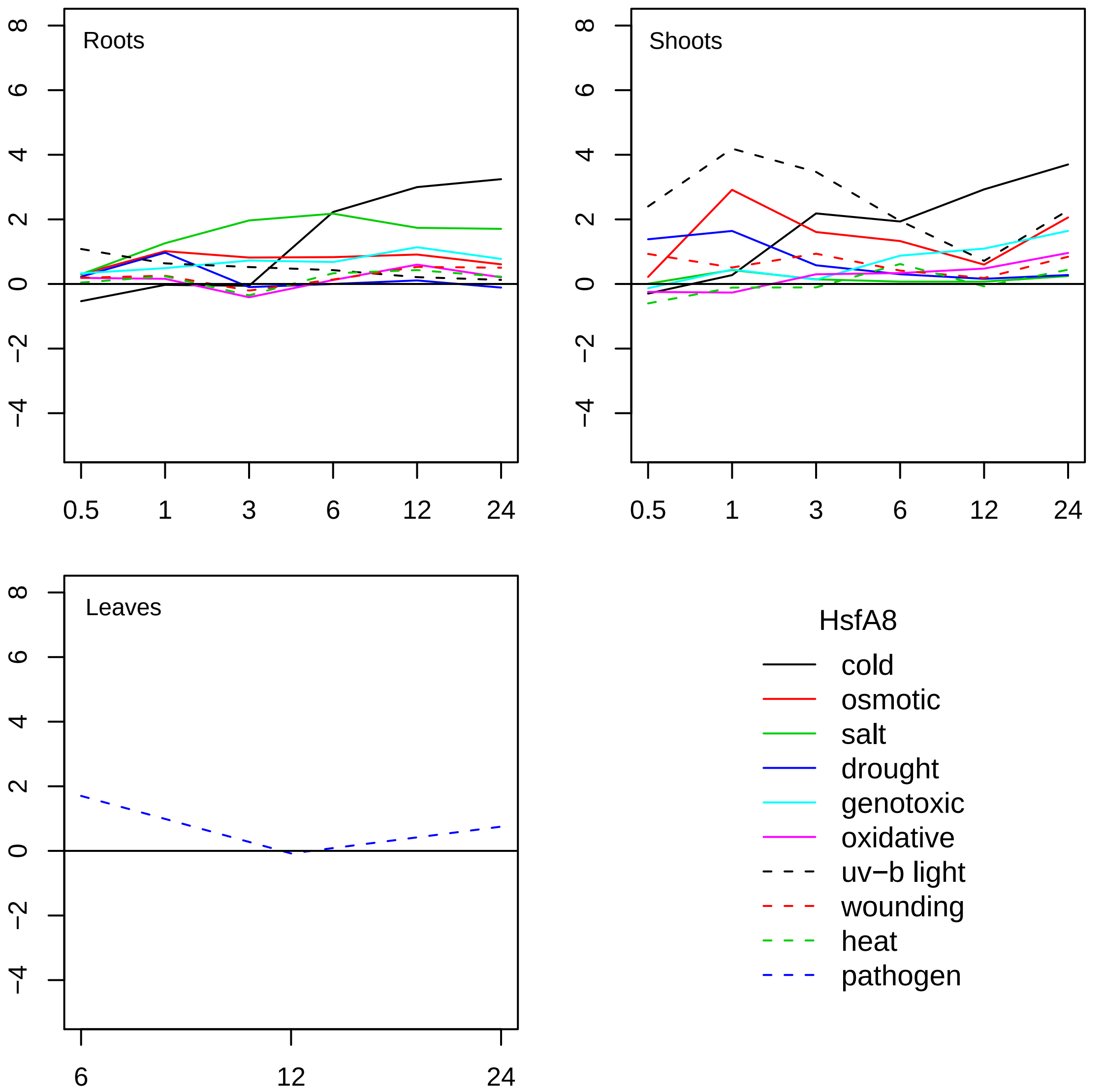

Roots
Shoots
Leaves
8
6
4
2
0
−2
−4
0.5
1
3
6
12
24
HsfA8
cold
osmotic
salt
drought
genotoxic
oxidative
uv−b light
wounding
heat
pathogen

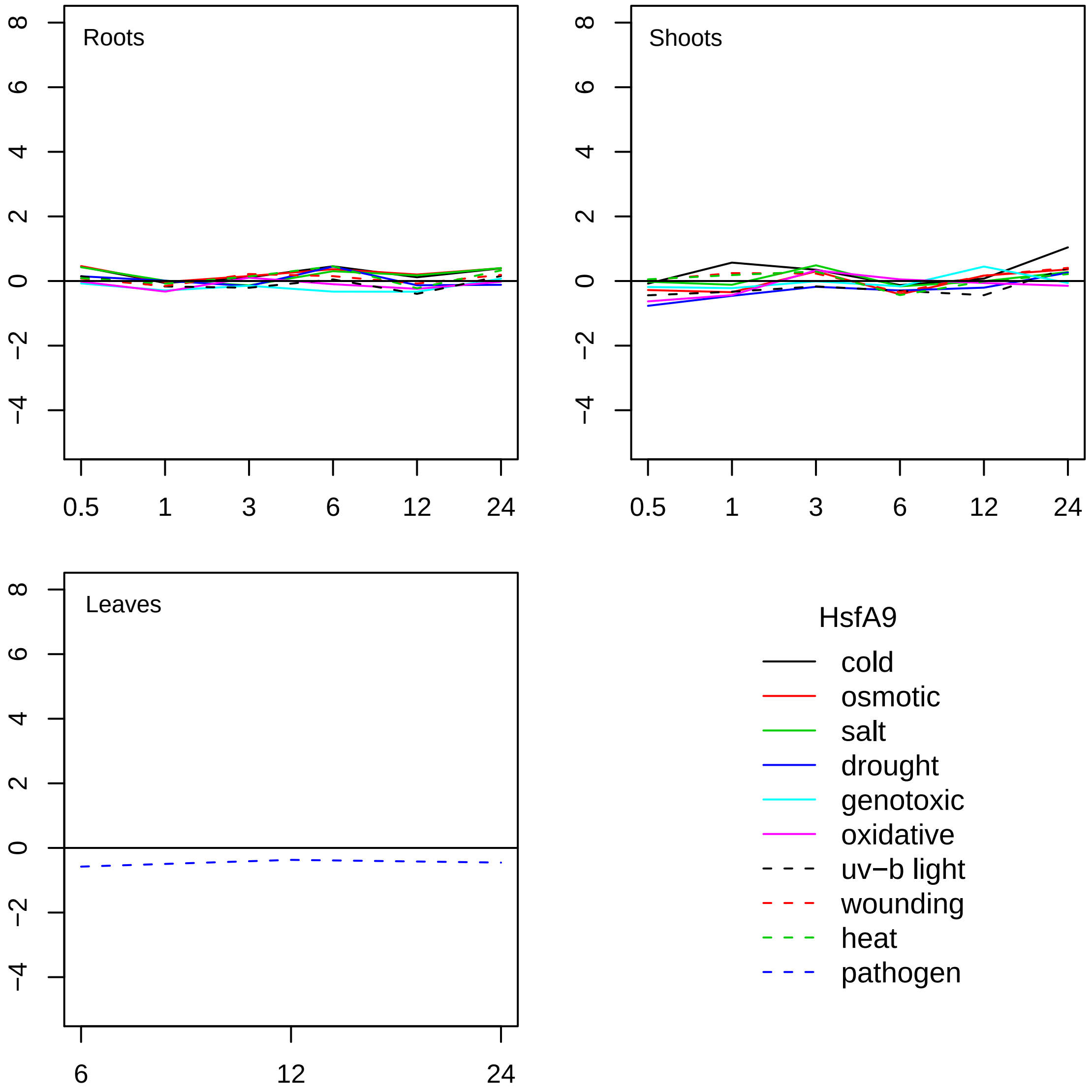
Roots
Shoots
Leaves
0.5
1
3
6
12
24
8
6
4
2
0
−2
−4
HsfA9
cold
osmotic
salt
drought
genotoxic
oxidative
uv−b light
wounding
heat
pathogen

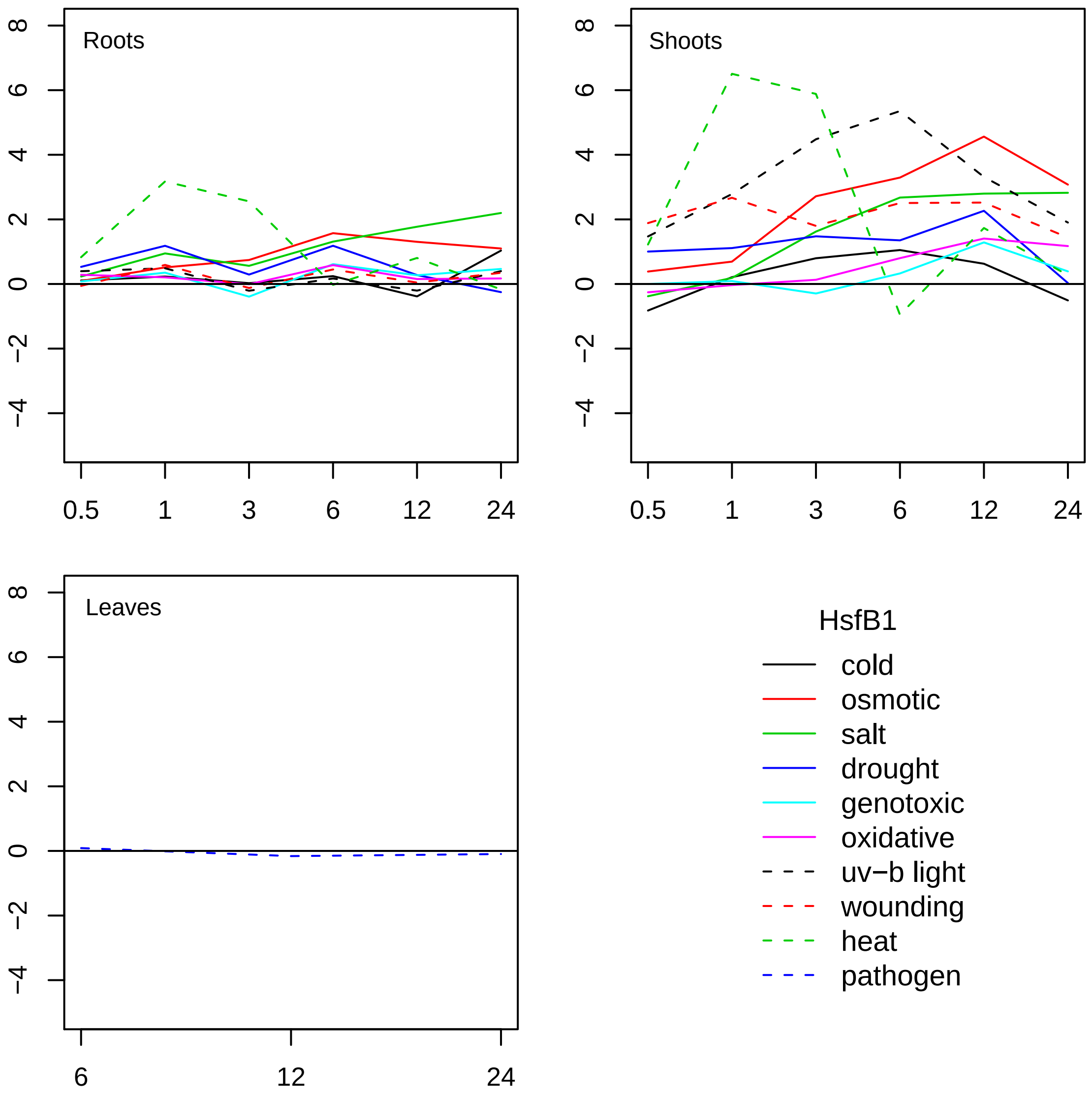
Roots
Shoots
Leaves
8
6
4
2
0
−2
−4
0.5
1
3
6
12
24
HsfB1
cold
osmotic
salt
drought
genotoxic
oxidative
uv−b light
wounding
heat
pathogen

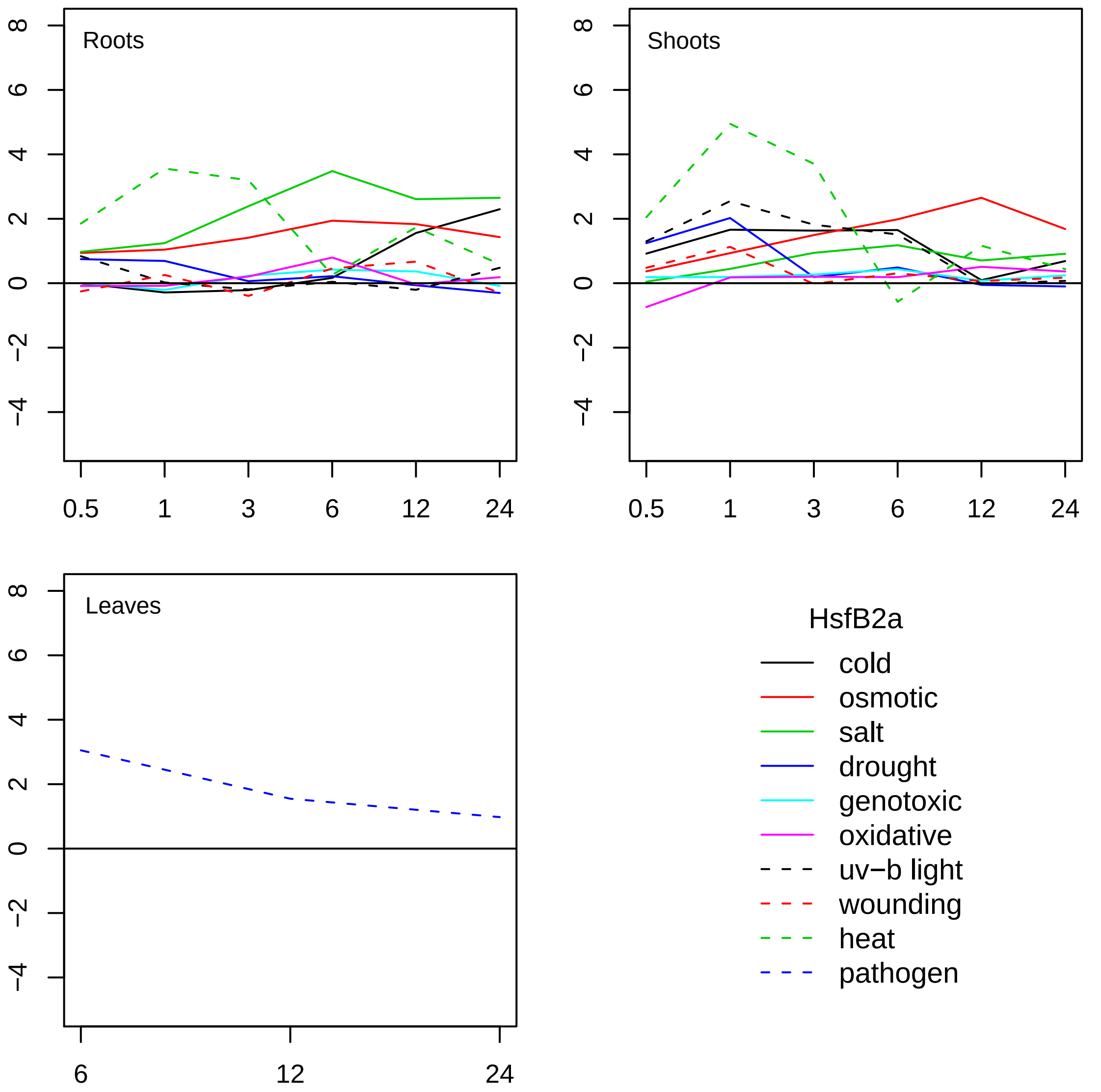

Roots
Shoots
Leaves
HsfB2a
cold
osmotic
salt
drought
genotoxic
oxidative
uv−b light
wounding
heat
pathogen
0.5
1
3
6
12
24
8
6
4
2
0
−2
−4

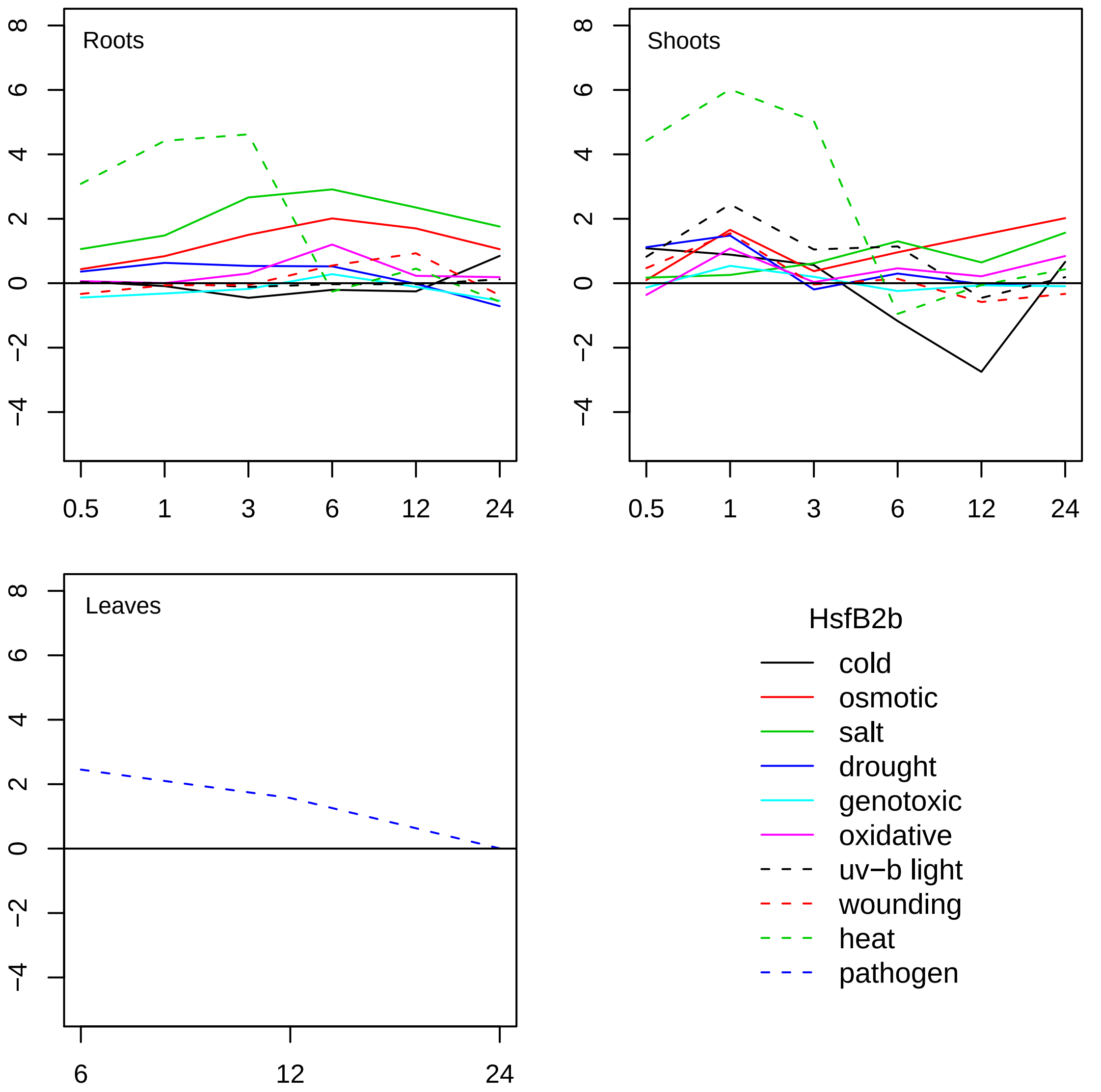

Roots
Shoots
Leaves
8
6
4
2
0
−2
−4
0.5
1
3
6
12
24
HsfB2b
cold
osmotic
salt
drought
genotoxic
oxidative
uv−b light
wounding
heat
pathogen

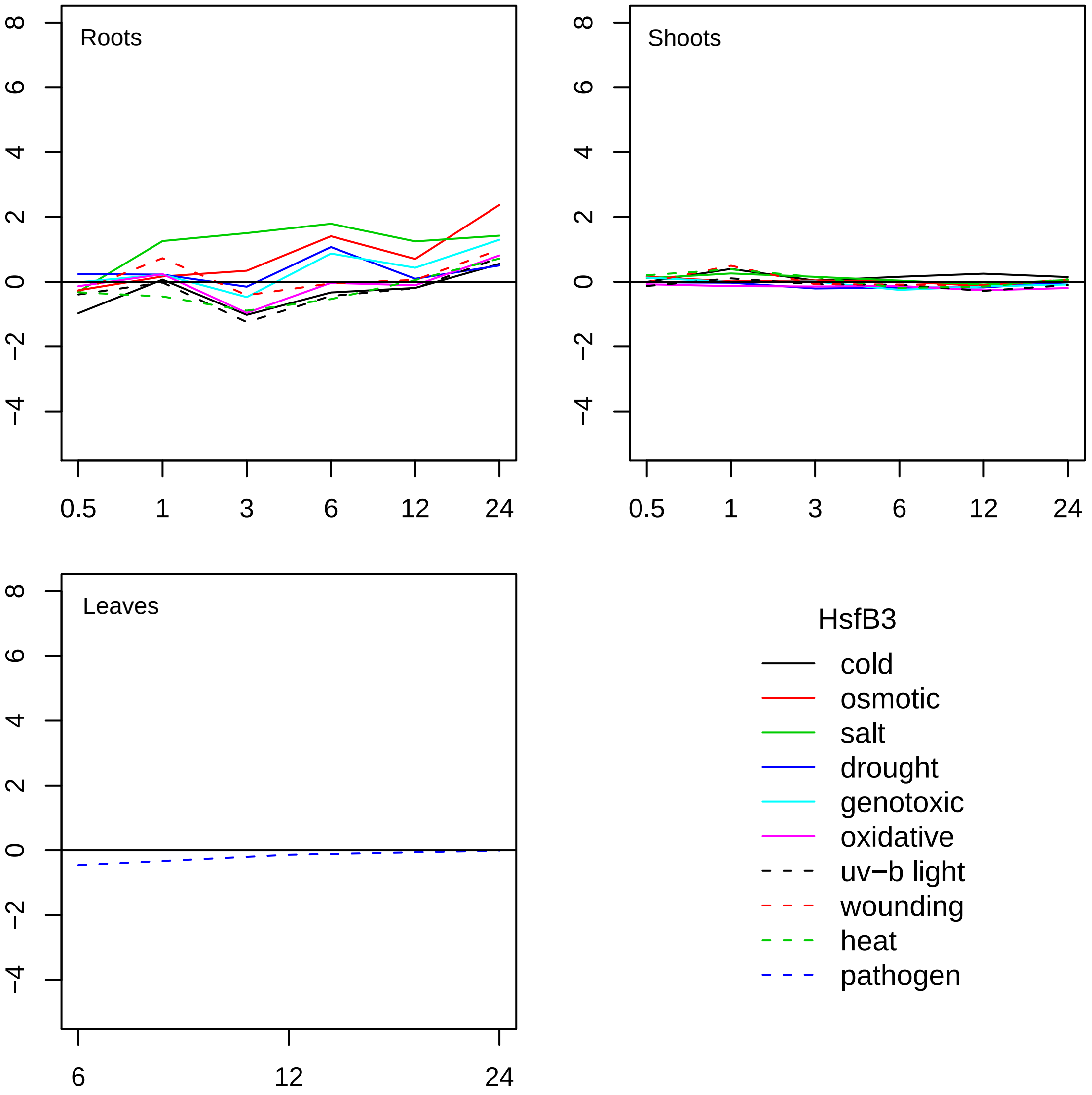

Roots
Shoots
Leaves
8
6
4
2
0
−2
−4
0.5
1
3
6
12
24
HsfB3
cold
osmotic
salt
drought
genotoxic
oxidative
uv−b light
wounding
heat
pathogen

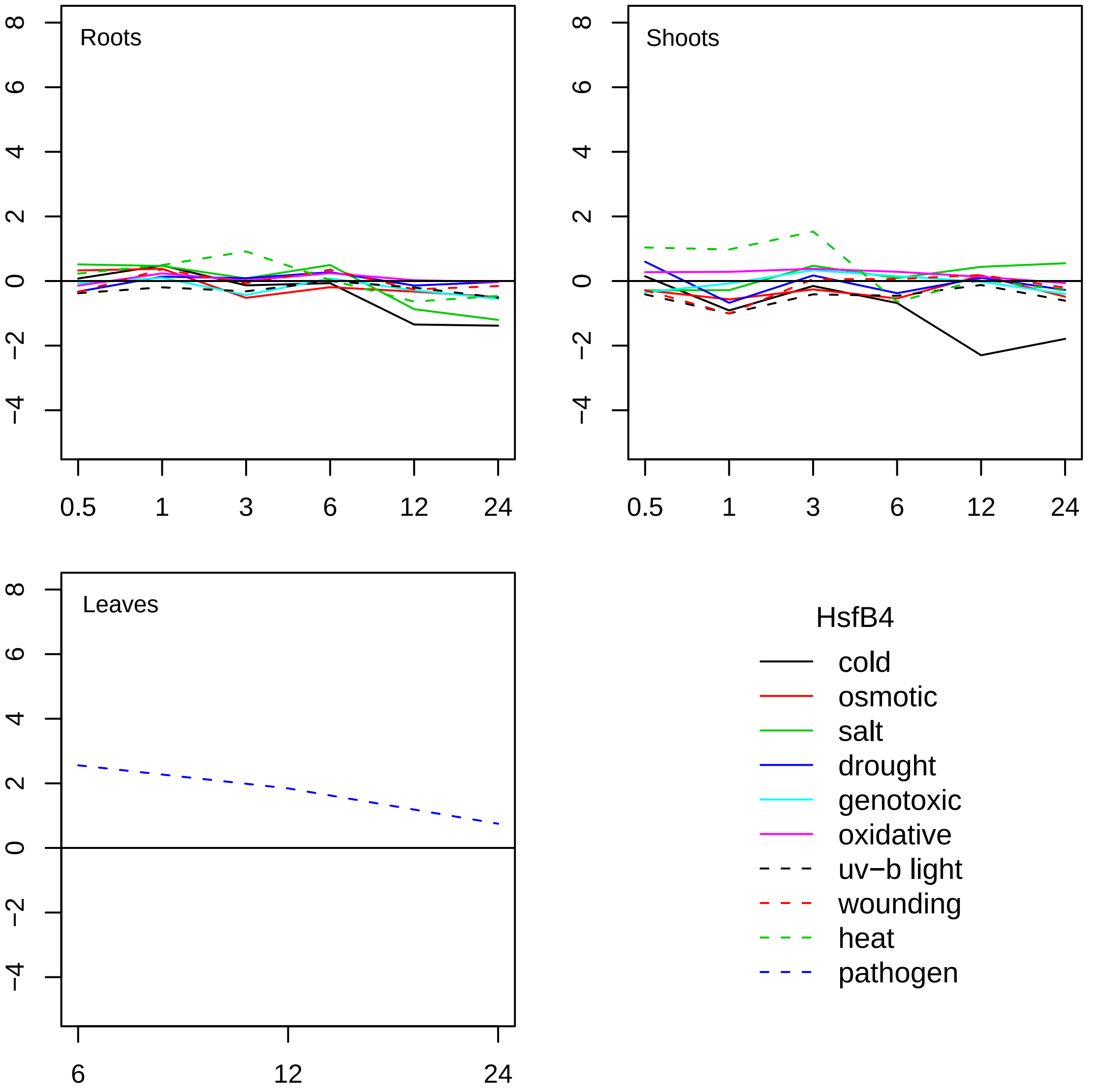
Roots
Shoots
Leaves
HsfB4
cold
osmotic
salt
drought
genotoxic
oxidative
uv−b light
wounding
heat
pathogen
0.5
1
3
6
12
24
8
6
4
2
0
−2
−4

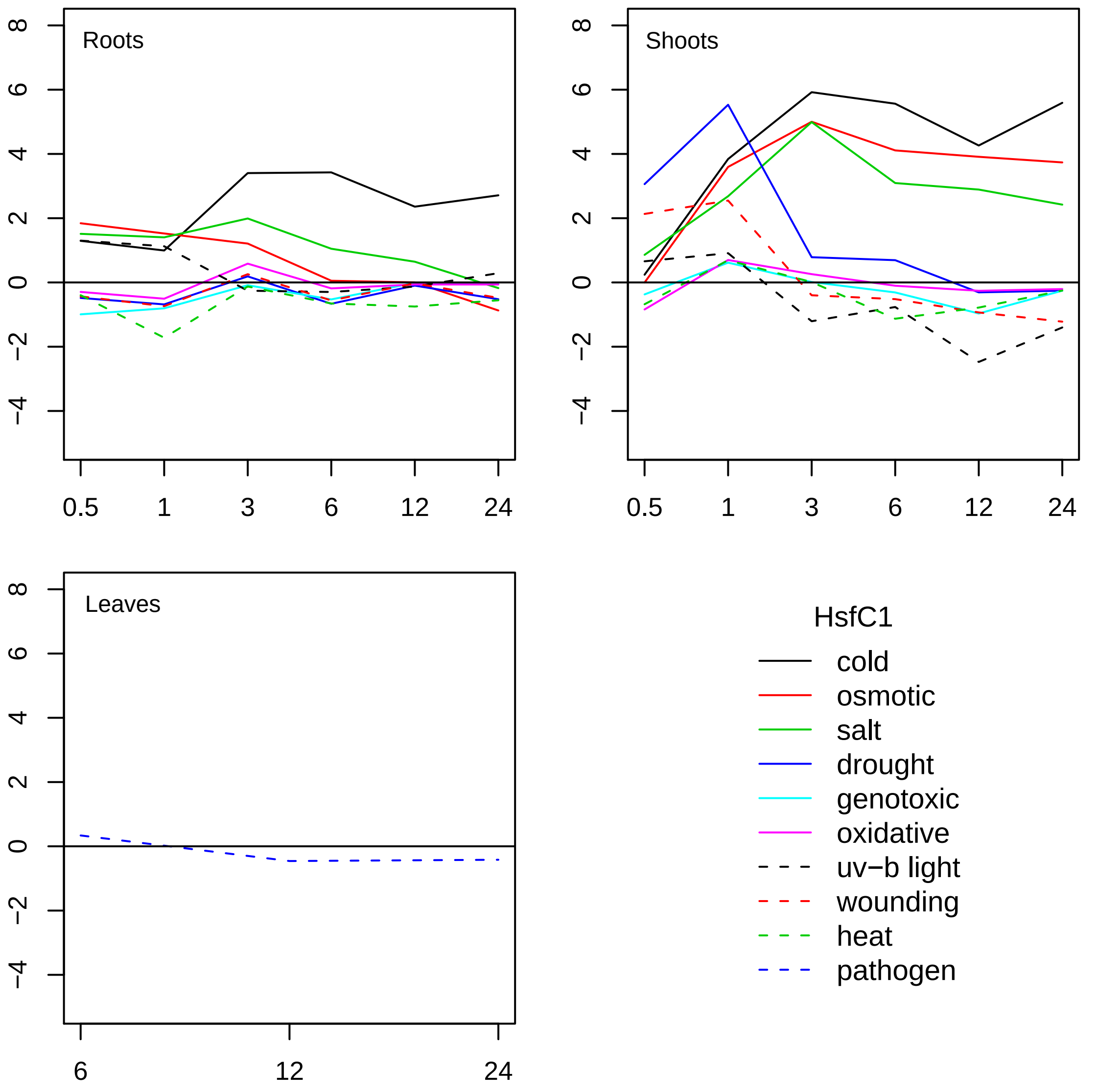
Roots
Shoots
Leaves
8
6
4
2
0
−2
−4
0.5
1
3
6
12
24
HsfC1
cold
osmotic
salt
drought
genotoxic
oxidative
uv−b light
wounding
heat
pathogen

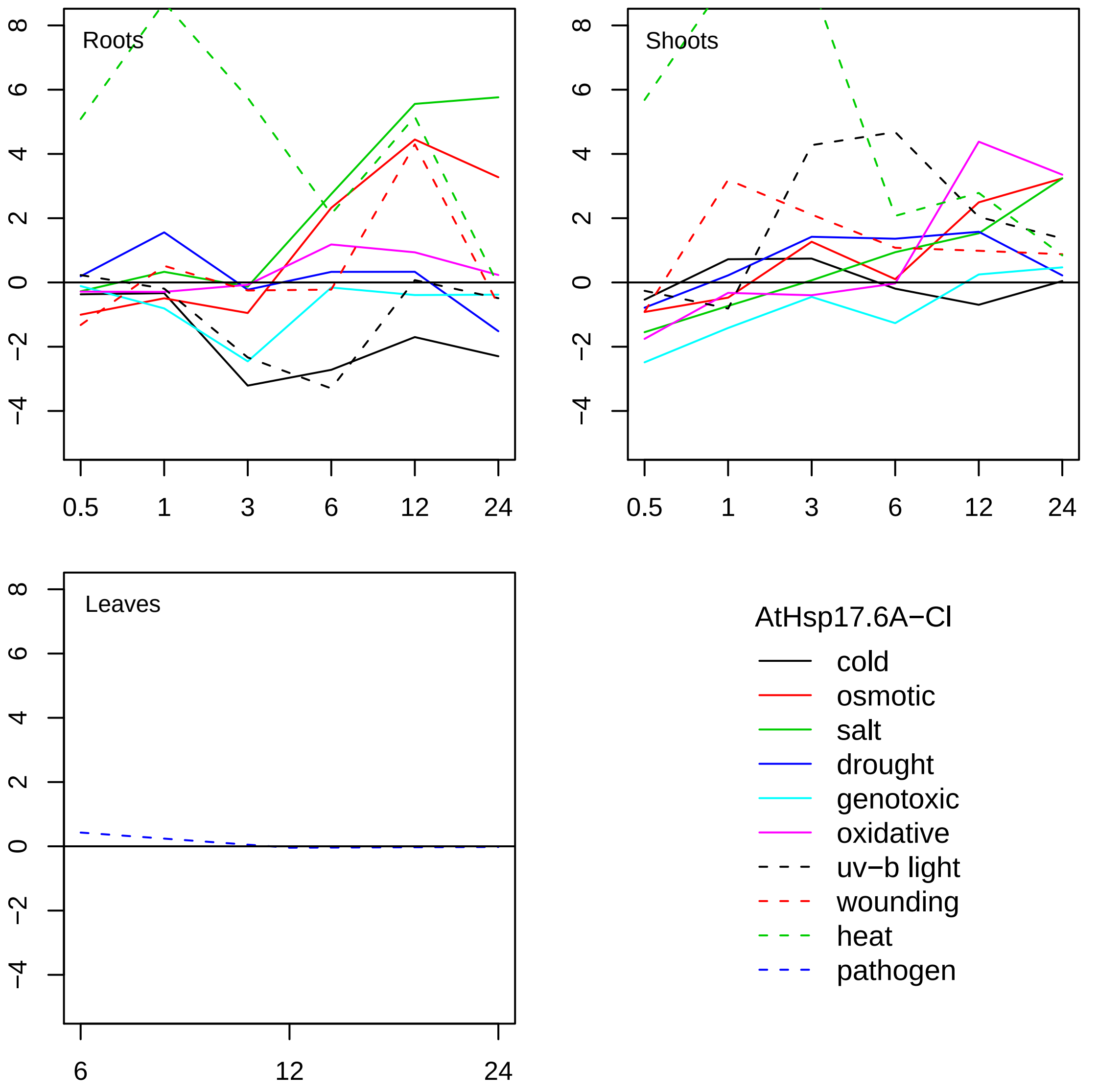

Roots
Shoots
Leaves
0.5
1
3
6
12
24
8
6
4
2
0
−2
−4
AtHsp17.6A−CI
cold
osmotic
salt
drought
genotoxic
oxidative
uv−b light
wounding
heat
pathogen

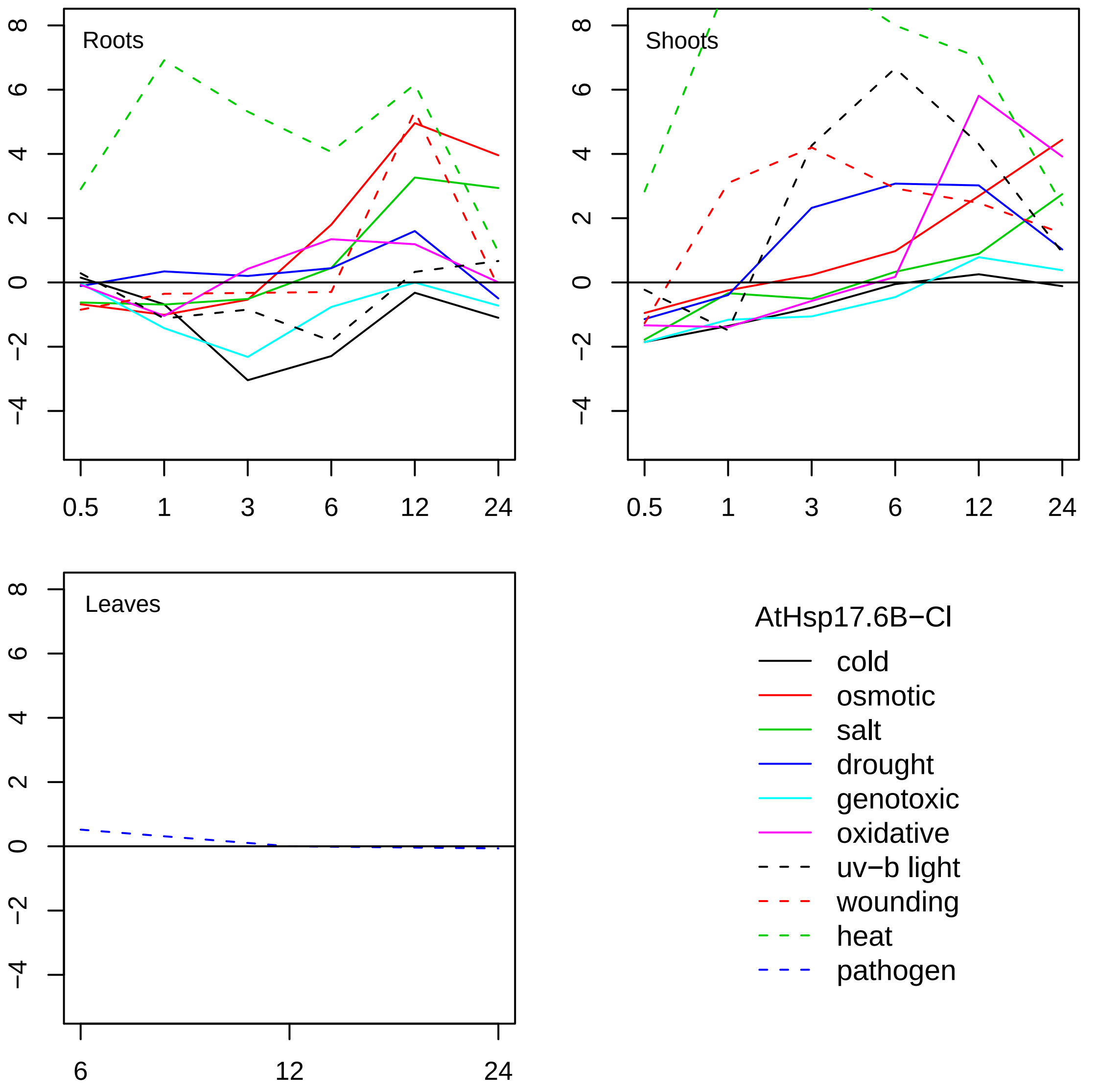
Roots
Shoots
Leaves
8
6
4
2
0
−2
−4
0.5
1
3
6
12
24
AtHsp17.6B−CI
cold
osmotic
salt
drought
genotoxic
oxidative
uv−b light
wounding
heat
pathogen

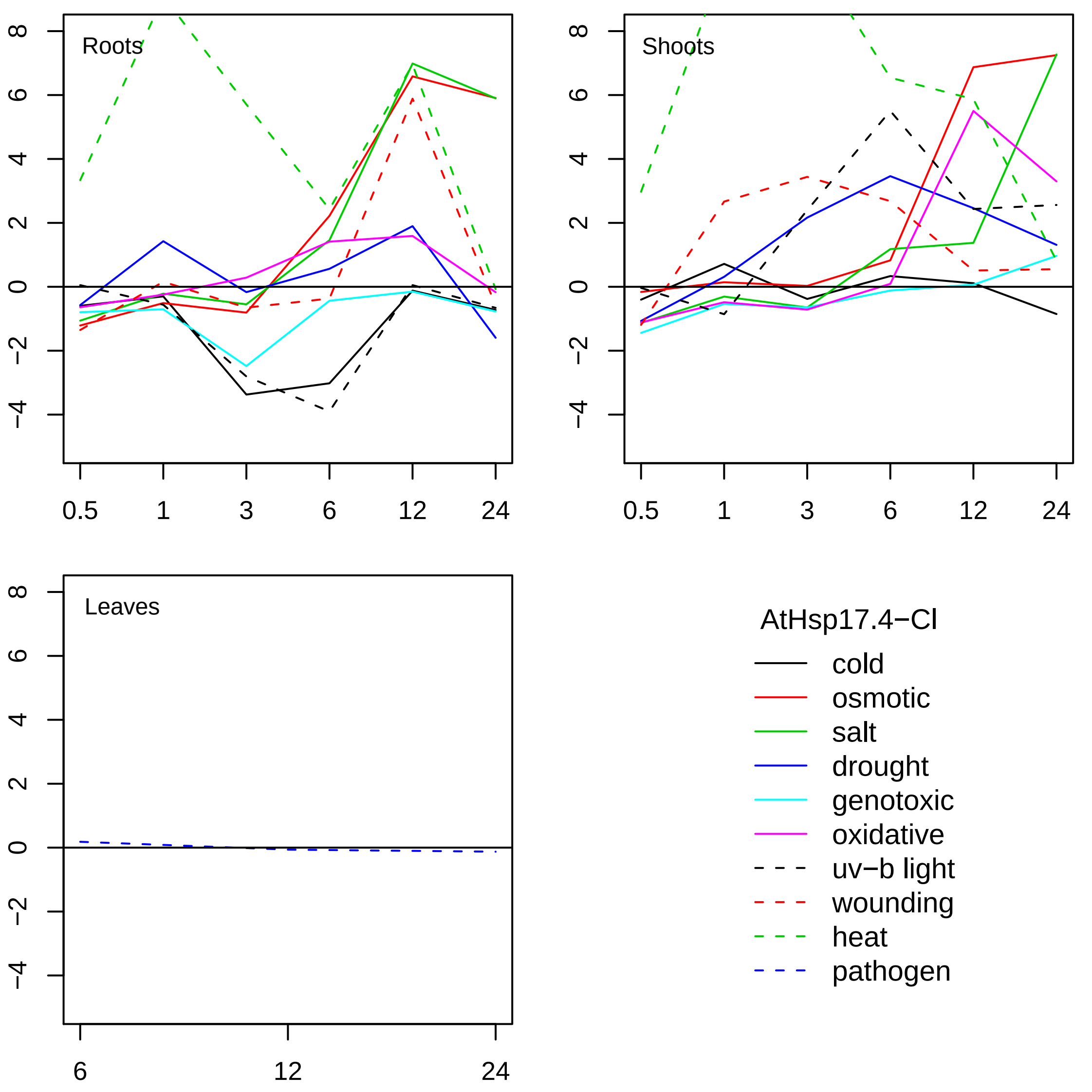
Roots
Shoots
Leaves
AtHsp17.4−CI
cold
osmotic
salt
drought
genotoxic
oxidative
uv−b light
wounding
heat
pathogen
8
6
4
2
0
−2
−4
0.5
1
3
6
12
24

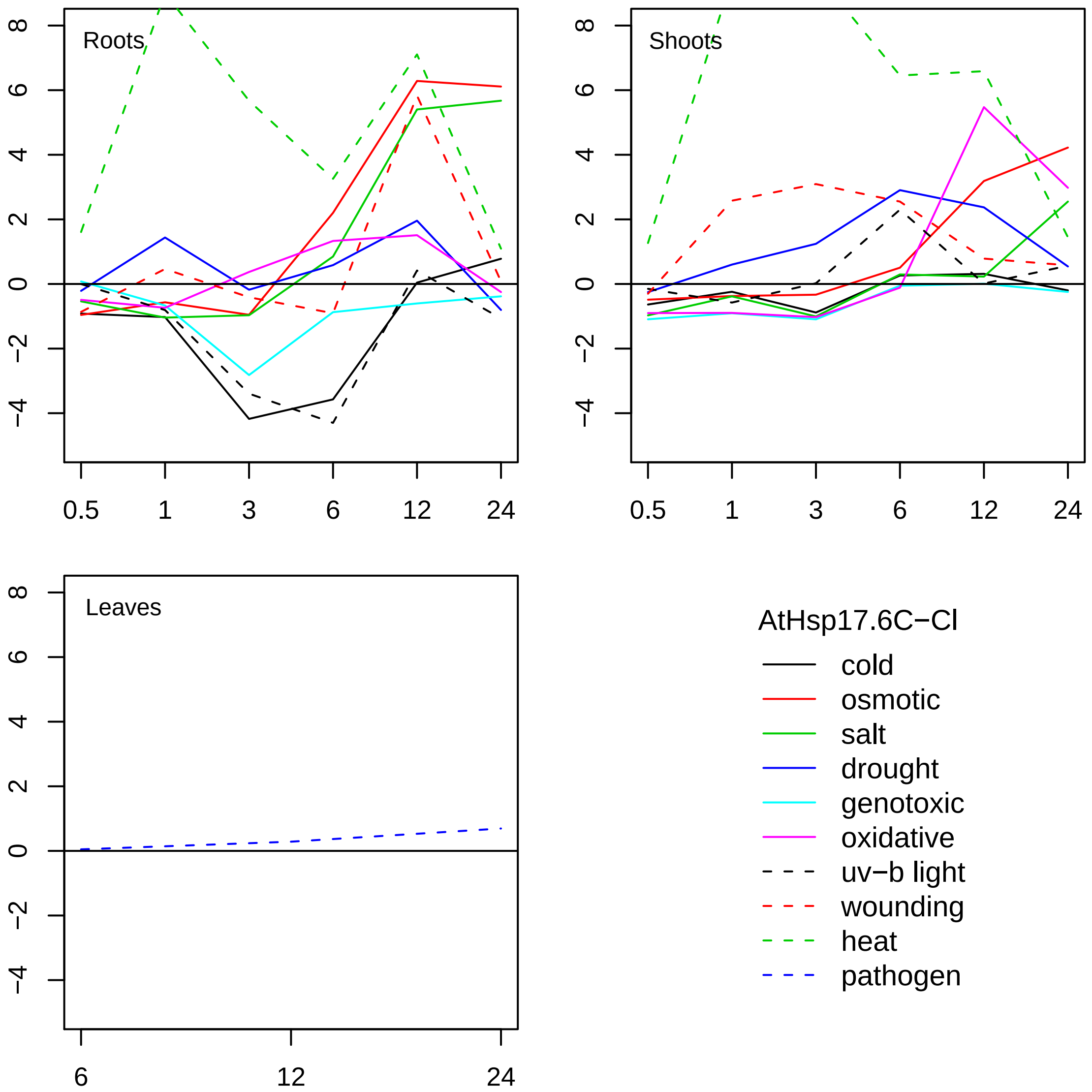

Roots
Shoots
Leaves
8
6
4
2
0
−2
−4
0.5
1
3
6
12
24
AtHsp17.6C−CI
cold
osmotic
salt
drought
genotoxic
oxidative
uv−b light
wounding
heat
pathogen

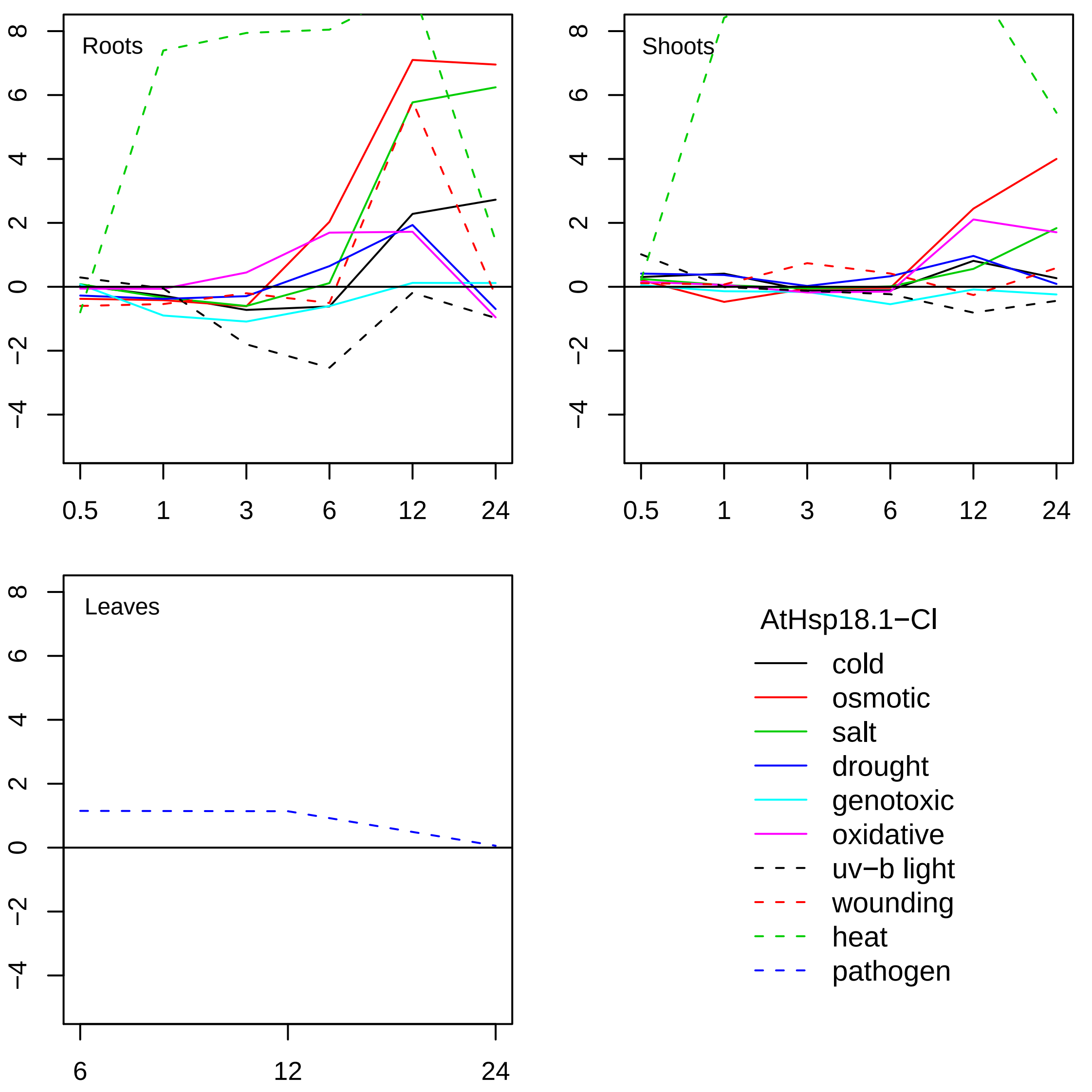

Roots
Shoots
Leaves
8
6
4
2
0
−2
−4
0.5
1
3
6
12
24
AtHsp18.1−CI
cold
osmotic
salt
drought
genotoxic
oxidative
uv−b light
wounding
heat
pathogen

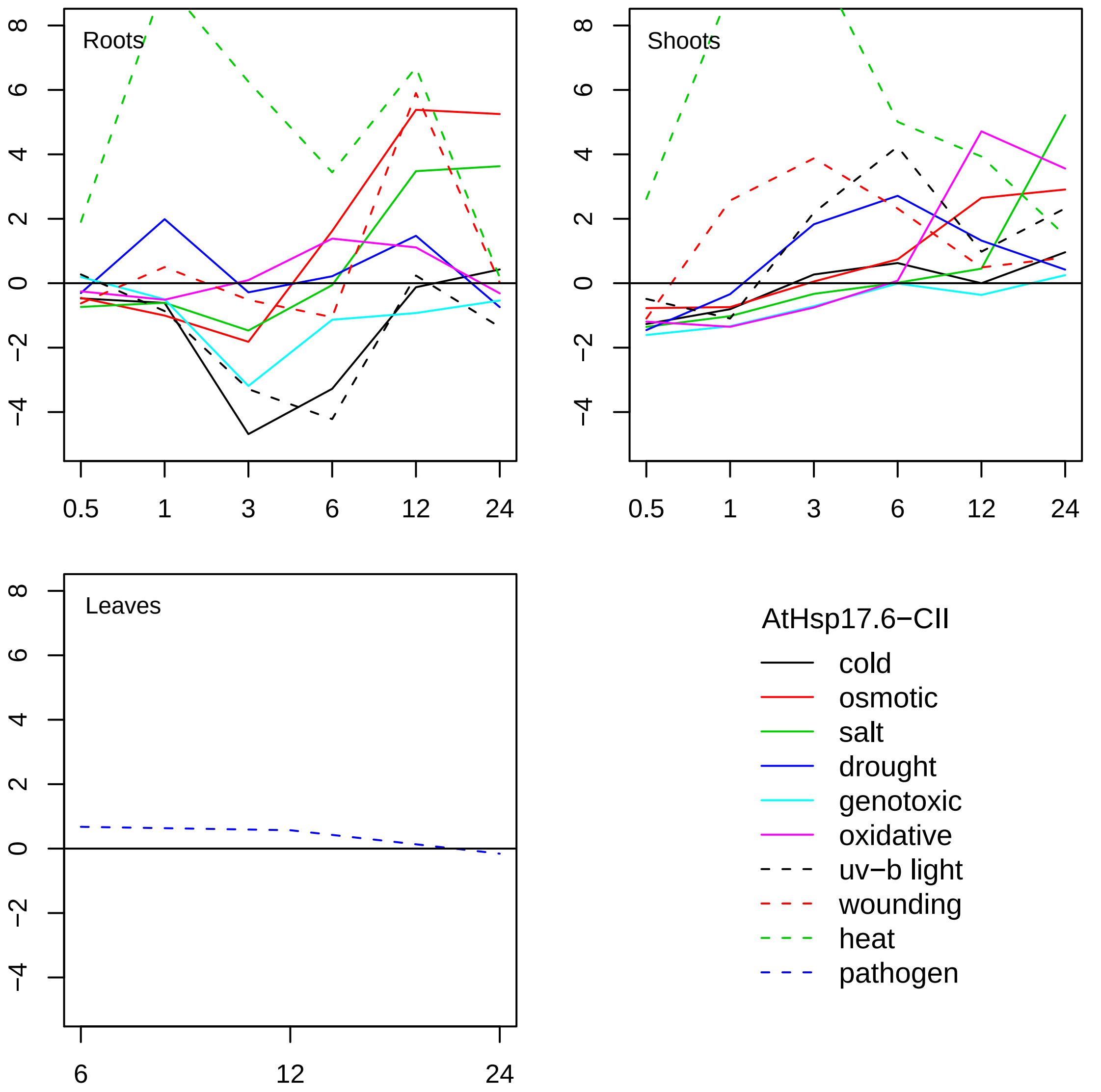

Roots
Shoots
Leaves
AtHsp17.6−CII
cold
osmotic
salt
drought
genotoxic
oxidative
uv−b light
wounding
heat
pathogen
0.5
1
3
6
12
24
−4
−2
0
2
4
6
8

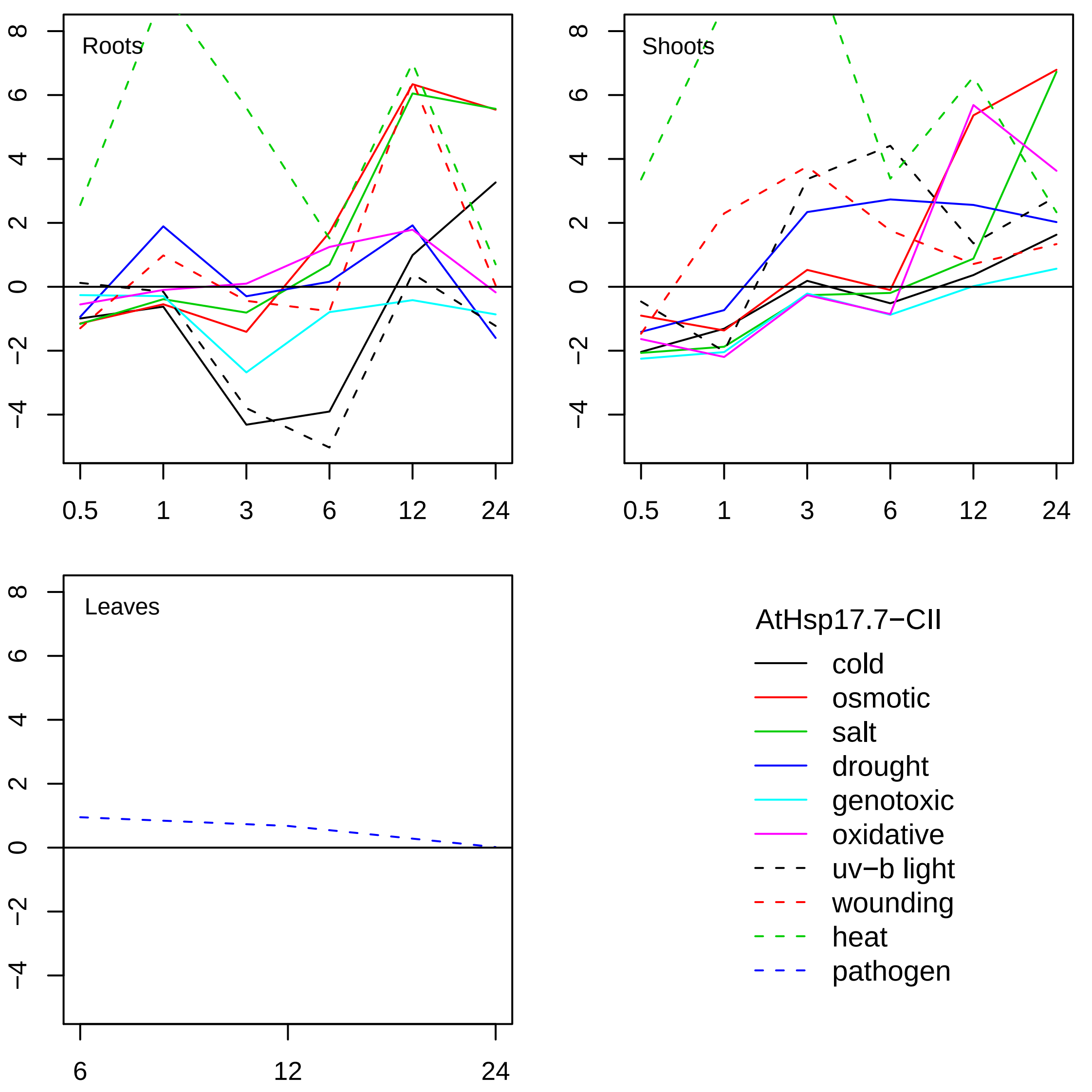

Roots
Shoots
Leaves
8
6
4
2
0
−2
−4
0.5
1
3
6
12
24
AtHsp17.7−CII
cold
osmotic
salt
drought
genotoxic
oxidative
uv−b light
wounding
heat
pathogen

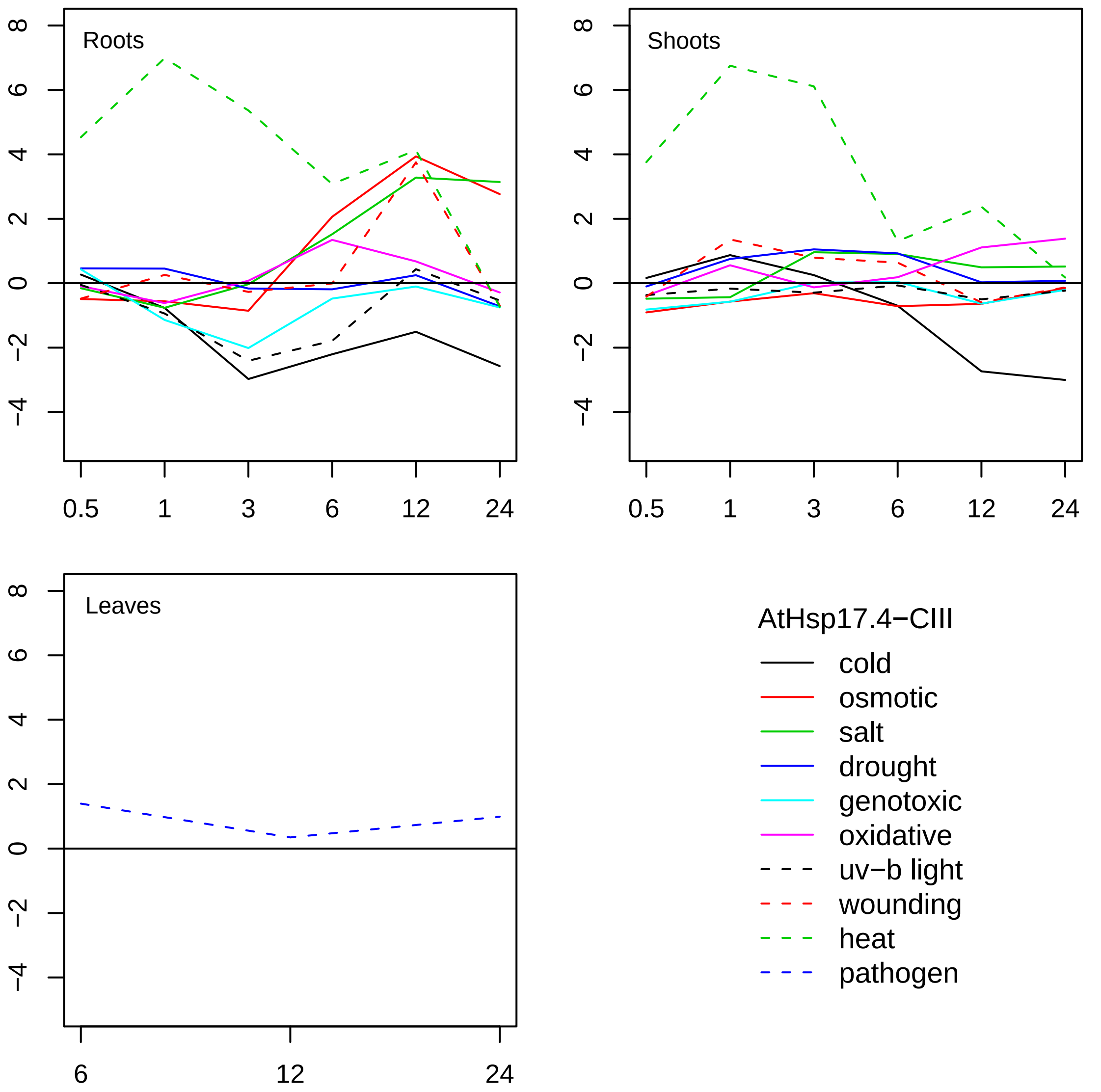

Roots
Shoots
Leaves
8
6
4
2
0
−2
−4
0.5
1
3
6
12
24
AtHsp17.4−CIII
cold
osmotic
salt
drought
genotoxic
oxidative
uv−b light
wounding
heat
pathogen

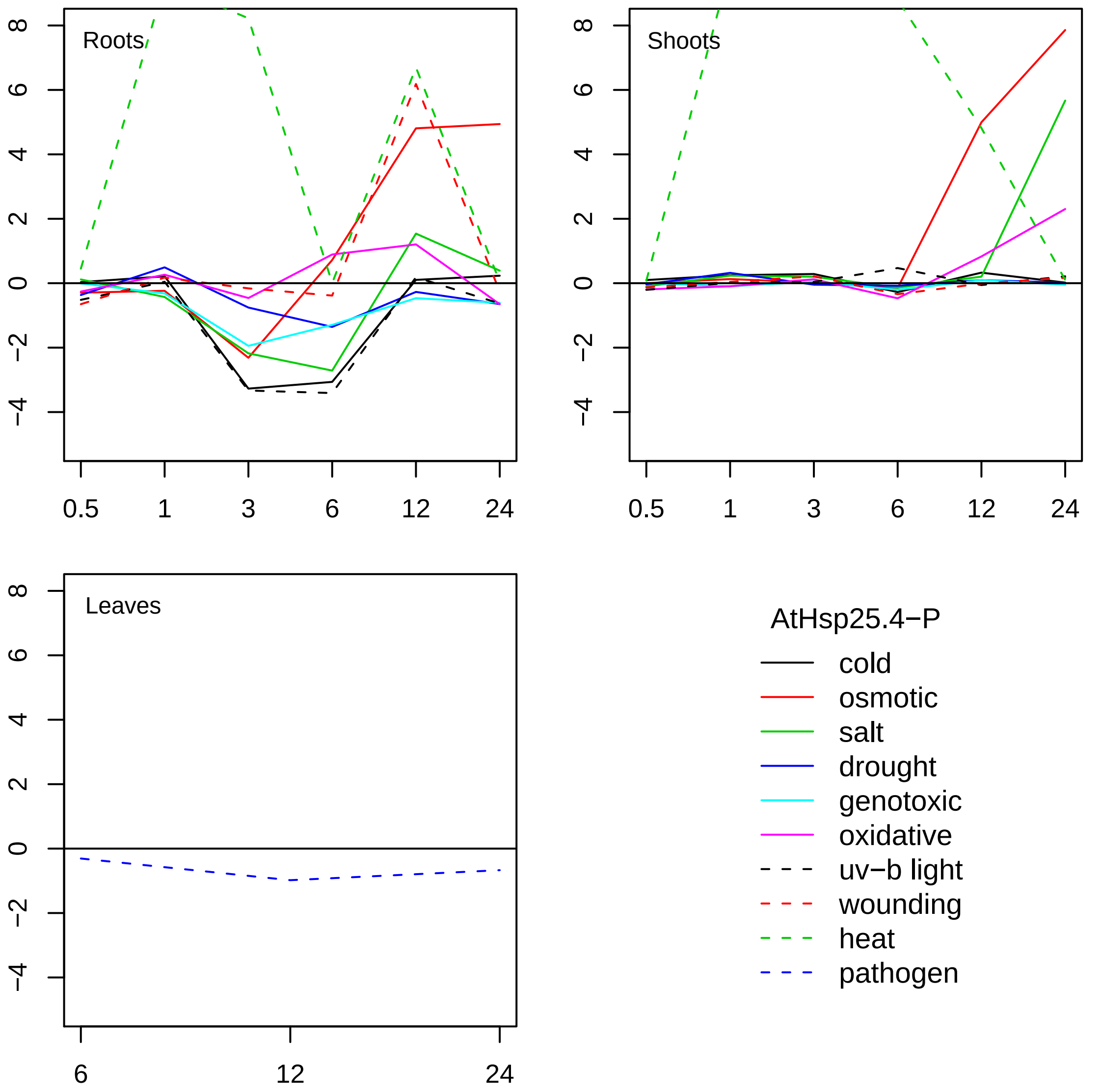
Roots
Shoots
Leaves
8
6
4
2
0
−2
−4
0.5
1
3
6
12
24
AtHsp25.4−P
cold
osmotic
salt
drought
genotoxic
oxidative
uv−b light
wounding
heat
pathogen

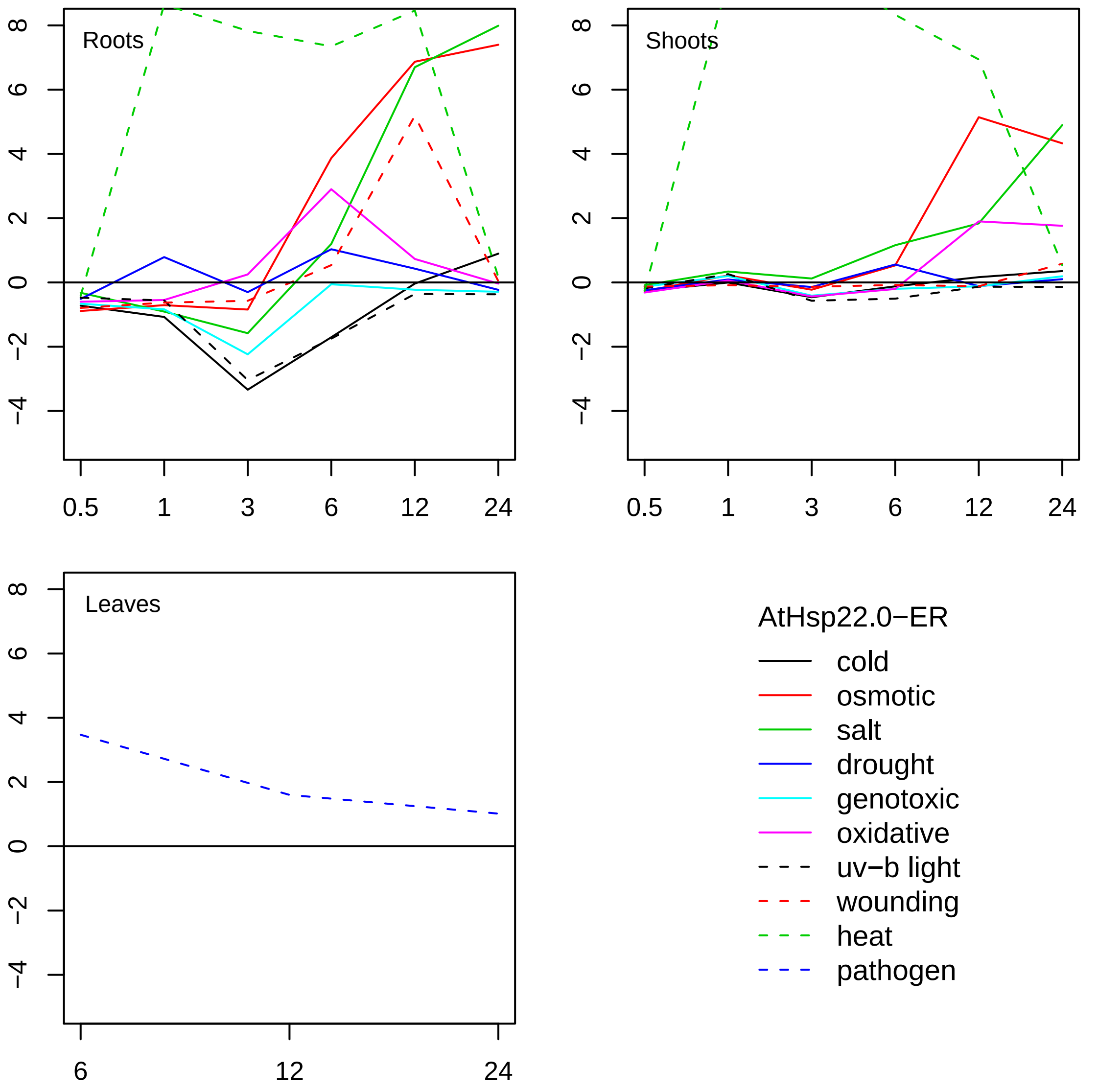
Roots
Shoots
Leaves
0.5
1
3
6
12
24
−4
−2
0
2
4
6
8
AtHsp22.0−ER
cold
osmotic
salt
drought
genotoxic
oxidative
uv−b light
wounding
heat
pathogen

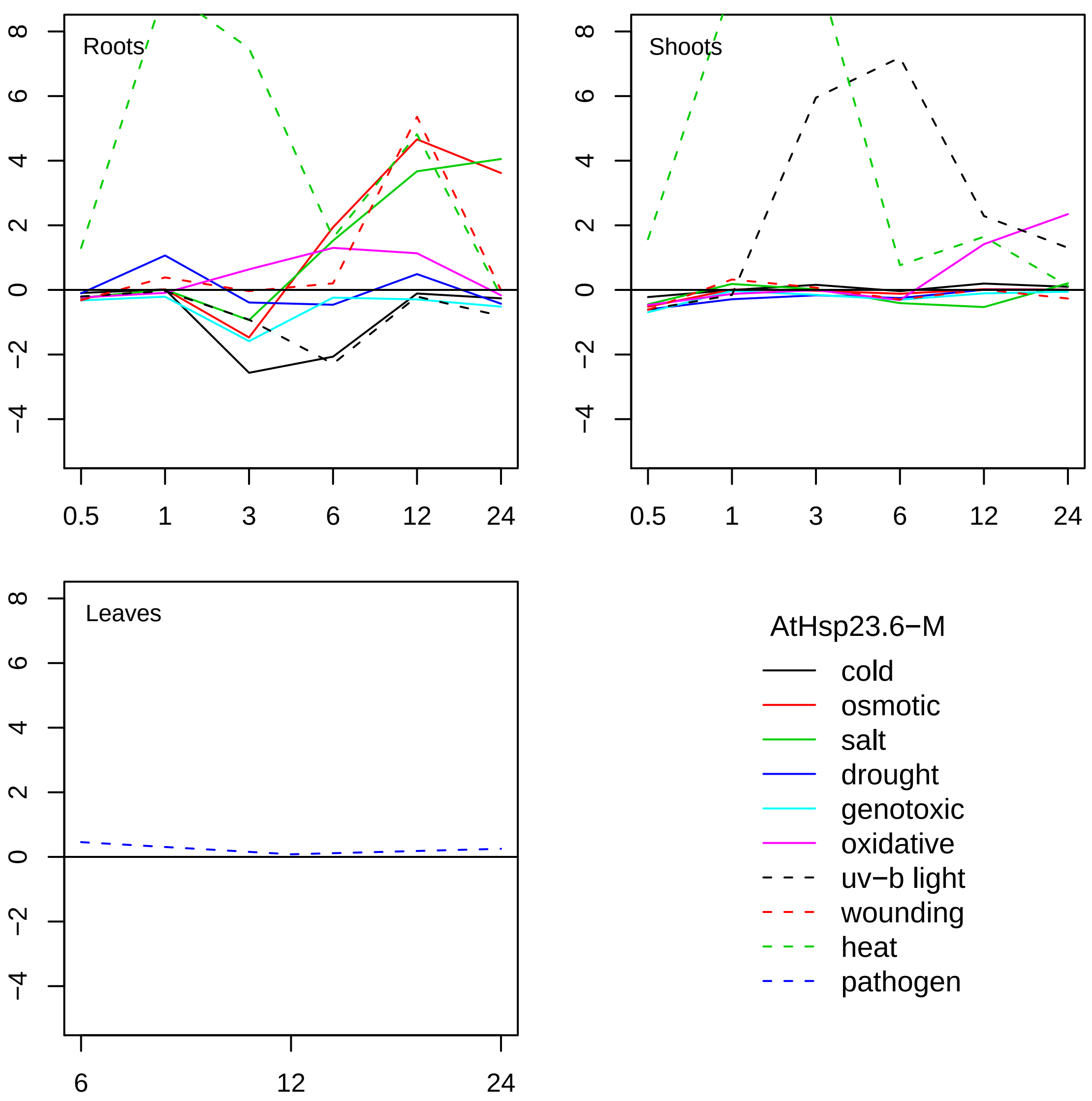
Roots
Shoots
Leaves
8
6
4
2
0
−2
−4
0.5
1
3
6
12
24
AtHsp23.6−M
cold
osmotic
salt
drought
genotoxic
oxidative
uv−b light
wounding
heat
pathogen

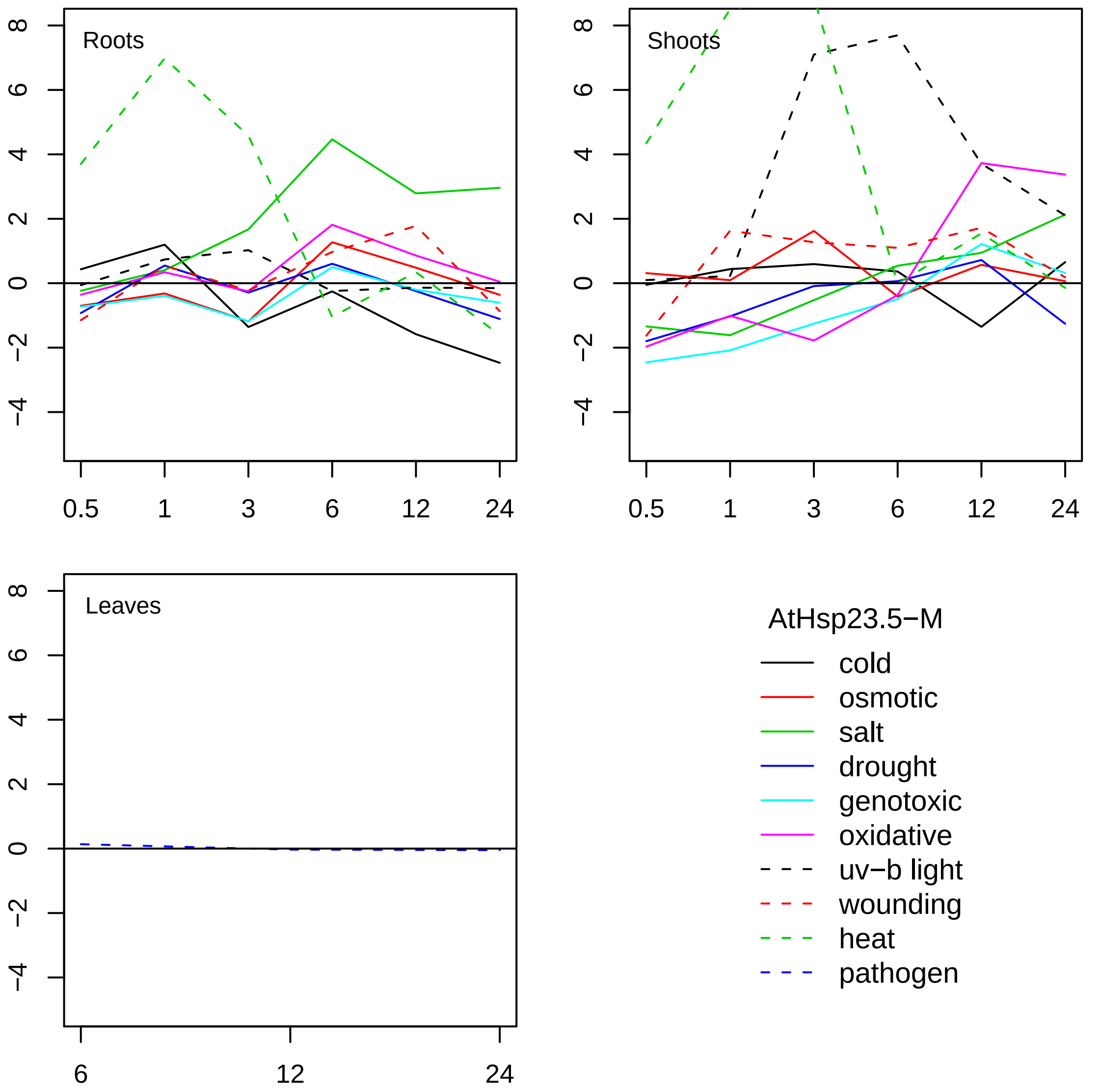
Roots
Shoots
Leaves
8
6
4
2
0
−2
−4
0.5
1
3
6
12
24
AtHsp23.5−M
cold
osmotic
salt
drought
genotoxic
oxidative
uv−b light
wounding
heat
pathogen

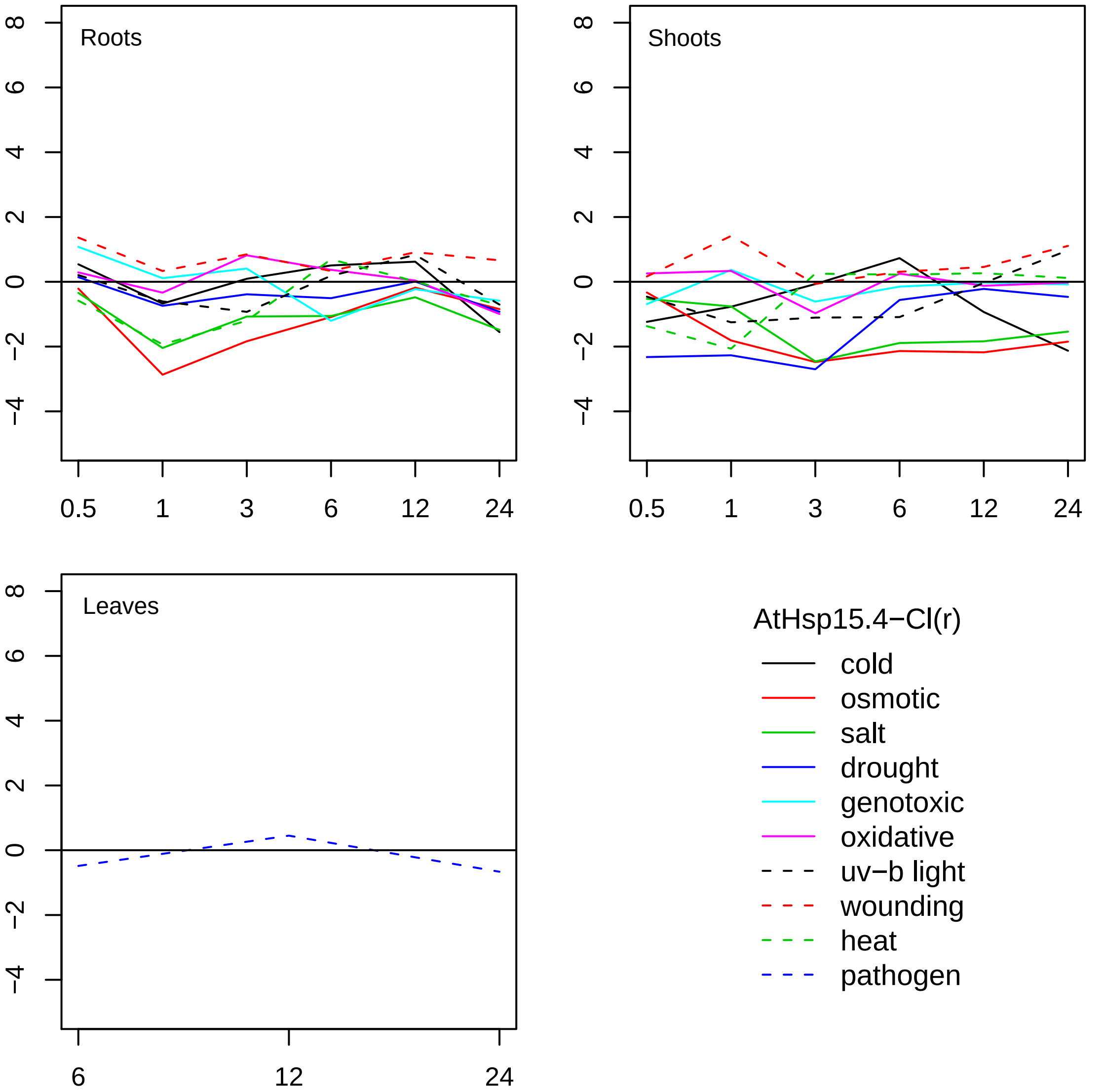
Roots
Shoots
Leaves
8
6
4
2
0
−2
−4
0.5
1
3
6
12
24
AtHsp15.4−CI(r)
cold
osmotic
salt
drought
genotoxic
oxidative
uv−b light
wounding
heat
pathogen

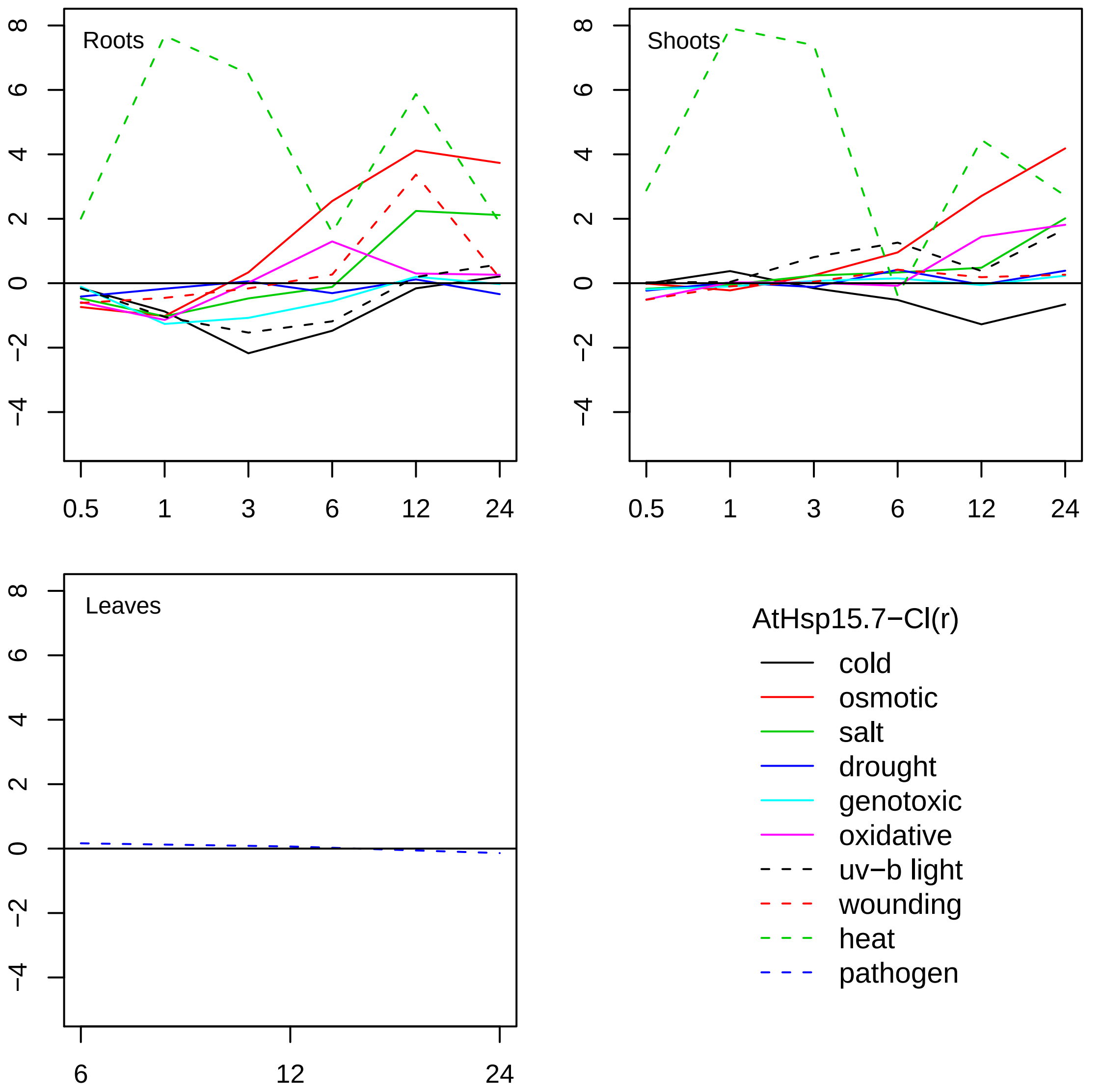

Roots
Shoots
Leaves
8
6
4
2
0
−2
−4
0.5
1
3
6
12
24
AtHsp15.7−CI(r)
cold
osmotic
salt
drought
genotoxic
oxidative
uv−b light
wounding
heat
pathogen

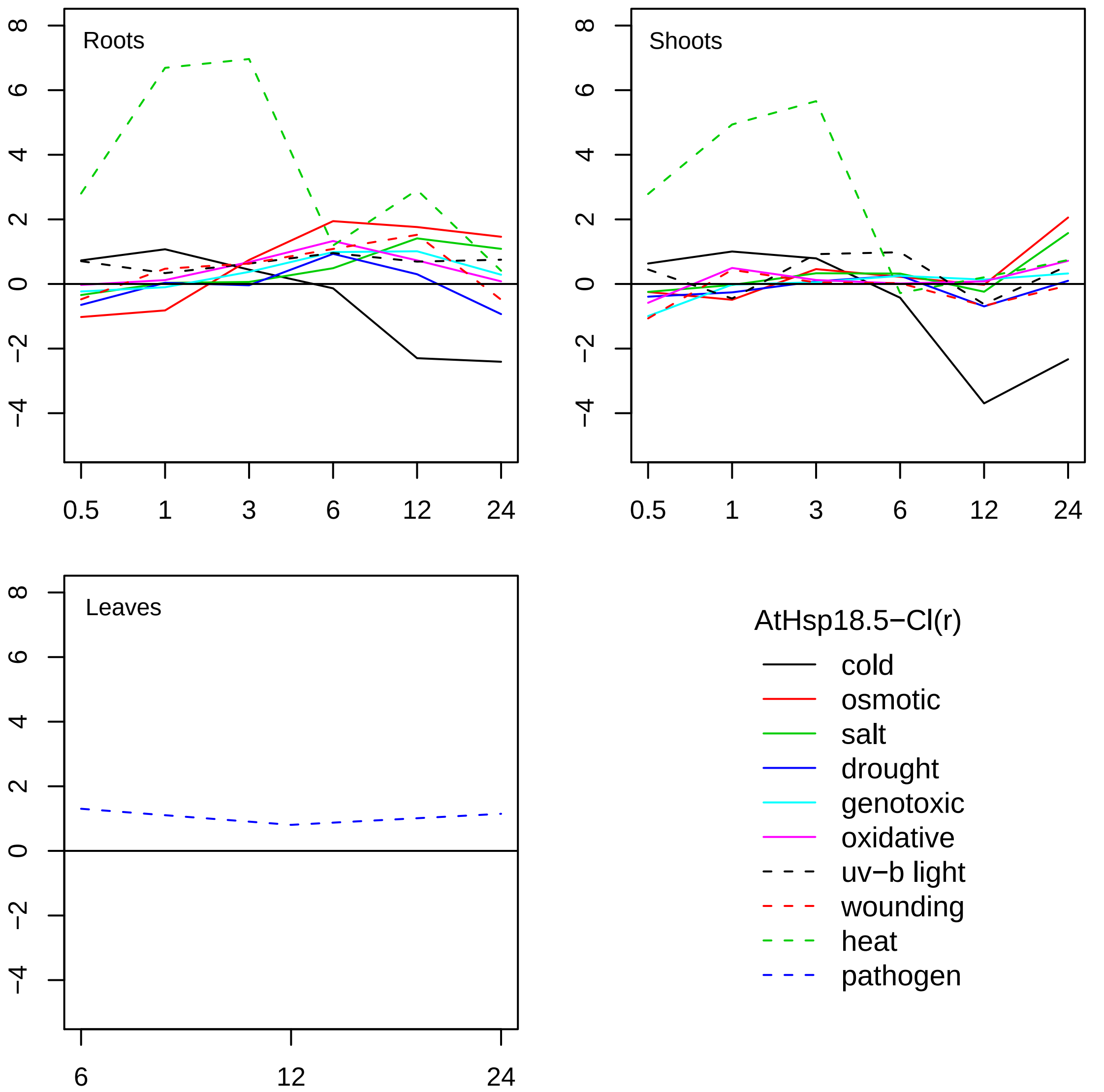
Roots
Shoots
Leaves
8
6
4
2
0
−2
−4
0.5
1
3
6
12
24
AtHsp18.5−CI(r)
cold
osmotic
salt
drought
genotoxic
oxidative
uv−b light
wounding
heat
pathogen

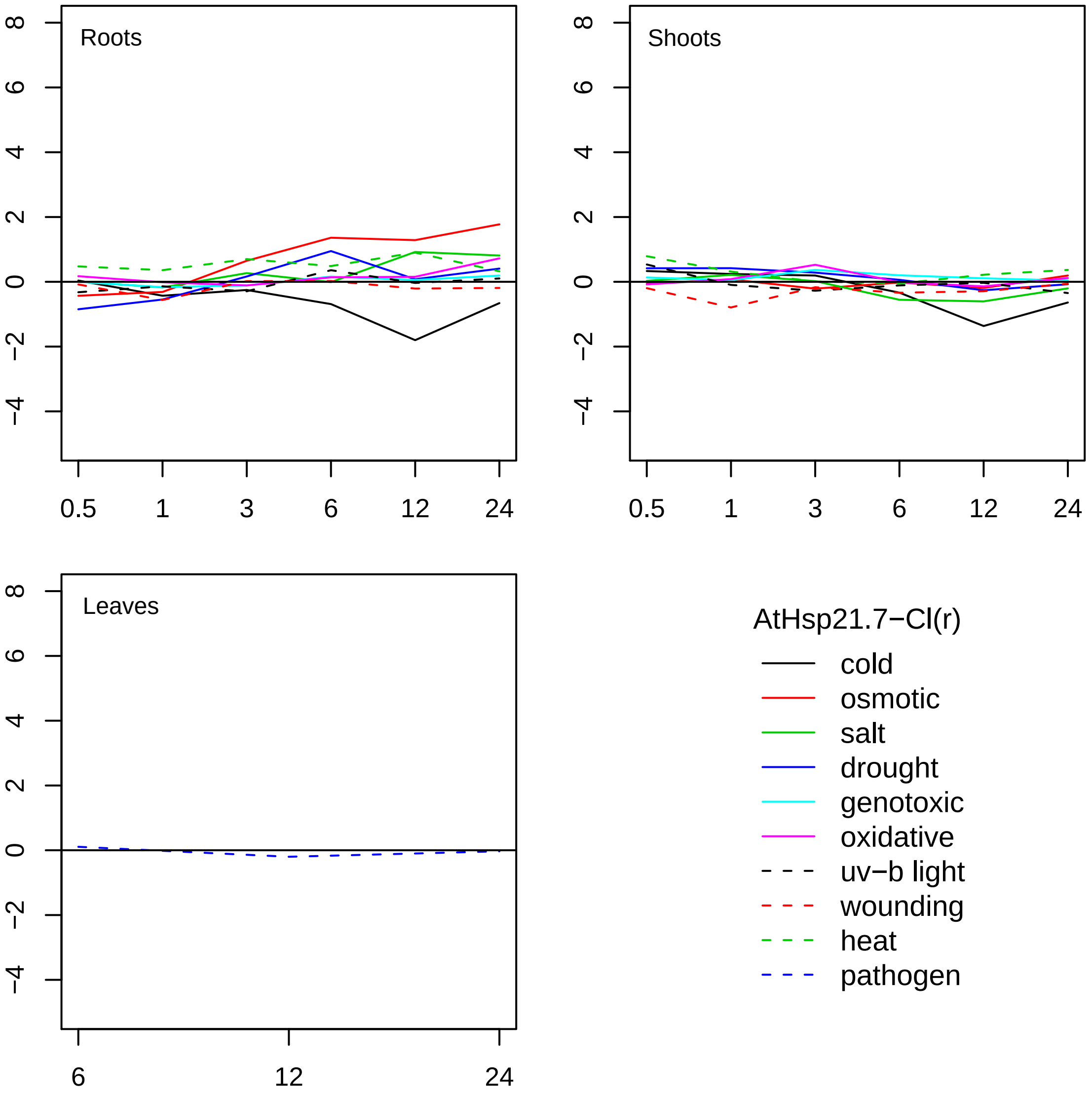
Roots
Shoots
Leaves
8
6
4
2
0
−2
−4
0.5
1
3
6
12
24
AtHsp21.7−CI(r)
cold
osmotic
salt
drought
genotoxic
oxidative
uv−b light
wounding
heat
pathogen

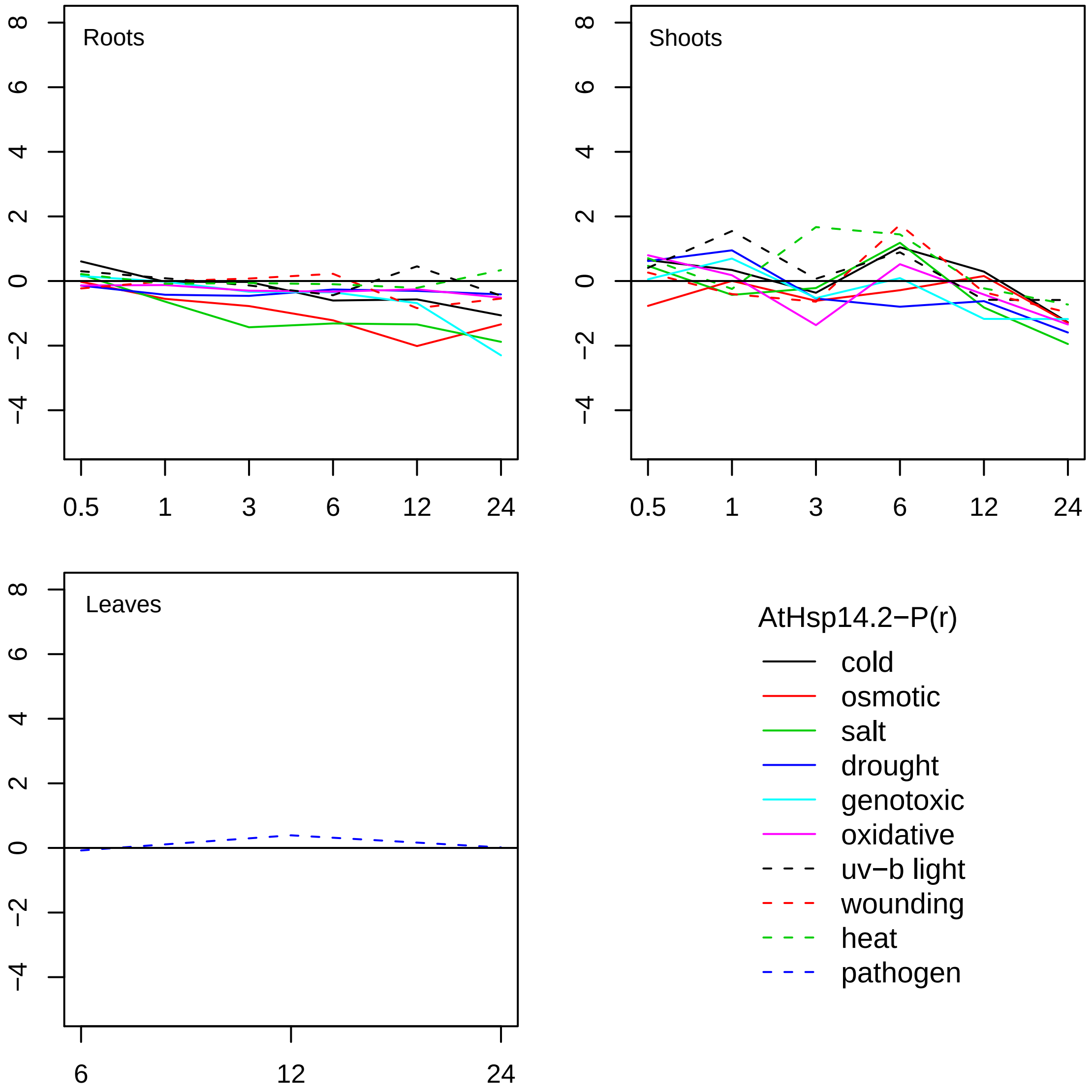

Roots
Shoots
Leaves
8
6
4
2
0
−2
−4
0.5
1
3
6
12
24
AtHsp14.2−P(r)
cold
osmotic
salt
drought
genotoxic
oxidative
uv−b light
wounding
heat
pathogen

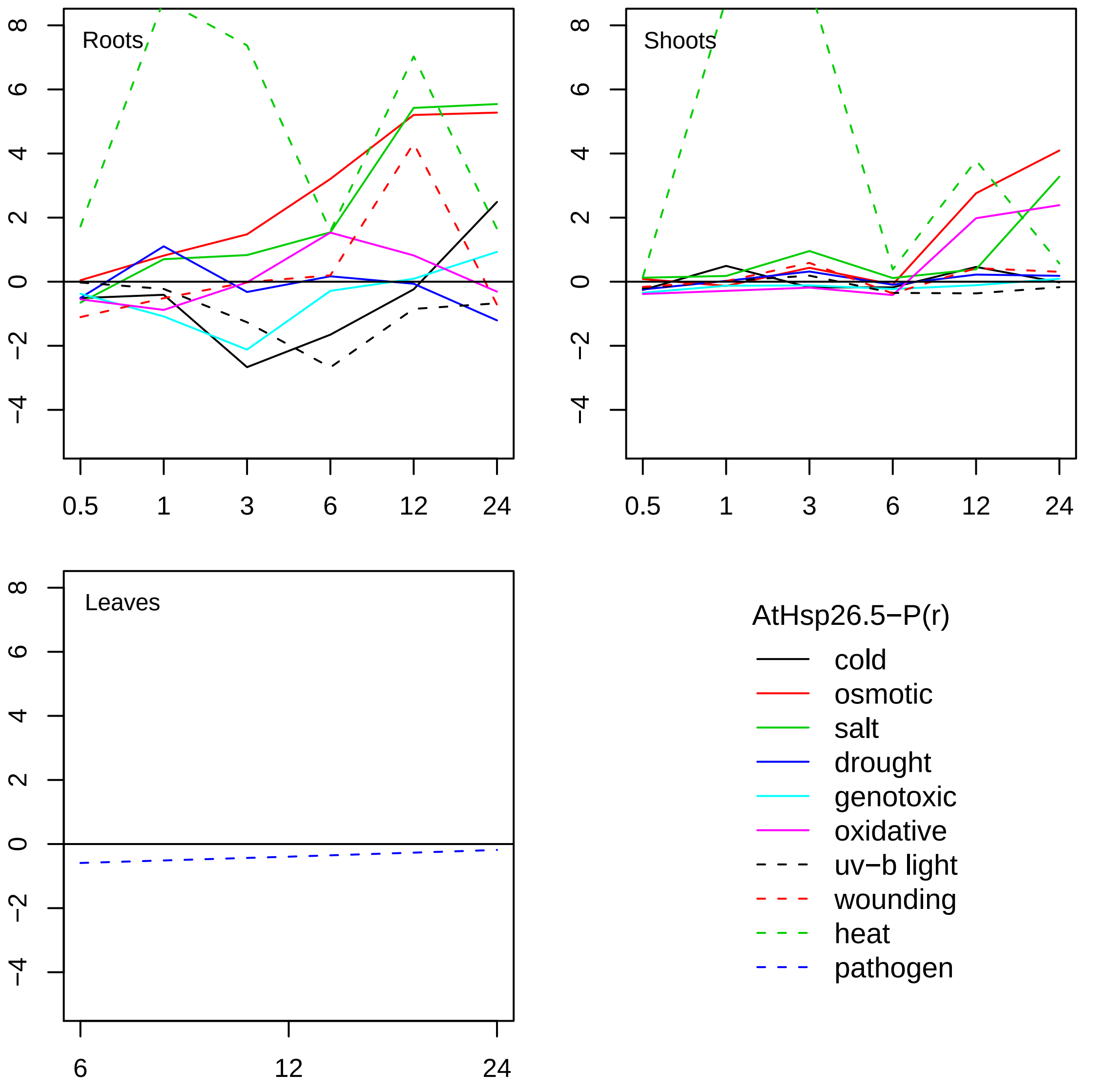
Roots
Shoots
Leaves
AtHsp26.5−P(r)
cold
osmotic
salt
drought
genotoxic
oxidative
uv−b light
wounding
heat
pathogen
0.5
1
3
6
12
24
−4
−2
0
2
4
6
8

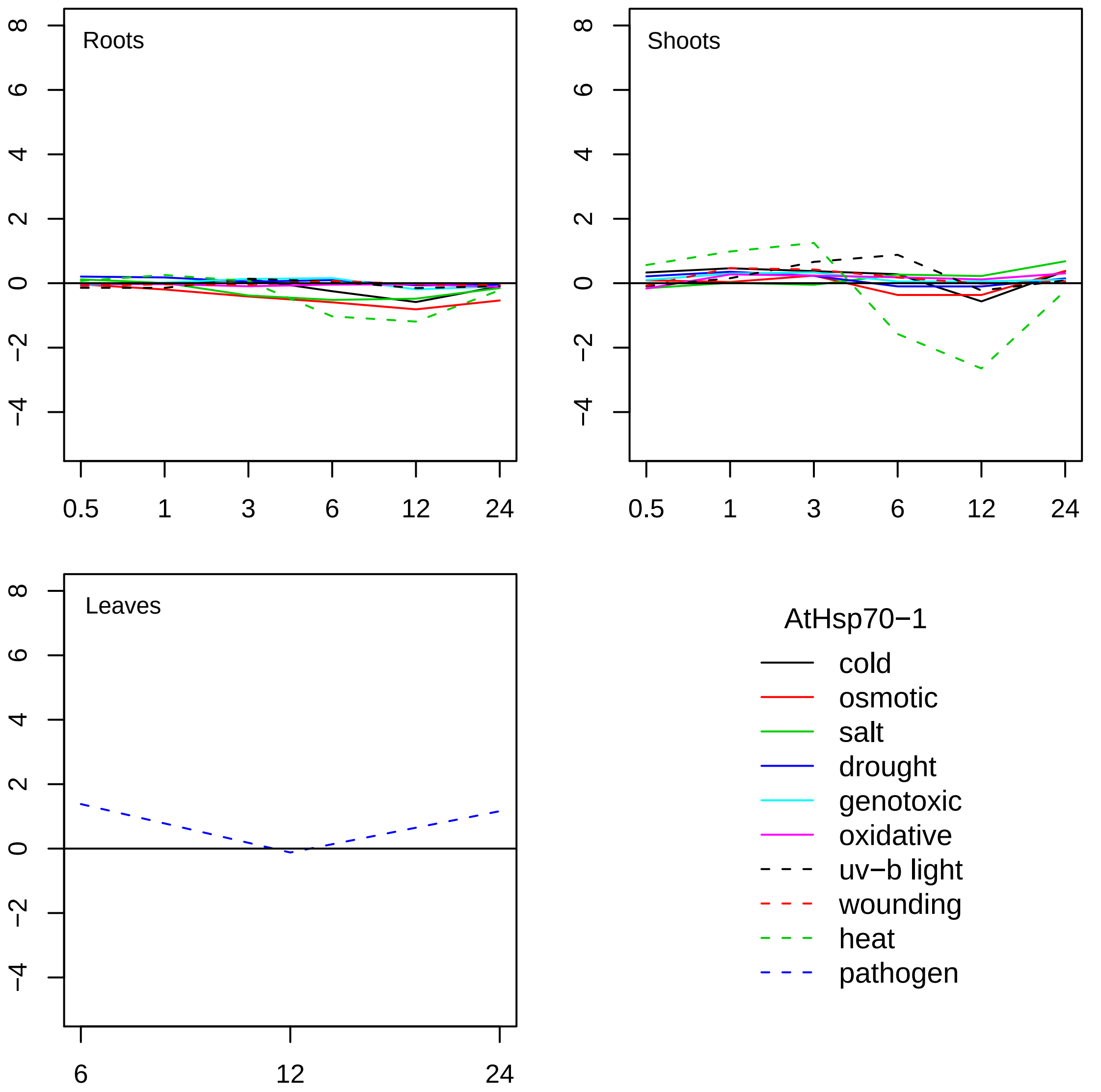

Roots
Shoots
Leaves
8
6
4
2
0
−2
−4
0.5
1
3
6
12
24
AtHsp70−1
cold
osmotic
salt
drought
genotoxic
oxidative
uv−b light
wounding
heat
pathogen

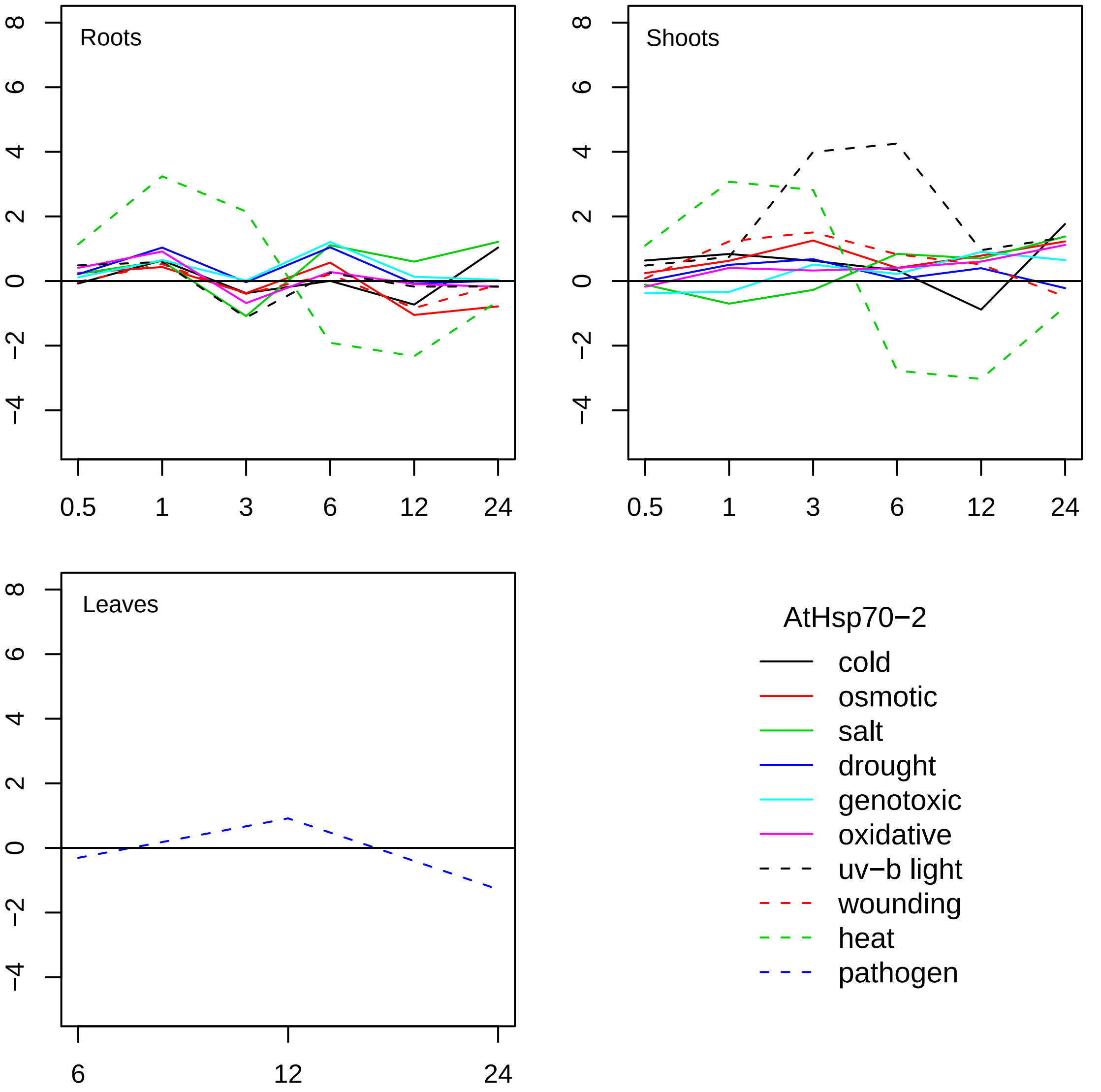
Roots
Shoots
Leaves
8
6
4
2
0
−2
−4
0.5
1
3
6
12
24
AtHsp70−2
cold
osmotic
salt
drought
genotoxic
oxidative
uv−b light
wounding
heat
pathogen

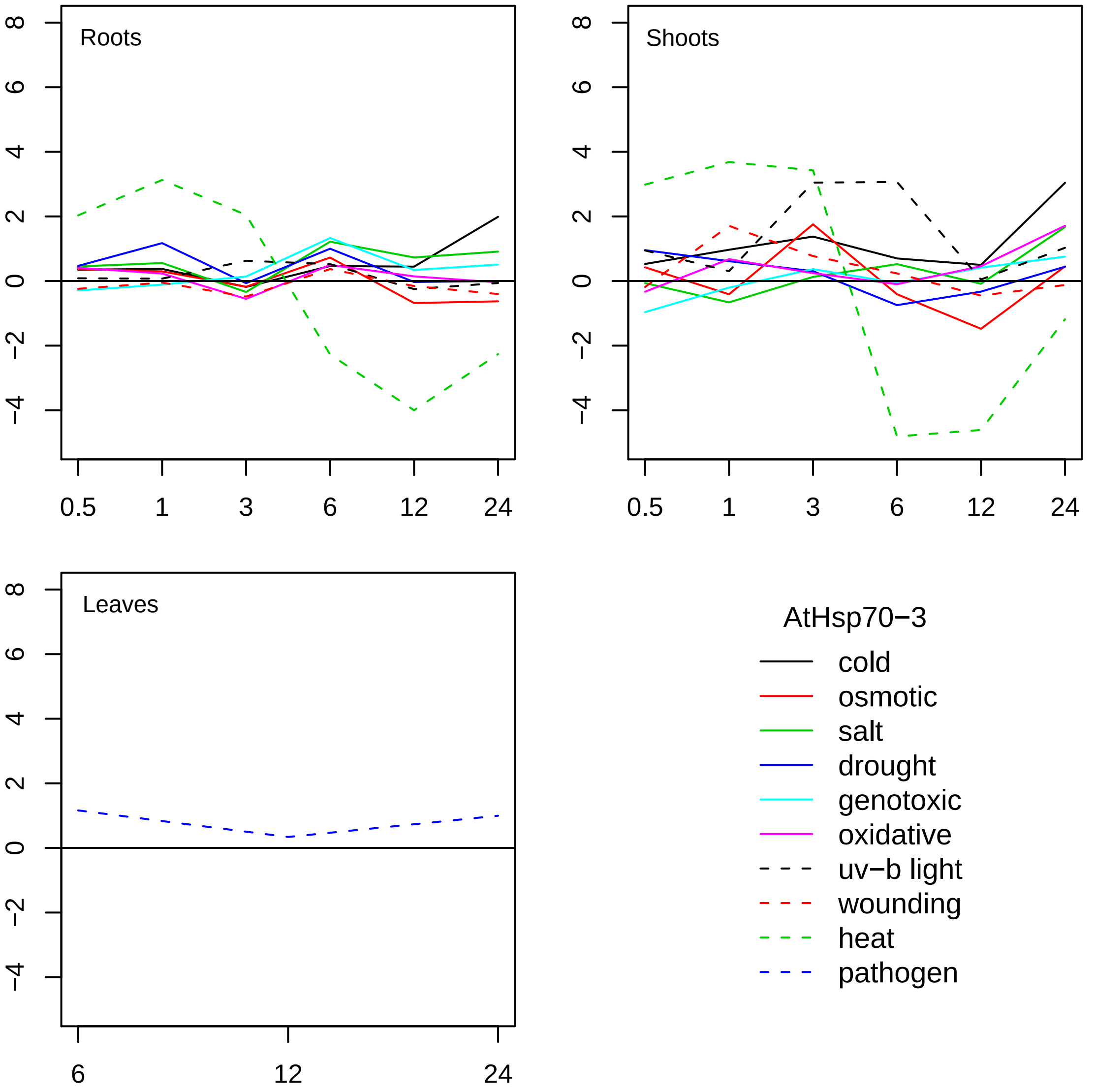

Roots
Shoots
Leaves
8
6
4
2
0
−2
−4
0.5
1
3
6
12
24
AtHsp70−3
cold
osmotic
salt
drought
genotoxic
oxidative
uv−b light
wounding
heat
pathogen

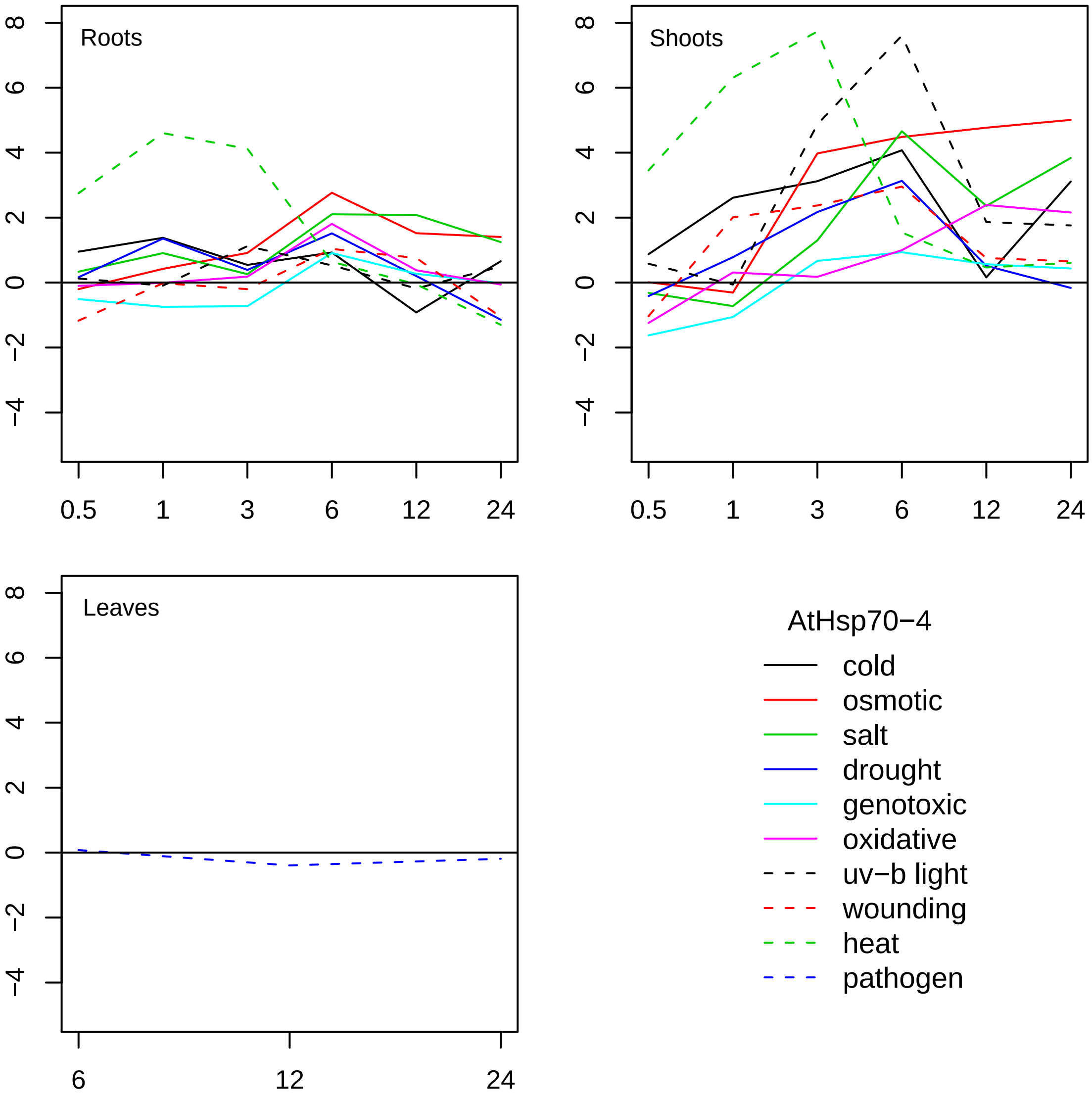
Roots
Shoots
Leaves
AtHsp70−4
cold
osmotic
salt
drought
genotoxic
oxidative
uv−b light
wounding
heat
pathogen
0.5
1
3
6
12
24
8
6
4
2
0
−2
−4

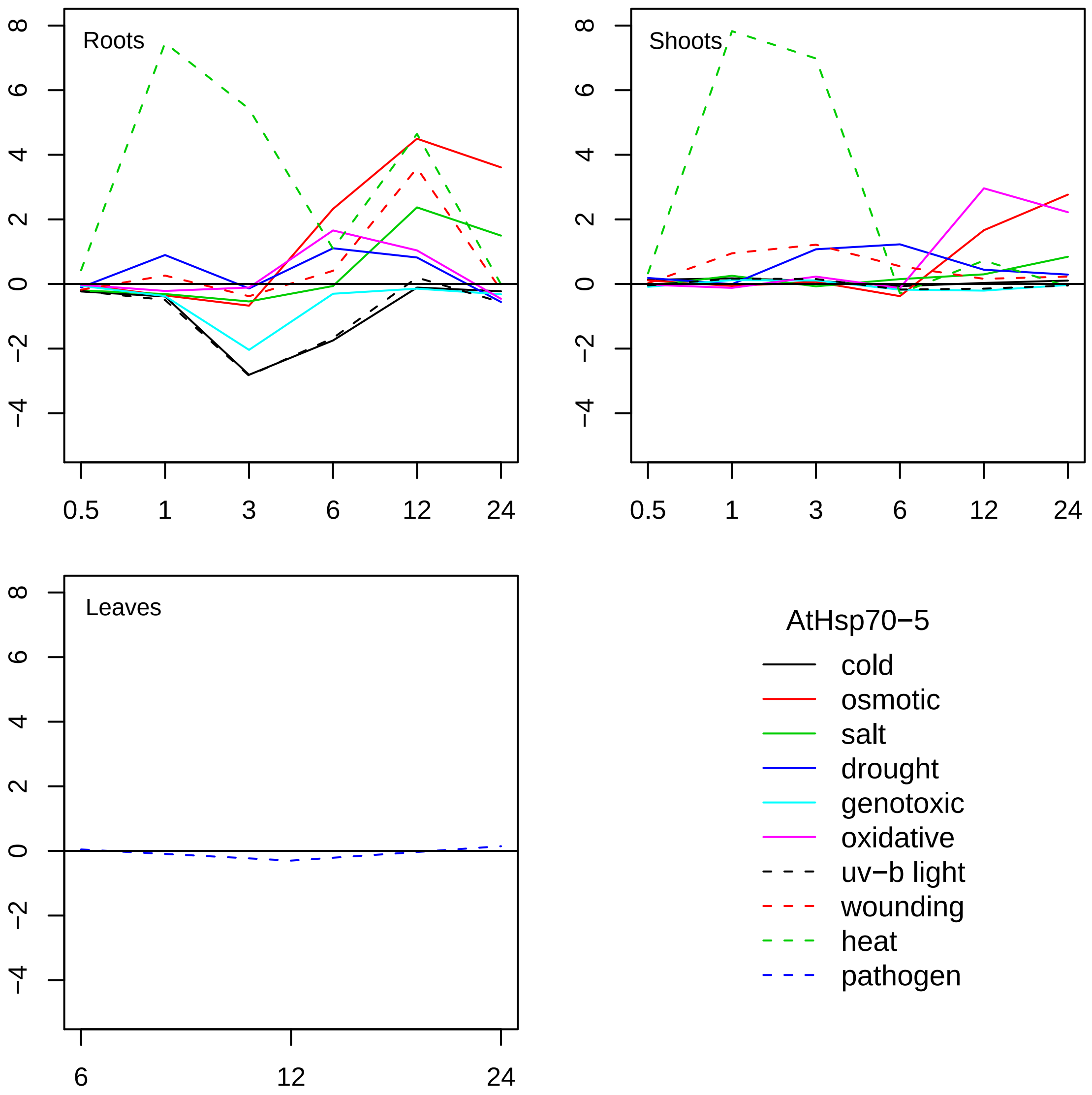

Roots
Shoots
Leaves
8
6
4
2
0
−2
−4
0.5
1
3
6
12
24
AtHsp70−5
cold
osmotic
salt
drought
genotoxic
oxidative
uv−b light
wounding
heat
pathogen

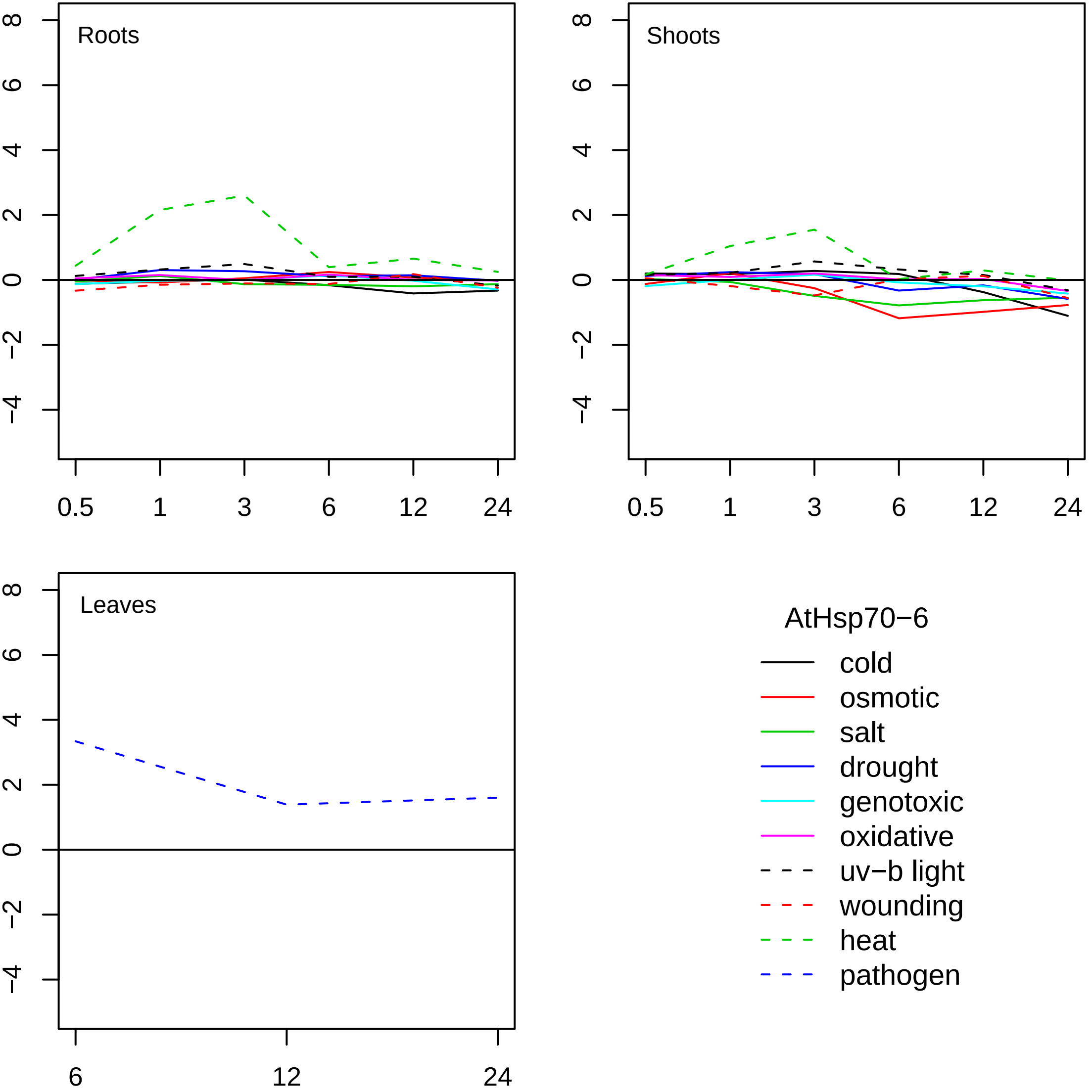

Roots
Shoots
Leaves
8
6
4
2
0
−2
−4
0.5
1
3
6
12
24
AtHsp70−6
cold
osmotic
salt
drought
genotoxic
oxidative
uv−b light
wounding
heat
pathogen

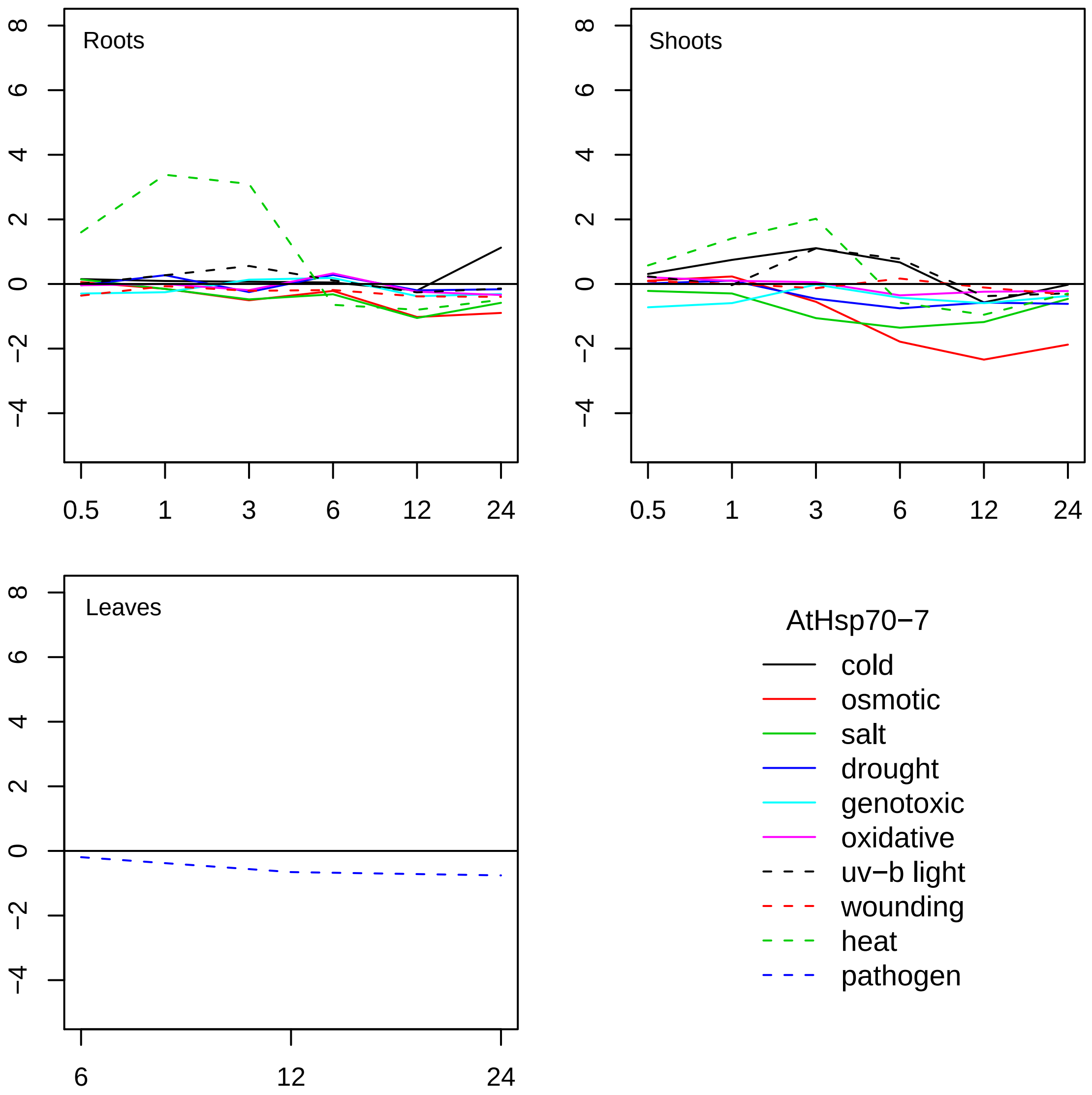
Roots
Shoots
Leaves
8
6
4
2
0
−2
−4
0.5
1
3
6
12
24
AtHsp70−7
cold
osmotic
salt
drought
genotoxic
oxidative
uv−b light
wounding
heat
pathogen

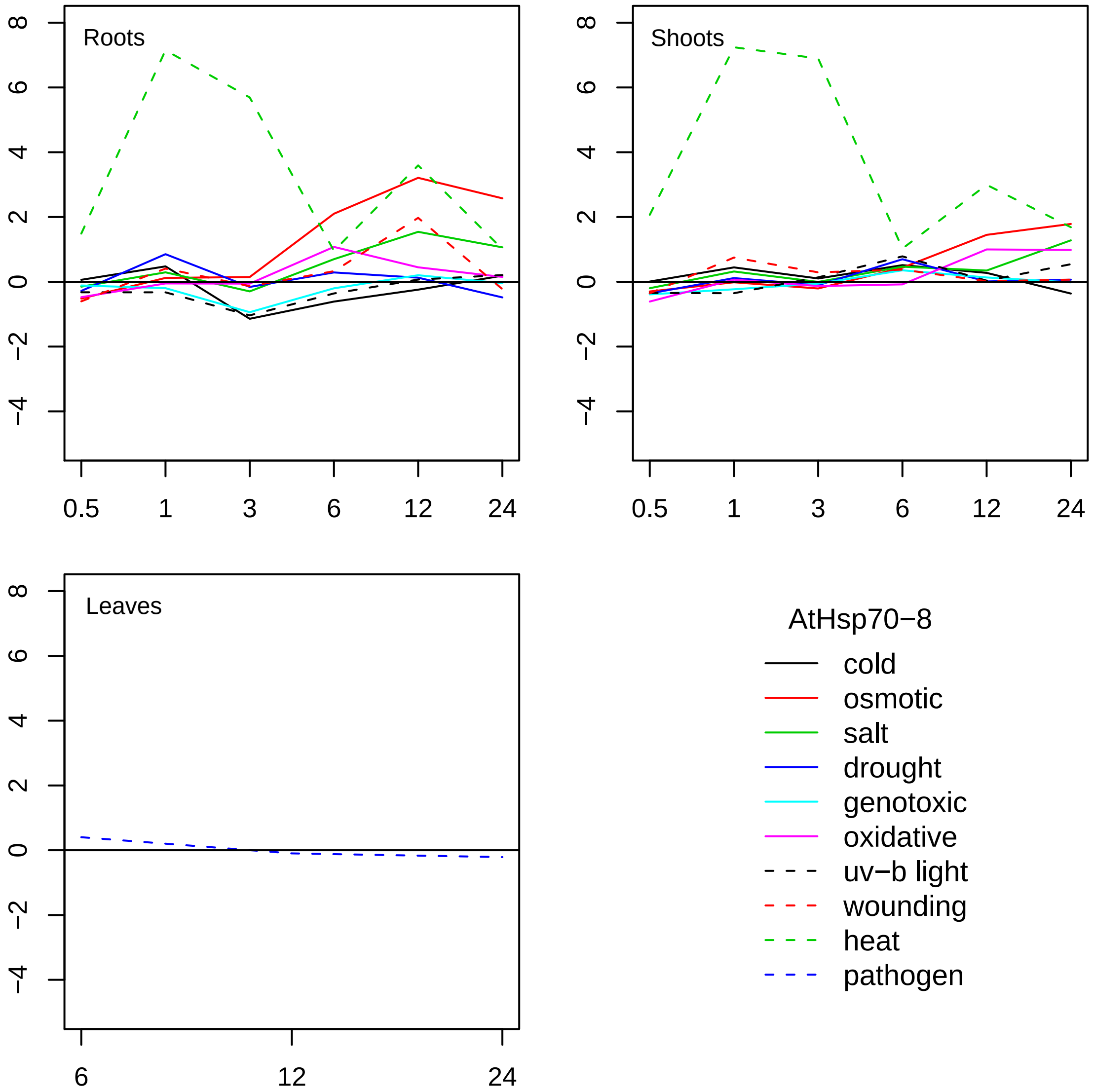

Roots
Shoots
Leaves
8
6
4
2
0
−2
−4
0.5
1
3
6
12
24
AtHsp70−8
cold
osmotic
salt
drought
genotoxic
oxidative
uv−b light
wounding
heat
pathogen

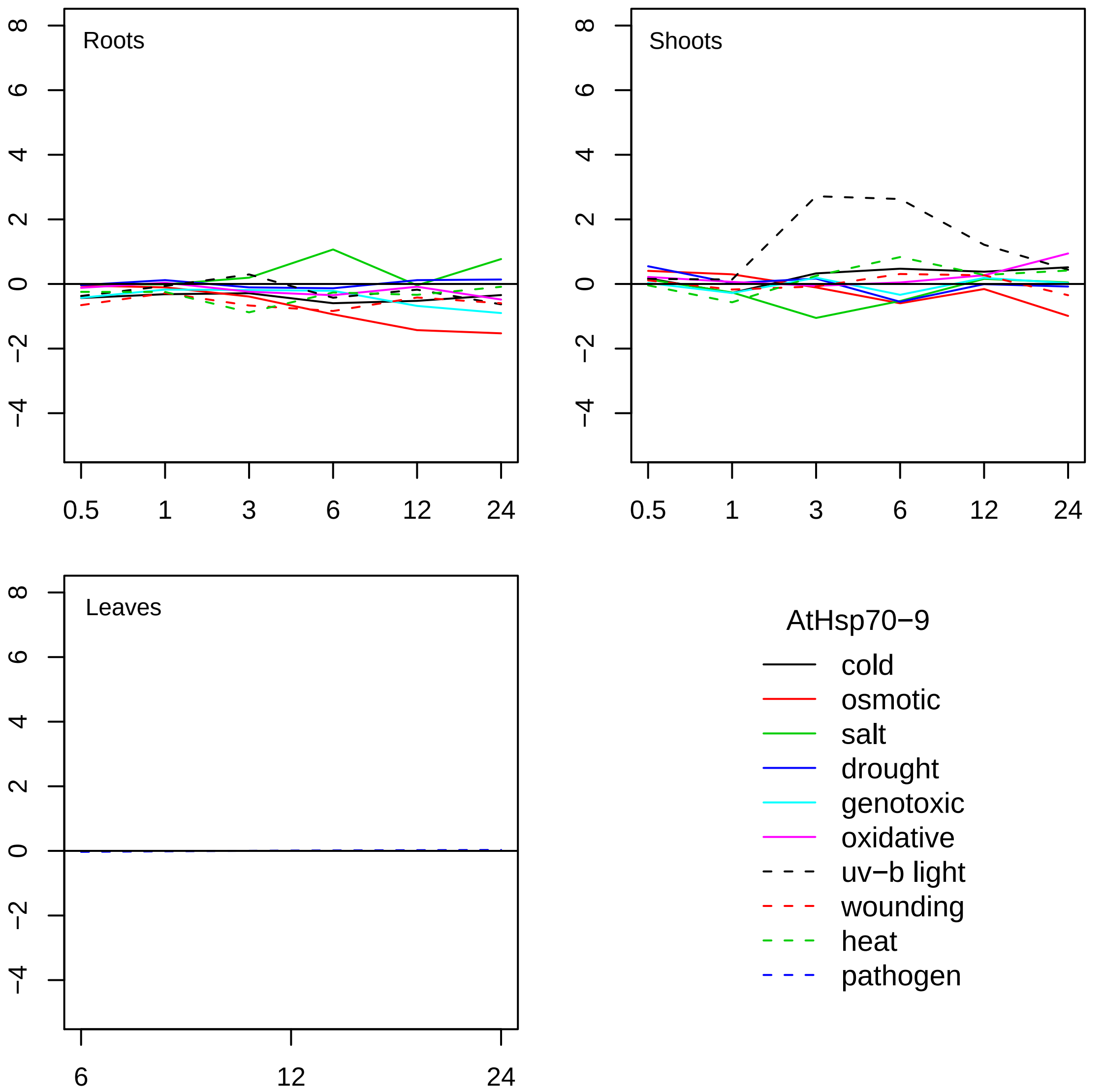

Roots
Shoots
Leaves
8
6
4
2
0
−2
−4
0.5
1
3
6
12
24
AtHsp70−9
cold
osmotic
salt
drought
genotoxic
oxidative
uv−b light
wounding
heat
pathogen

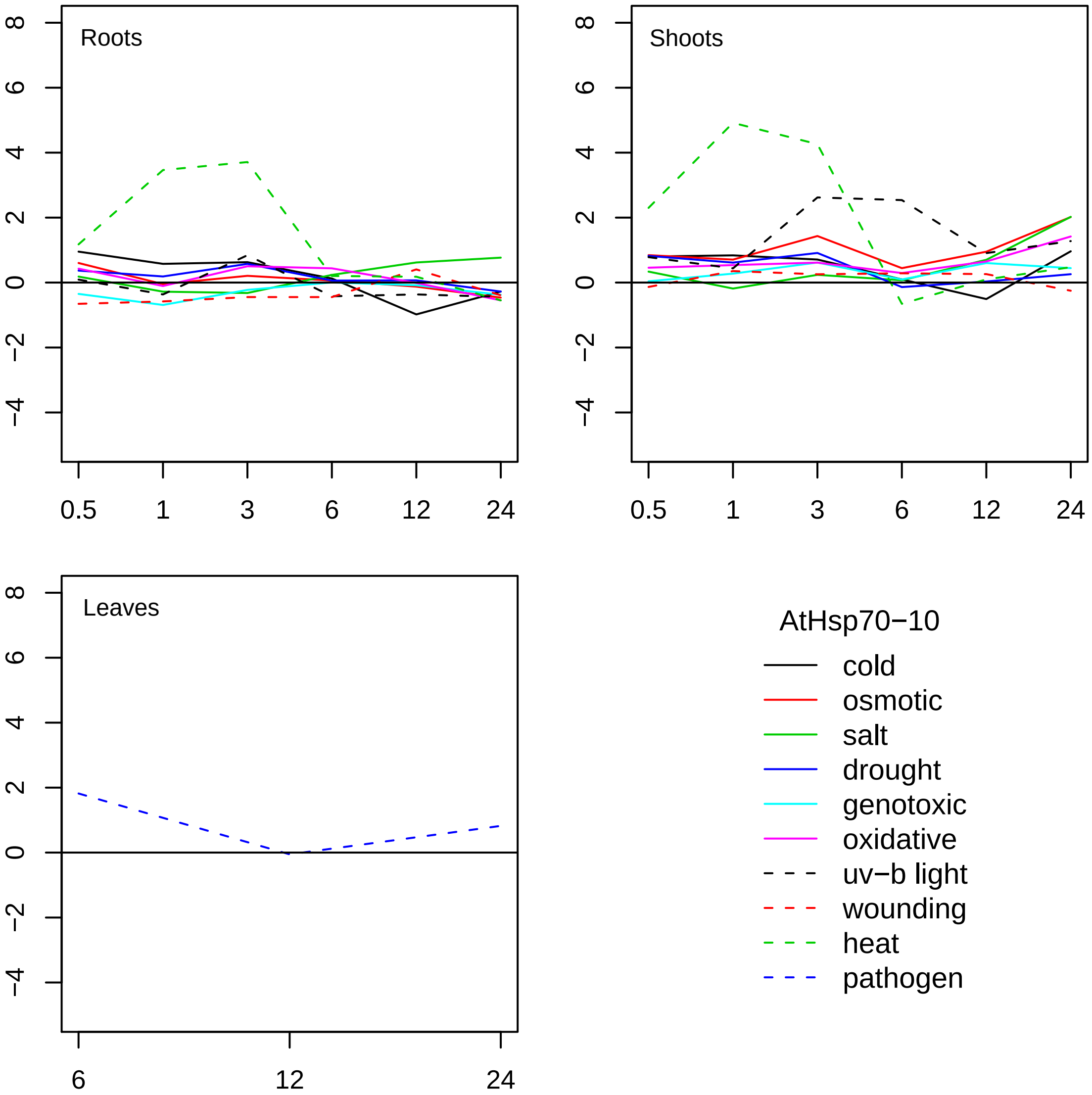
Roots
Shoots
Leaves
0.5
1
3
6
12
24
8
6
4
2
0
−2
−4
AtHsp70−10
cold
osmotic
salt
drought
genotoxic
oxidative
uv−b light
wounding
heat
pathogen

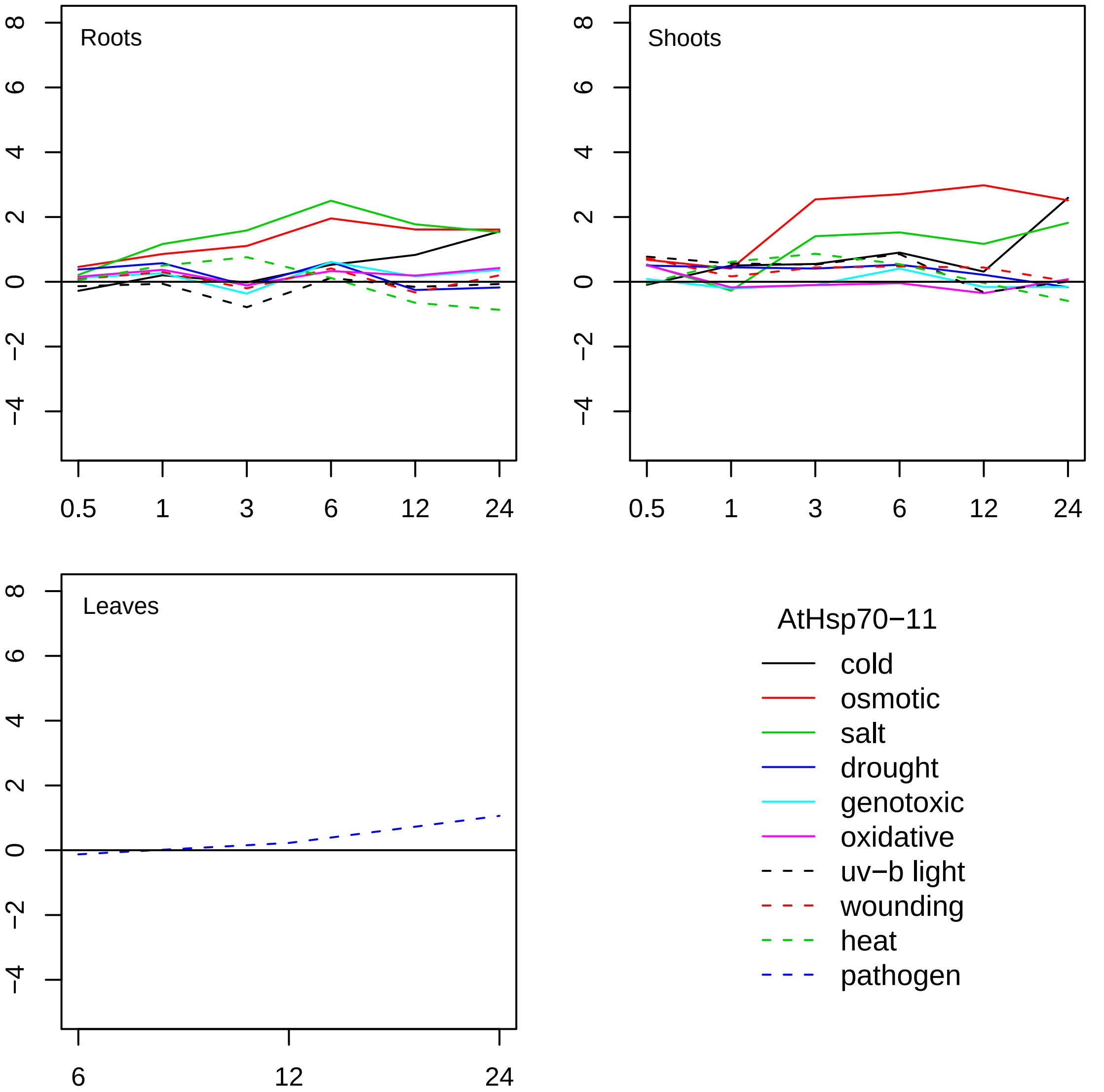
Roots
Shoots
Leaves
AtHsp70−11
cold
osmotic
salt
drought
genotoxic
oxidative
uv−b light
wounding
heat
pathogen
0.5
1
3
6
12
24
8
6
4
2
0
−2
−4

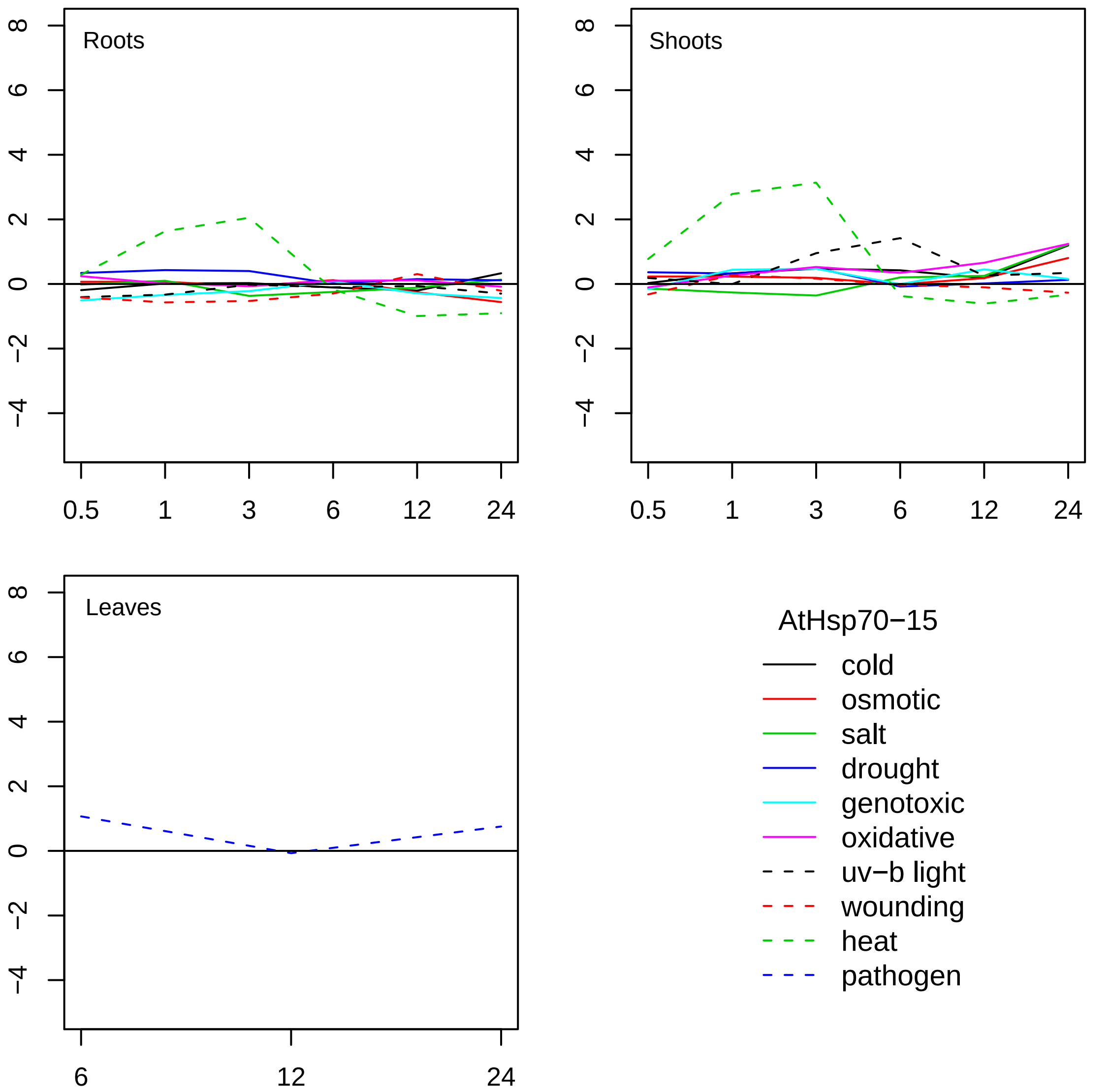

Roots
Shoots
Leaves
8
6
4
2
0
−2
−4
0.5
1
3
6
12
24
AtHsp70−15
cold
osmotic
salt
drought
genotoxic
oxidative
uv−b light
wounding
heat
pathogen

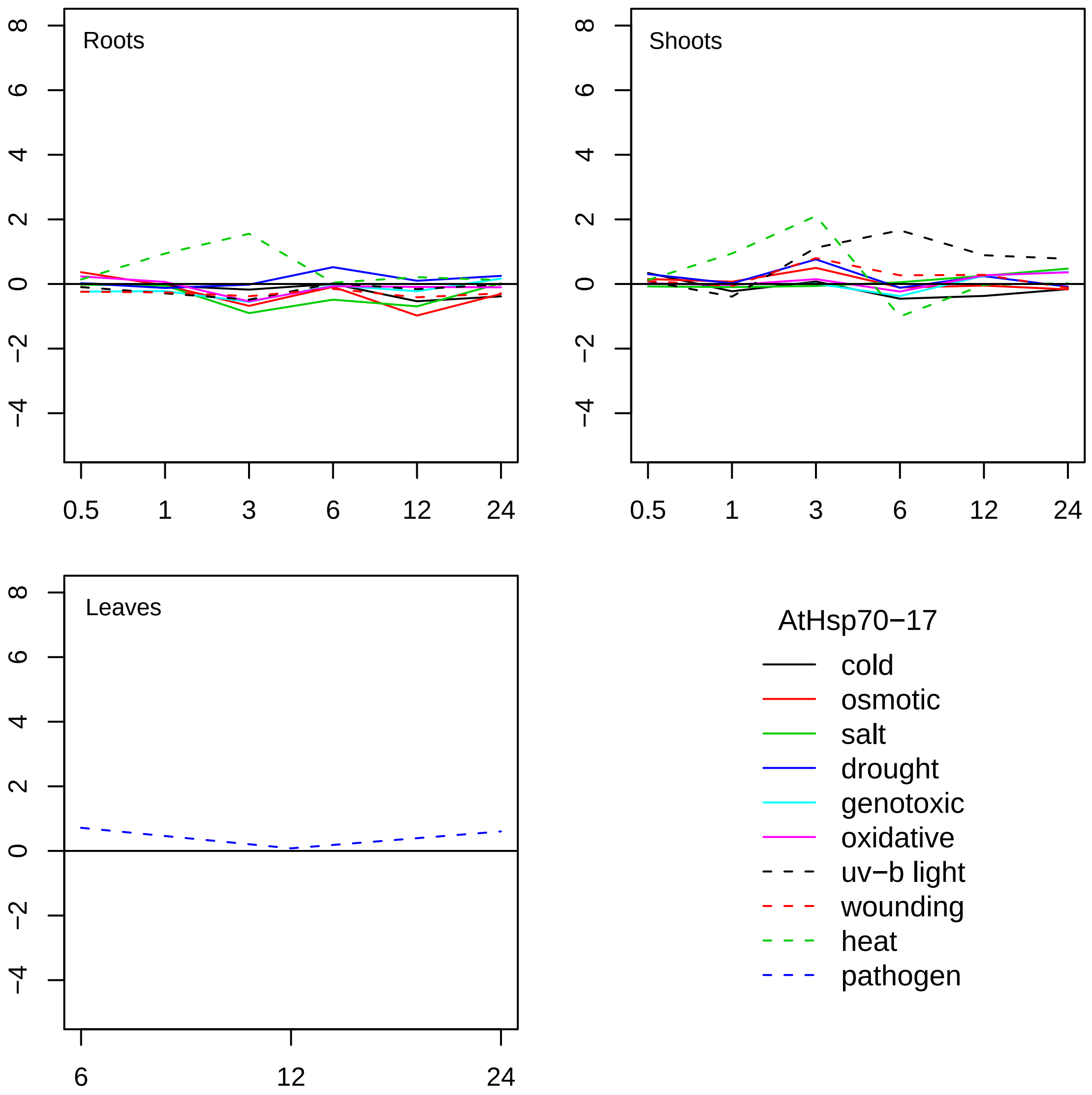

Roots
Shoots
Leaves
0.5
1
3
6
12
24
8
6
4
2
0
−2
−4
AtHsp70−17
cold
osmotic
salt
drought
genotoxic
oxidative
uv−b light
wounding
heat
pathogen

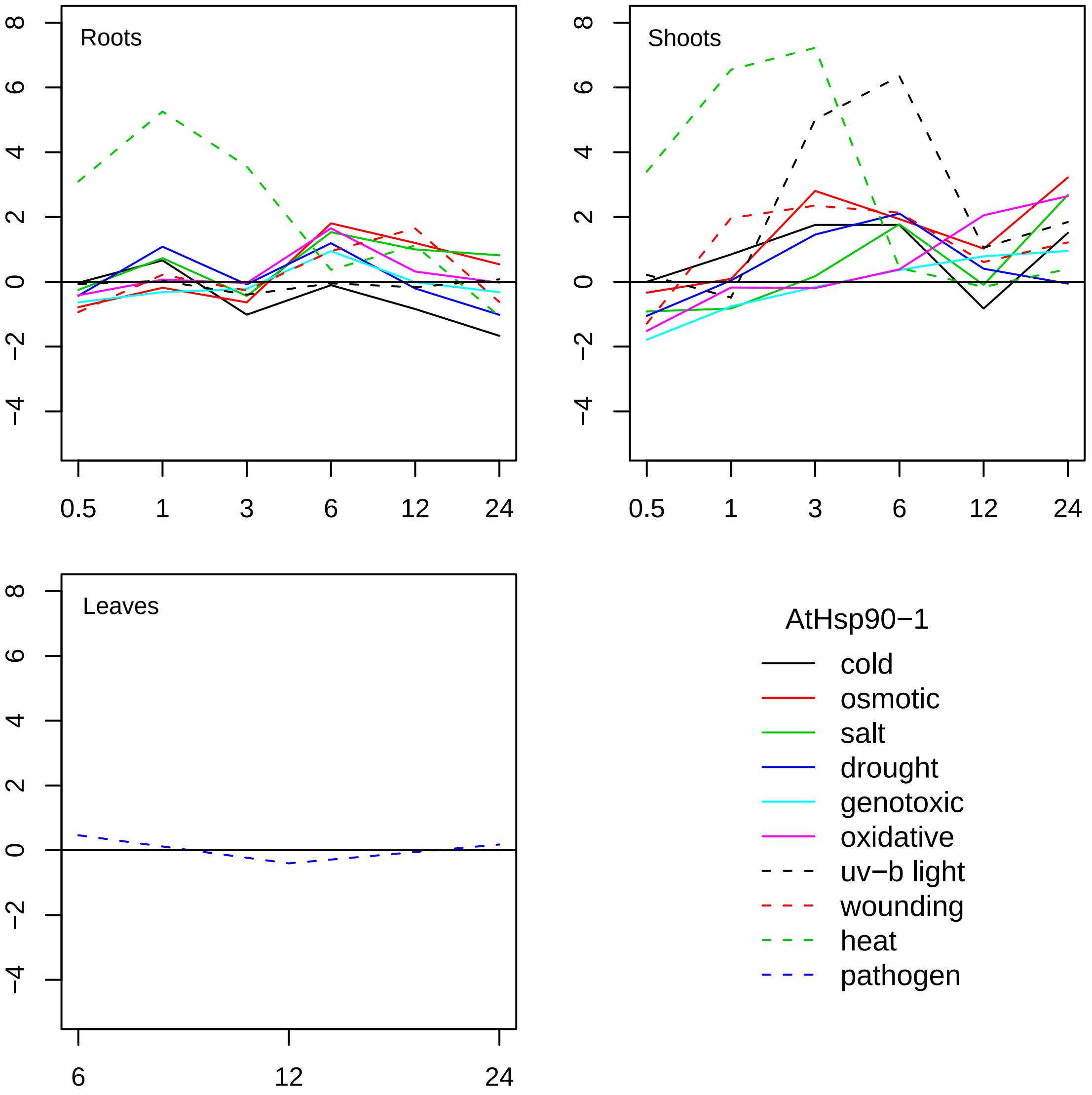

Roots
Shoots
Leaves
8
6
4
2
0
−2
−4
0.5
1
3
6
12
24
AtHsp90−1
cold
osmotic
salt
drought
genotoxic
oxidative
uv−b light
wounding
heat
pathogen

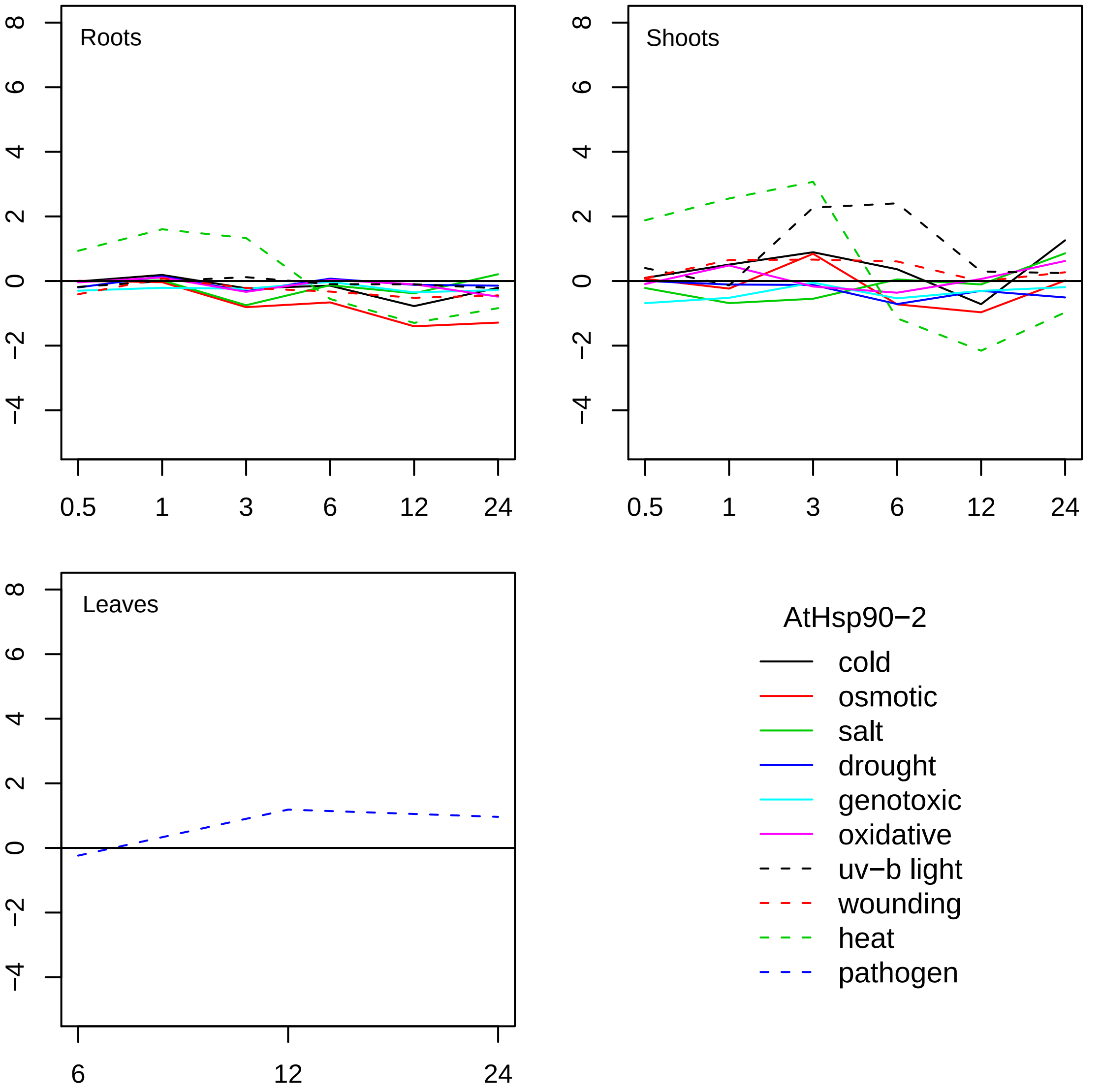

Roots
Shoots
Leaves
8
6
4
2
0
−2
−4
0.5
1
3
6
12
24
AtHsp90−2
cold
osmotic
salt
drought
genotoxic
oxidative
uv−b light
wounding
heat
pathogen

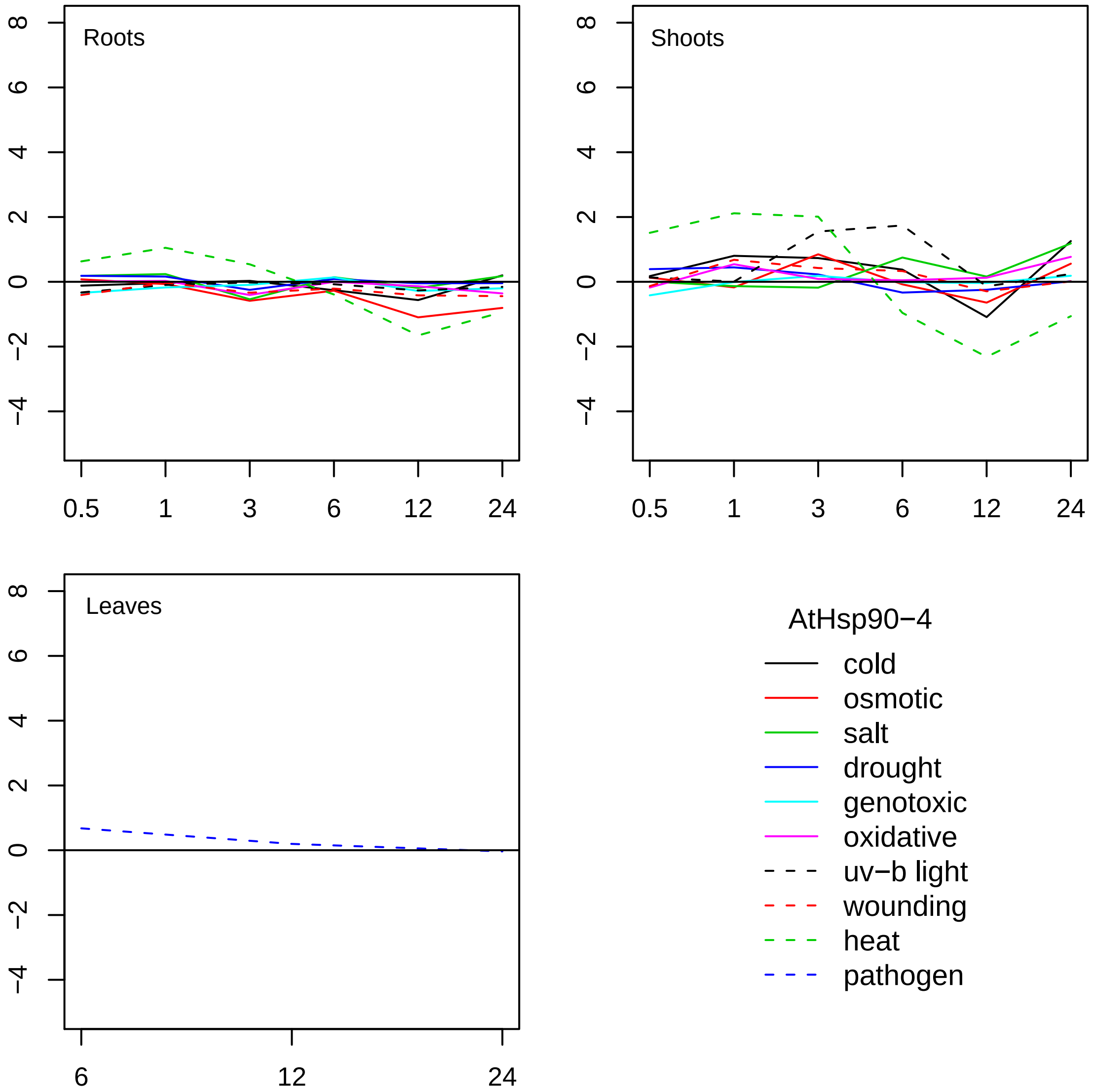

Roots
Shoots
Leaves
AtHsp90−4
cold
osmotic
salt
drought
genotoxic
oxidative
uv−b light
wounding
heat
pathogen

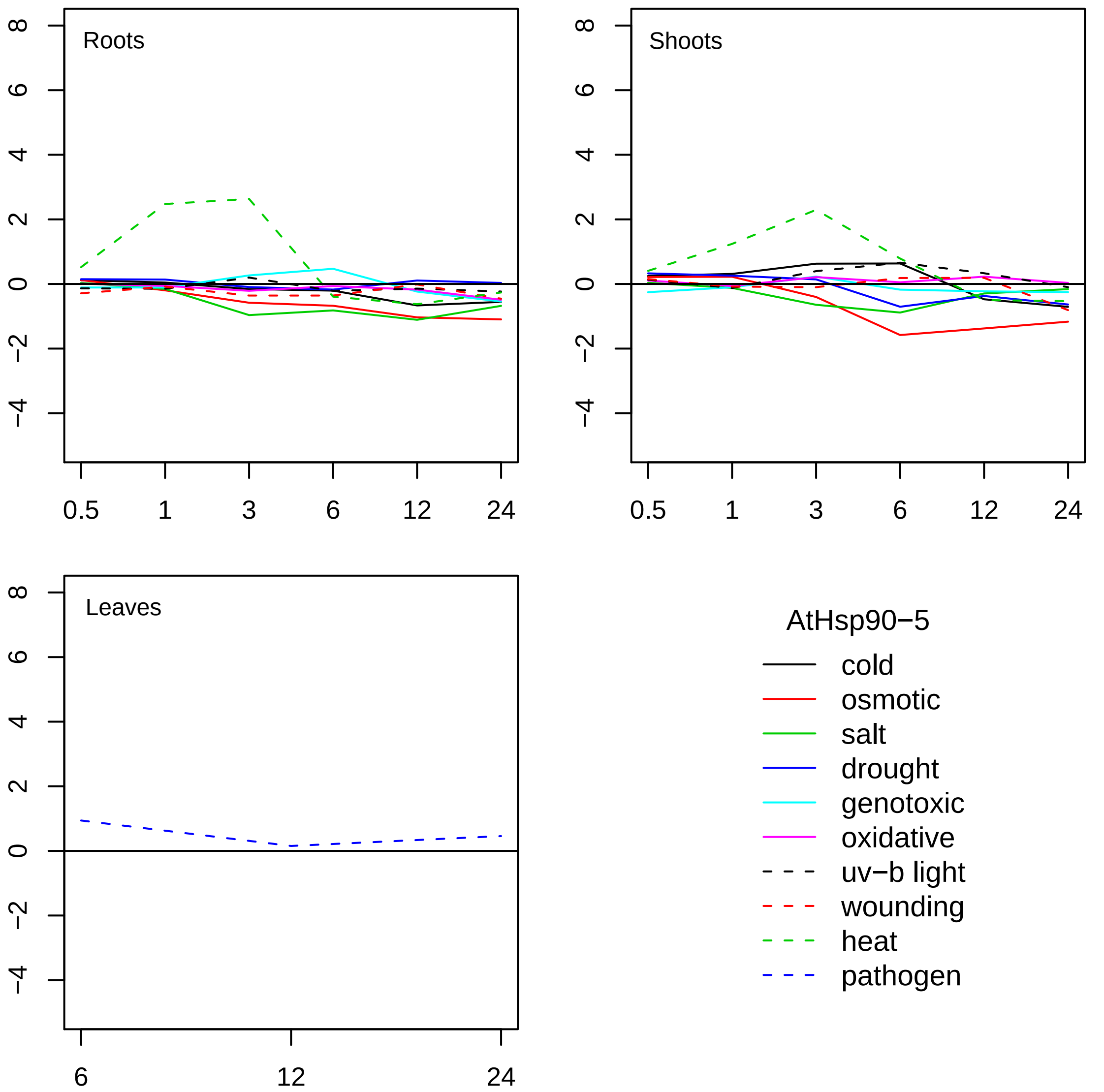

Roots
Shoots
Leaves
8
6
4
2
0
−2
−4
0.5
1
3
6
12
24
AtHsp90−5
cold
osmotic
salt
drought
genotoxic
oxidative
uv−b light
wounding
heat
pathogen

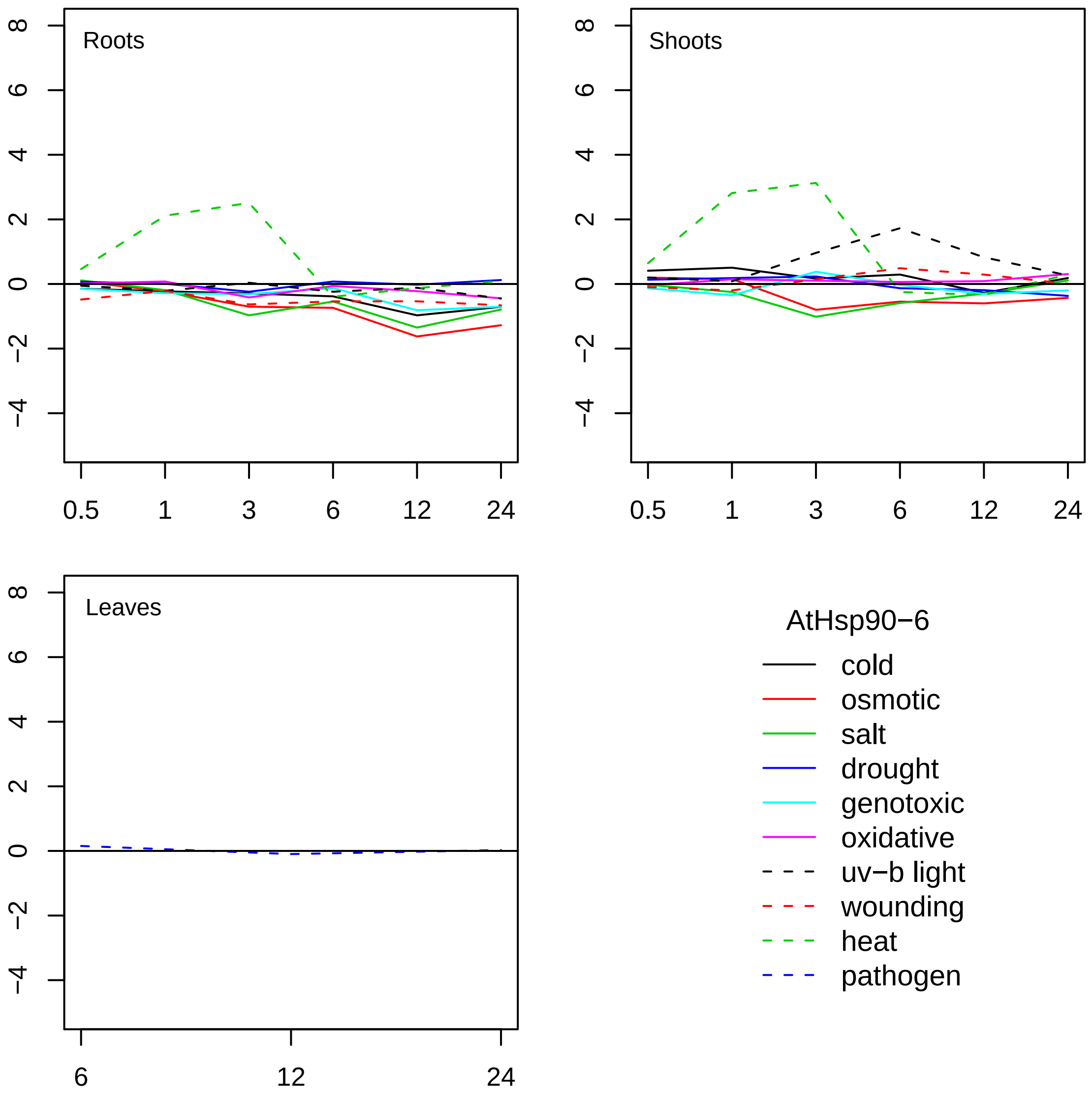

Roots
Shoots
Leaves
AtHsp90−6
cold
osmotic
salt
drought
genotoxic
oxidative
uv−b light
wounding
heat
pathogen
8
6
4
2
0
−2
−4
0.5
1
3
6
12
24

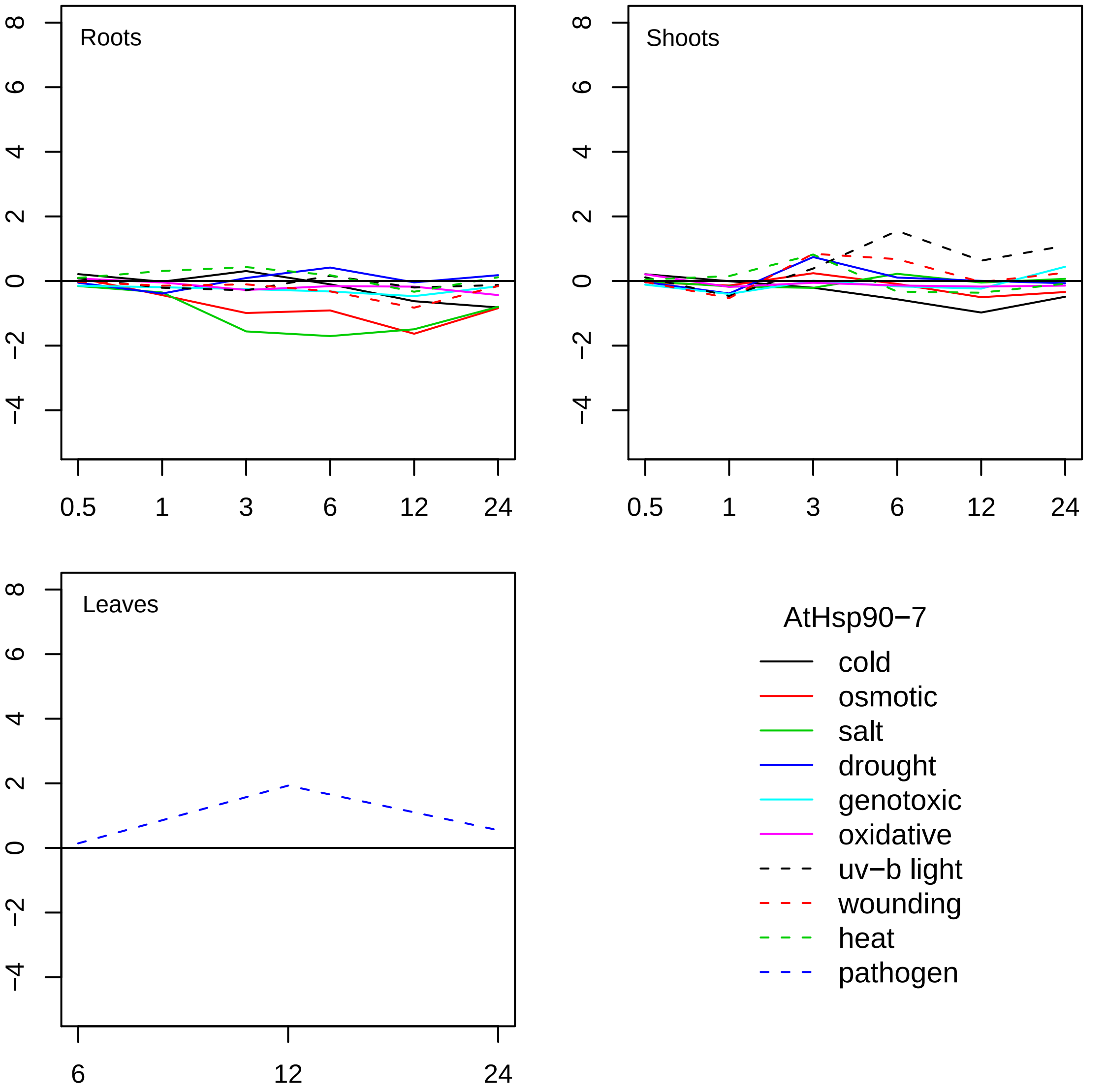
Roots
Shoots
Leaves
8
6
4
2
0
−2
−4
0.5
1
3
6
12
24
AtHsp90−7
cold
osmotic
salt
drought
genotoxic
oxidative
uv−b light
wounding
heat
pathogen

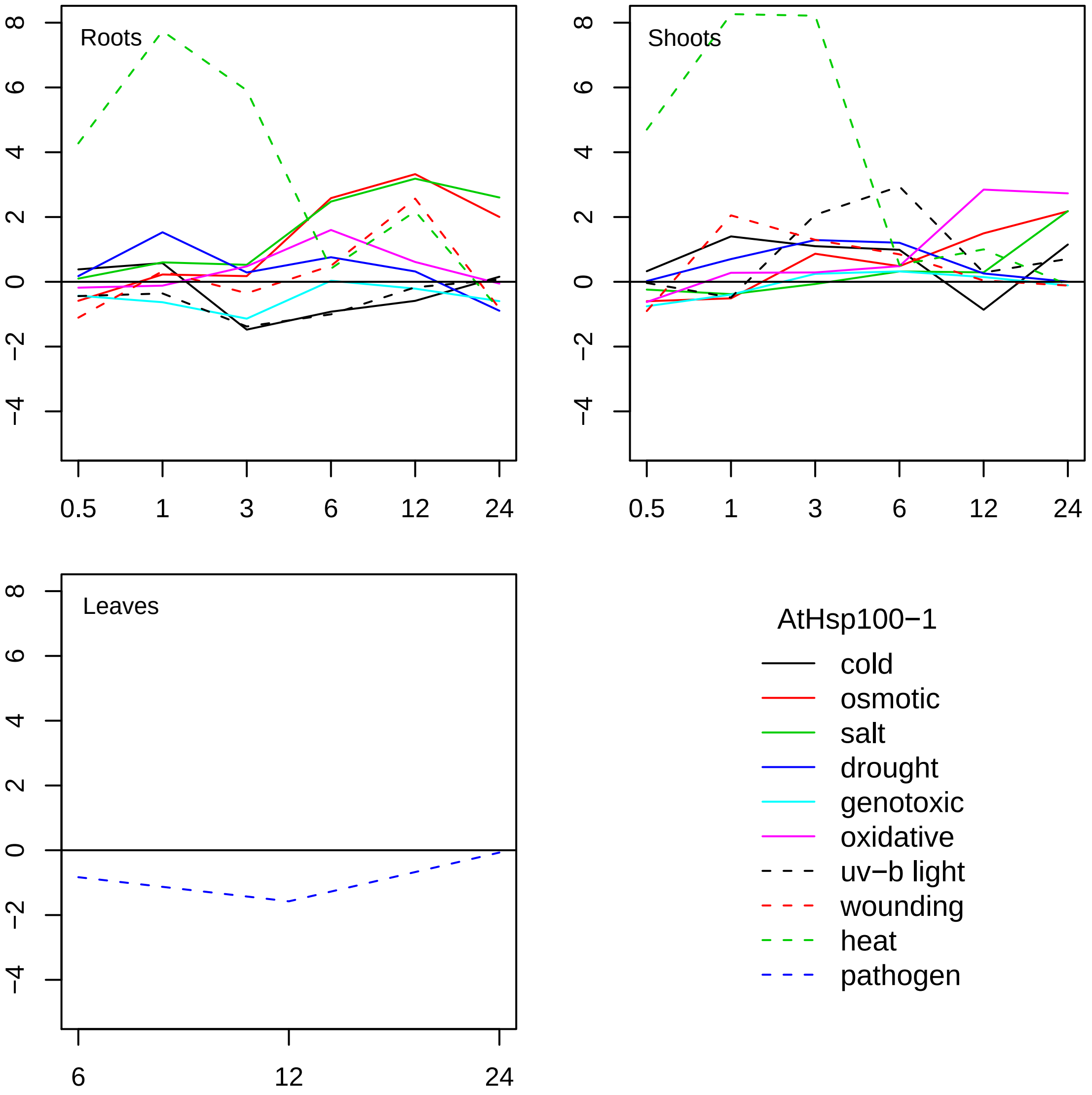
Roots
Shoots
Leaves
8
6
4
2
0
−2
−4
0.5
1
3
6
12
24
AtHsp100−1
cold
osmotic
salt
drought
genotoxic
oxidative
uv−b light
wounding
heat
pathogen

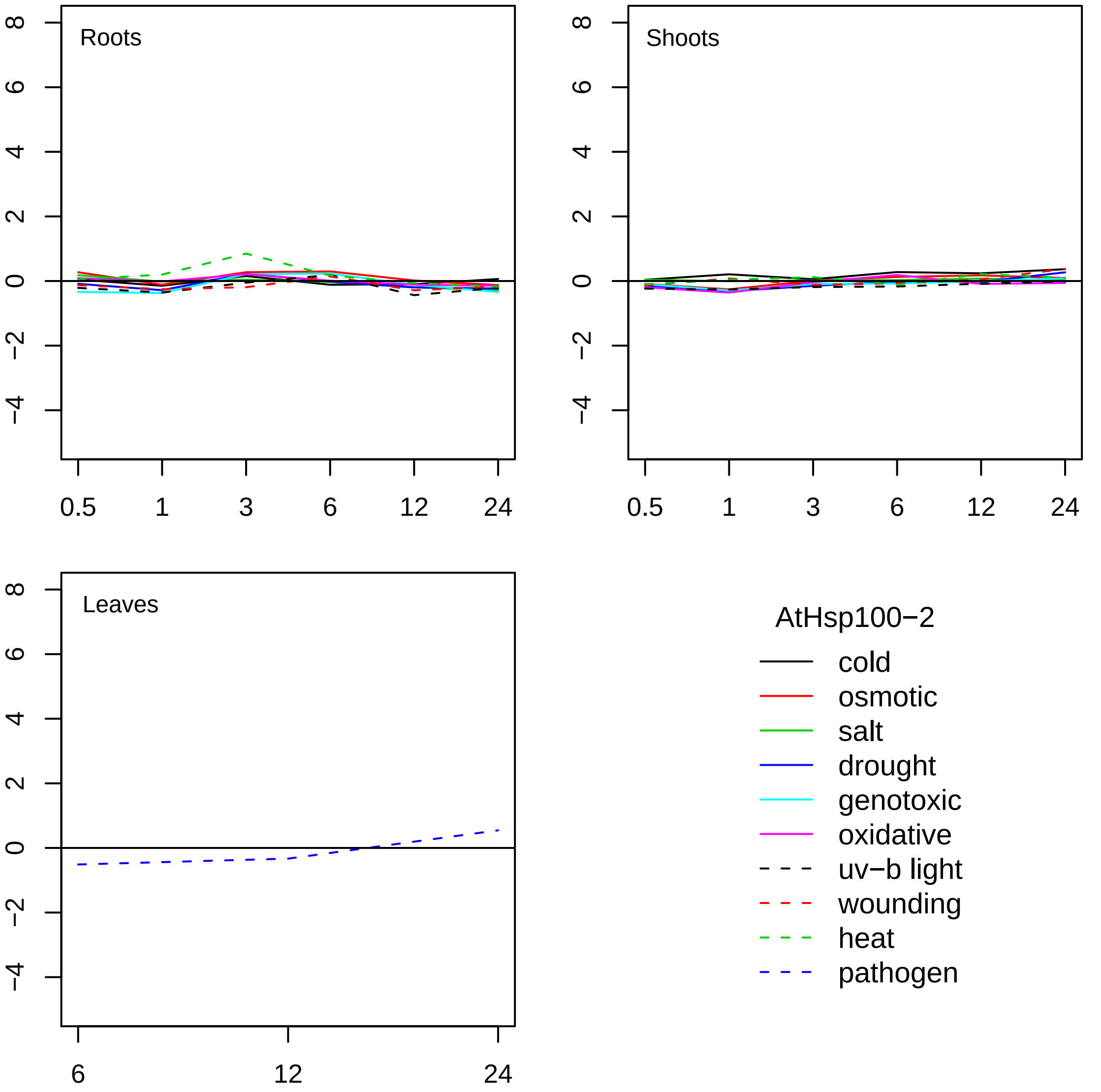

Roots
Shoots
Leaves
8
6
4
2
0
−2
−4
0.5
1
3
6
12
24
AtHsp100−2
cold
osmotic
salt
drought
genotoxic
oxidative
uv−b light
wounding
heat
pathogen

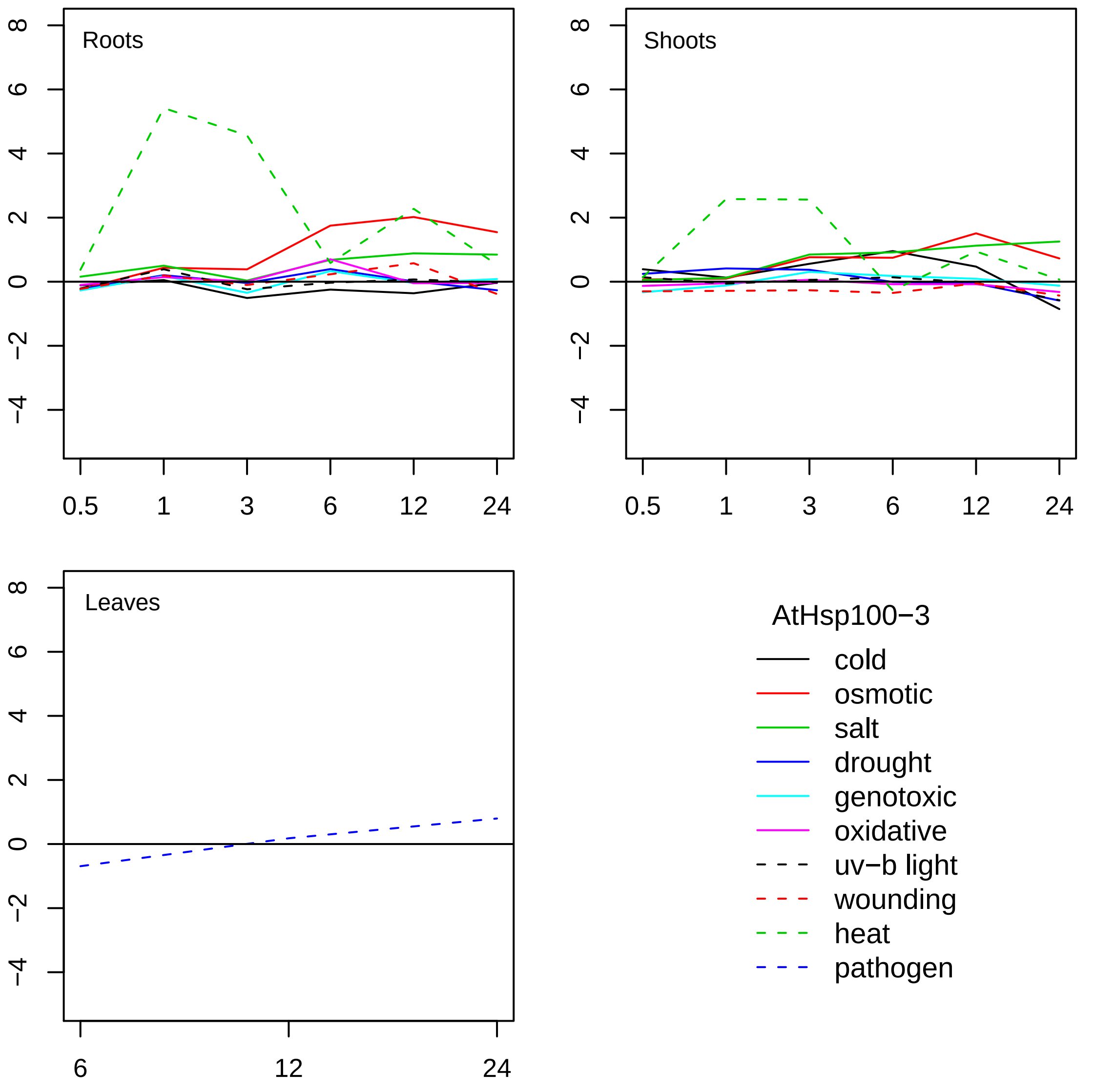
Roots
Shoots
Leaves
8
6
4
2
0
−2
−4
0.5
1
3
6
12
24
AtHsp100−3
cold
osmotic
salt
drought
genotoxic
oxidative
uv−b light
wounding
heat
pathogen

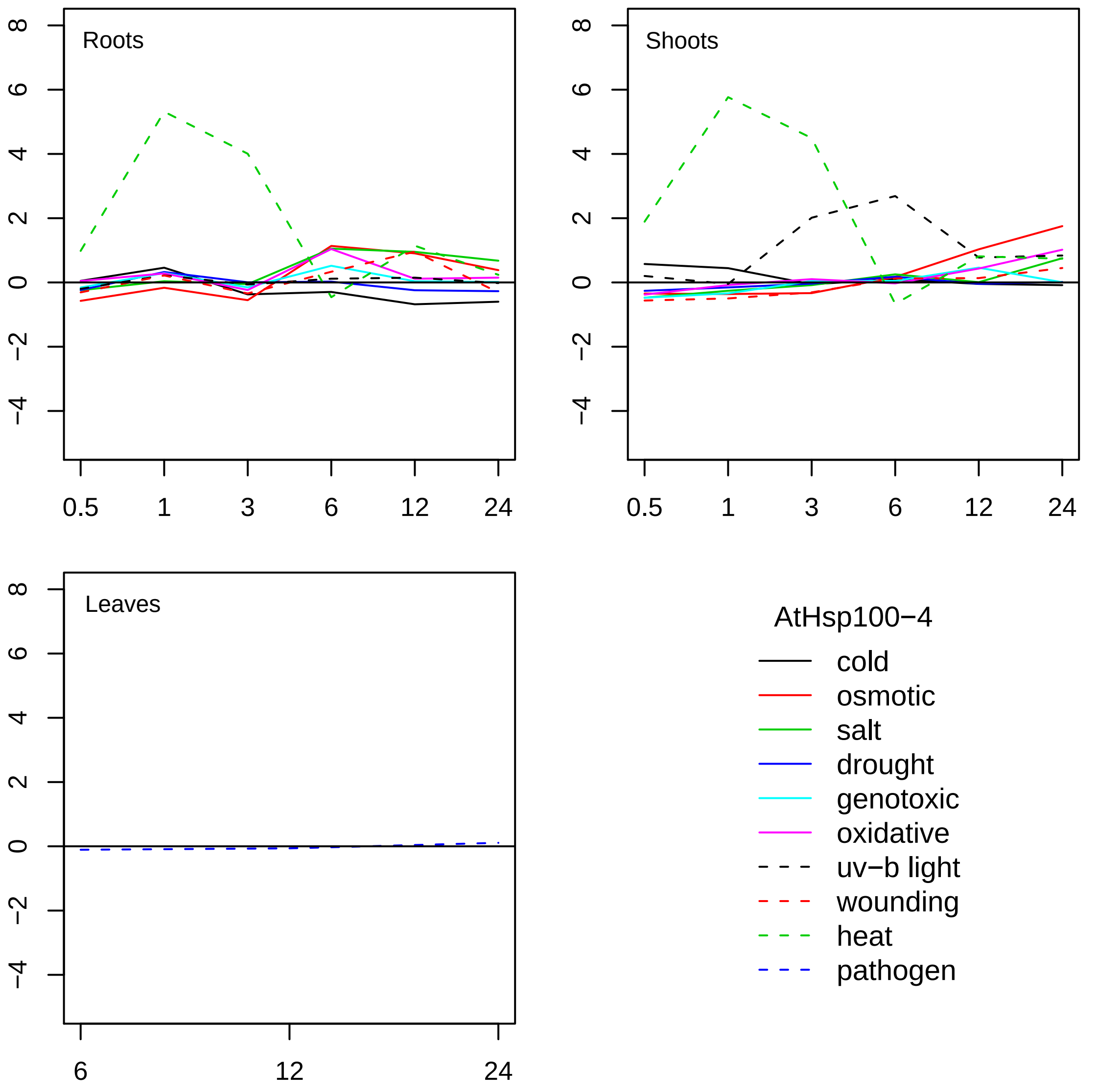

Roots
Shoots
Leaves
8
6
4
2
0
−2
−4
0.5
1
3
6
12
24
AtHsp100−4
cold
osmotic
salt
drought
genotoxic
oxidative
uv−b light
wounding
heat
pathogen

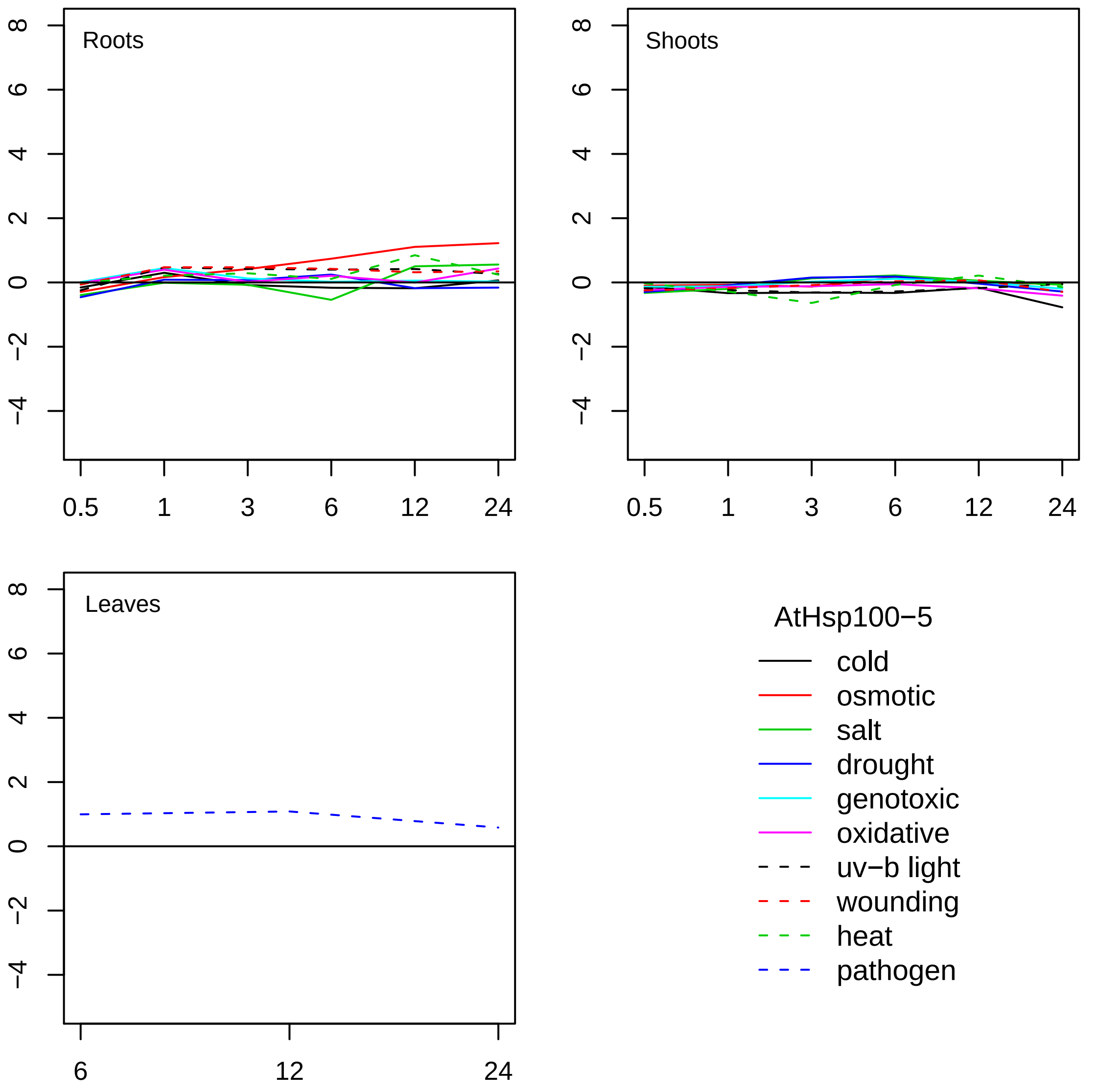
Roots
Shoots
Leaves
AtHsp100−5
cold
osmotic
salt
drought
genotoxic
oxidative
uv−b light
wounding
heat
pathogen
8
6
4
2
0
−2
−4
0.5
1
3
6
12
24

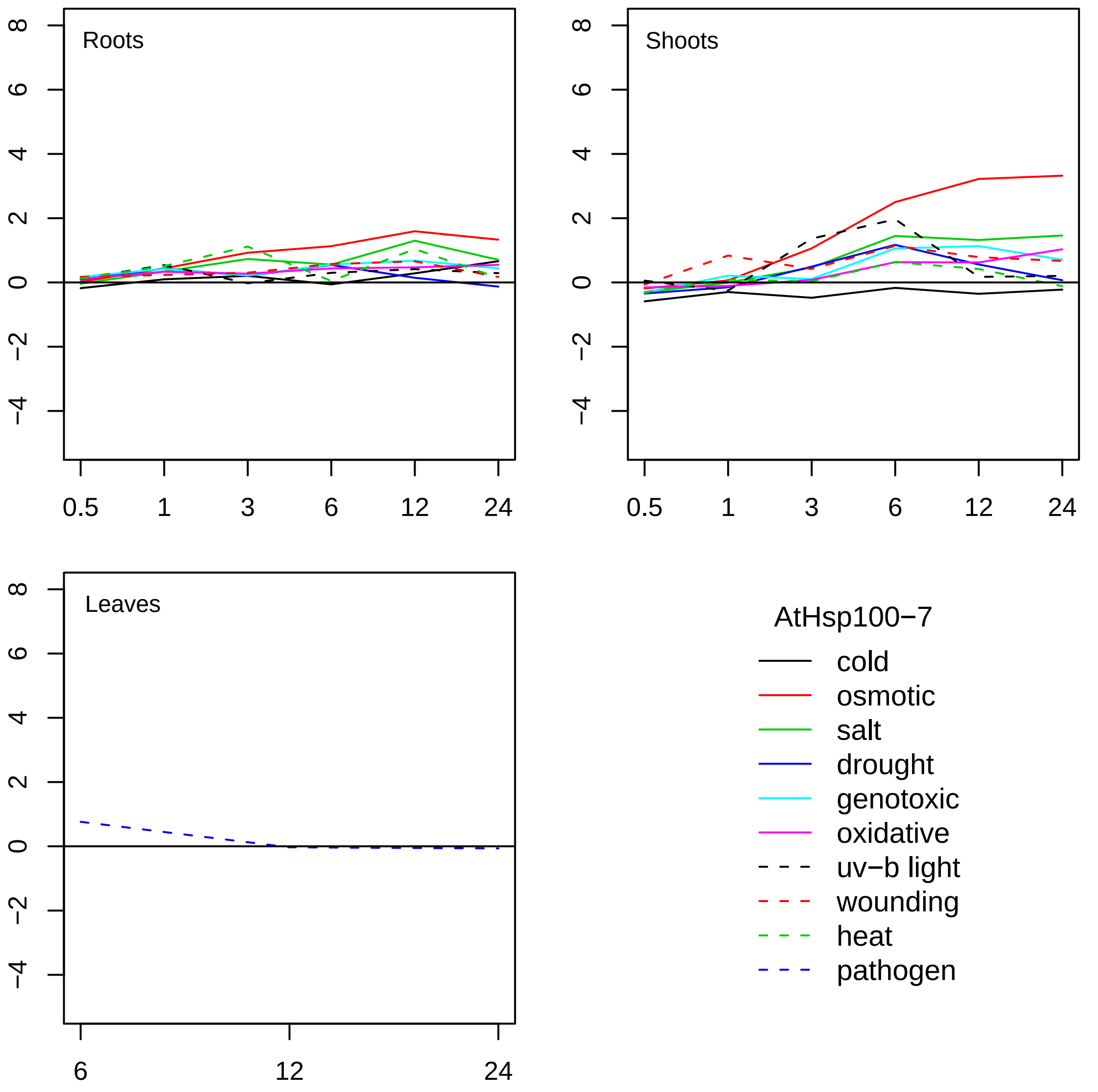

Roots
Shoots
Leaves
8
6
4
2
0
−2
−4
0.5
1
3
6
12
24
AtHsp100−7
cold
osmotic
salt
drought
genotoxic
oxidative
uv−b light
wounding
heat
pathogen

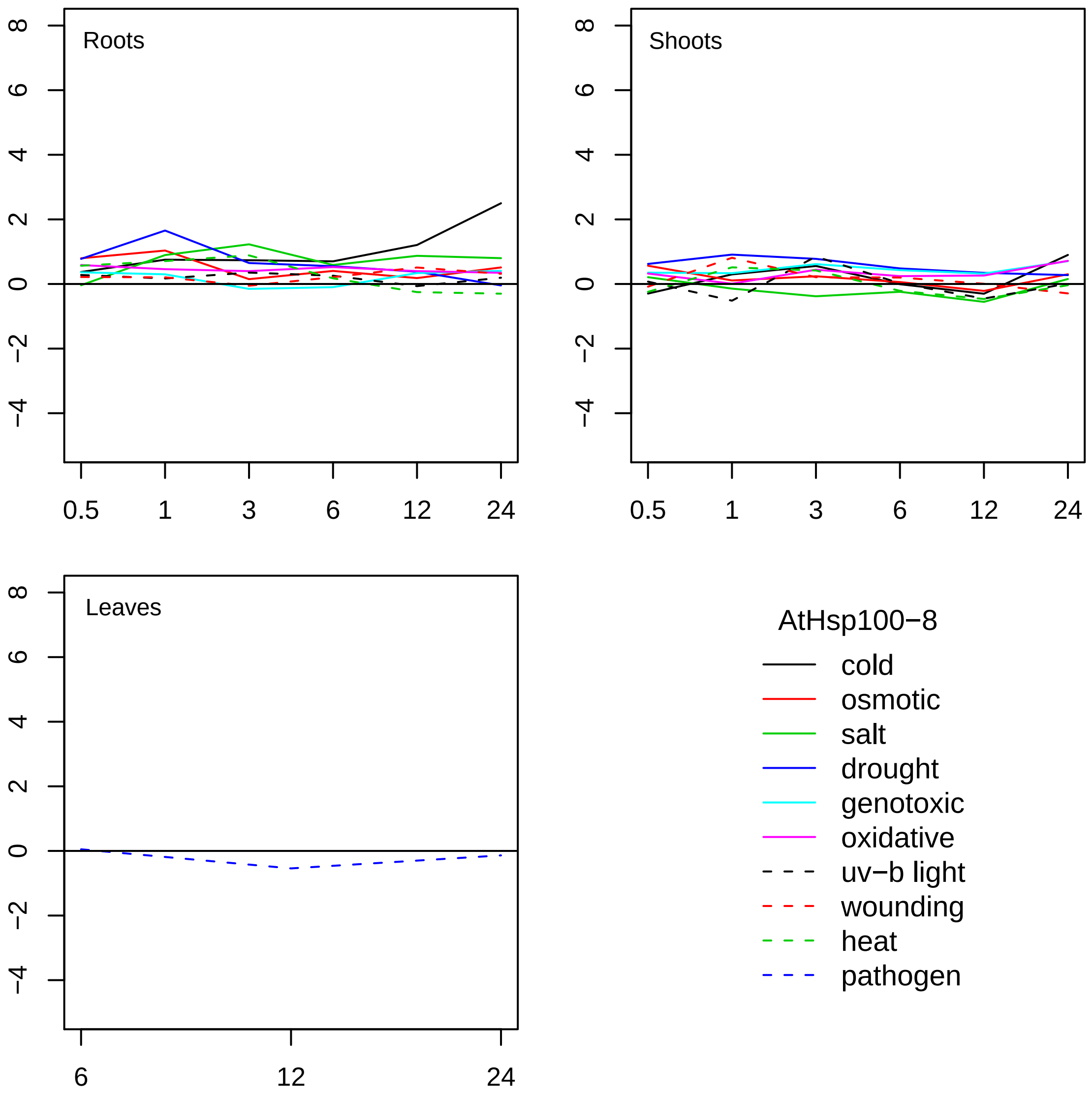
Roots
Shoots
Leaves
8
6
4
2
0
−2
−4
0.5
1
3
6
12
24
AtHsp100−8
cold
osmotic
salt
drought
genotoxic
oxidative
uv−b light
wounding
heat
pathogen
